# Stereoselective design of amino acid bioconjugates: targeting strategies and physicochemical optimization

**DOI:** 10.1039/d5md00760g

**Published:** 2025-12-24

**Authors:** Soma Mandal, Rajat Choudhary, V Badireenath Konkimalla

**Affiliations:** a School of Biological Sciences, National Institute of Science Education and Research, HBNI PO- Bhimpur-Padanpur, via- Jatni Khurda Odisha 752050 India; b Homi Bhabha National Institute Training School Complex, Anushakti Nagar Mumbai 400094 India

## Abstract

Amino acid conjugates are progressively becoming popular as a potent tactic to enhance the pharmacological efficacy of drugs, especially in the areas of cancer and antimicrobial therapy. By taking advantage of the intrinsic biological attributes of amino acids, their conjugates facilitate drug stability, selective accumulation, and enhanced therapeutic efficacies. In particular, the structural analogy of amino acids to physiological substrates enables these conjugates to use solute carrier transporters, commonly overexpressed in tumour cells, which allow for targeted and effective drug delivery. This review considers how amino acid properties like chirality, hydrophobicity and steric bulk can be modulated to maximize drug conjugates. We emphasize important design aspects, such as selection of linkers and coupling reagents, and how these have an impact on drug release and biodistribution. Specific focus is given to d-amino acid, which increases proteolytic stability and bioactivity for both anticancer and antimicrobial uses, and to l-amino acid, which is responsible for receptor recognition, metabolic compatibility and amino acid decorated nanoparticle formulation. The existing drawbacks of antibody–drug conjugates (ADCs) and peptide–drug conjugates (PDCs) are immunogenicity, enzymatic degradation and poor tissue penetration. Amino acid conjugates provide a strong rationale with higher chemical versatility and potential for better pharmacokinetics and less toxicity. By harnessing the insights from chemistry, transporter biology and therapeutic design, this review presents a strategy for the creation of next-generation amino acid conjugates that bridge molecular accuracy to clinical utility.

## Abstract 2

A stereochemistry driven overview of amino acid bioconjugates, outlining targeting startegies and physicochemical design principles that advance anticancer, antimicrobial and CNS-directed therapeutics.

## Graphical abstract

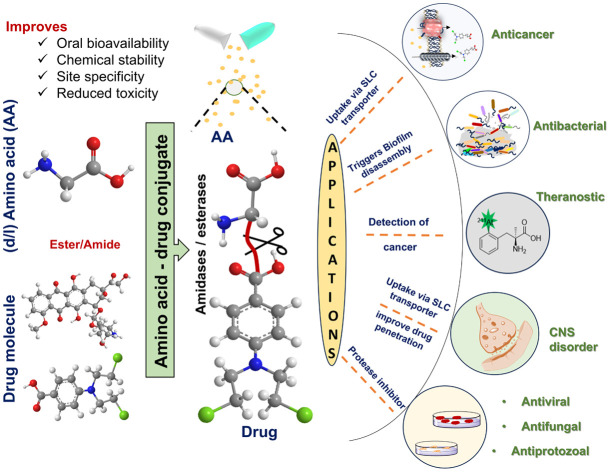

## 1. Introduction

Bioconjugation is a pivotal process in biomedical research, where drug molecules can be linked with biomolecules (amino acids, peptides, and carbohydrates), diagnostic agents, or targeting moieties that can enhance stability, proteolysis resistance, and targeting capabilities, thereby improving therapeutic efficacy.^1,2^ The design of a bioconjugate is primarily influenced by its intended function and the desired characteristics of the final product. A critical attribute for a successful bioconjugation is the selection of an appropriate cross-linker that ensures a stable and effective conjugate with desired features.^3^ Conjugating naturally occurring moieties with amino acids facilitates structural mimicry due to their involvement in vital biological pathways/systems, and the variations in the conjugated side chains confer considerable structural/property changes.^4^ Amino acid and peptide bioconjugates offer a promising avenue as delivery agents in targeting cancer cells with minimal activity loss, selective cytotoxicity, controlled drug release, low dosage requirements, and limited side effects.^5^ Drugs are transported passively to cancer tissues using nanocarriers in conventional therapy, relying on the increased permeability and retention effect caused by the aberrant blood artery architecture surrounding the tumors. Chemotherapeutic agents such as doxorubicin, methotrexate, and gemcitabine drugs are linked to carriers that contain ligands that bind specifically to overexpressed cancer cell receptors, resulting in high drug penetration within tumors, reduced systemic release, and prolonged circulation, with immunogenicity playing an essential role in ligand selection.^6–9^

Over the past few decades, significant advancements in cancer therapeutics have markedly prolonged cancer patient survival. Considering the limitations of conventional chemotherapy, current approaches are expected to address the shortcomings by targeting the overexpressing tumor-specific receptors such as folate receptors (FR), human epidermal growth factor receptor in breast cancer (HER2), and CD44.^10^ Different approaches such as monoclonal antibodies, antibody–drug conjugates (ADCs), small molecule peptides and their conjugates, non-peptide ligands, and oligos (siRNA/antisense) have been used as effective approaches for targeted therapy. Among these, ADCs are promising targeted immunotherapeutic agents that deliver cytotoxic drugs directly to tumor cells, but their clinical efficacy encounters obstacles, including low penetration capacity, immunogenicity, short half-life, low payload potency, and unstable linkage in systemic blood circulation.^11^ However, the clinical applicability of peptide–drug conjugate (PDC) therapeutics is constrained by their stability, quick renal clearance, and susceptibility to enzymatic degradation.^12^

In recent years, a number of targeted drug delivery systems have been designed to increase the therapeutic index of anticancer drugs to enhance tumor selectivity and reduce adverse effects. Biopolymer-based carriers, natural polymers like gelatin, collagen and zein, are of interest due to their biocompatibility, biodegradability and drug loading tunability. These materials have been engineered into hydrogels, films, or nanoparticles for sustained or stimulus-responsive drug release. Nanoparticle-based platform designed nanocarriers, such as liposomes, polymeric micelles, dendrimers, and inorganic nanoparticles, are promising for passive and active targeting by the enhanced permeability and retention (EPR) effect or ligand-mediated uptake. Despite substantial progress, many of these targeted drug delivery systems still face significant challenges of poor tumor penetration, high production cost, and complex pharmacokinetics.^13–16^ Such limitations necessiates intense research for development of next generation delivery strategies that are more selective towards tumor transporter uptake and biocompatible. Amino acid conjugates emerge as a promising therapeutic class that utilizes endogenous transporter pathways (SLC and PEPT1) to accomplish tumor-selective uptake. In exploring alternative therapeutic strategies, biological molecules, including amino acids, lipids, and carbohydrates, serve as carriers that facilitate the targeted delivery of drugs to specific sites.

Amino acids are notable for their simple chemistry with diverse functions, including hormone production, cell signalling, homeostasis, and antioxidant functions. They also affect several human physiological processes and have strong associations in conditions such as cancer, atrophic diseases, and sarcopenia.^17^ Given their pivotal role in numerous physiological and pathological processes, amino acids have attracted considerable attention in drug development. For a bioconjugation approach, the intrinsic reactivity of the targeted amino acid is chosen based on its acidity/basicity, electrophilicity/nucleophilicity and oxidation–reduction properties. Furthermore, the unique spatial geometry and accessibility of the amino acid attributed by the side chain, N-group and C-group, are critical for ester and amide bond formation in conjugation.^18^

Over the past few decades, outcomes from clinical research have shown that different amino acid supplements may be therapeutically effective in treating various diseases.^19^ The non-canonical amino acid (ncAA)-mediated techniques open new avenues for identification and synthesis of various conjugates, vaccines, and cell-based treatments.^20^ Amino acid–drug conjugates enhance pharmacokinetics by increasing biodistribution, improving absorption properties, displaying less toxicity, and exhibiting high physiological effects.^21^ The FDA (Food and Drug Administration) and European Medicines Agency (EMA) have approved amino acid-based (melphalan, flufenamide, odevixibat, difelikefalin, avacopan, and sotorasib) medications in recent years.^22^ Valine in the tramiprosate (ALZ-801) prodrug improves oral bioavailability and gastrointestinal tolerability in Alzheimer's treatment.^23^ Also, the fundamental mechanism of amino acid therapy is to restore the balance between amino acid metabolism and redox homeostasis, which is otherwise perturbed in disease conditions.^24^ However, the treatment efficacy of amino acid therapy in some disorders has controversial/non-conclusive results, where studies conducted in a larger population are critical to precisely evaluate the therapeutic effectiveness of the amino acid therapy.

Amino acid–drug conjugates are indeed a novel drug design and delivery approach; however, despite their great potential, their functionalities are not fully explored. This review presents a comprehensive and up-to-date overview of amino acid-based bioconjugates for anticancer, antimicrobial and anti-inflammatory therapies. This comprises addressing key limitations of ADCs and PDCs, followed by pharmacokinetic advantages of amino acids making them suitable for prodrug design. A special emphasis has been given to chirality, highlighting the way in which d- and l-amino acids impact drug stability and therapeutic efficacy. This review provides a new stereochemical paradigm, by comparing the distinct application of d- and l-amino acids in antimicrobial and anticancer therapies, and exploring their incorporation into targeted drug delivery systems, metabolic-based targeting and nano-medicine. This further highlights the growing potential of amino acid chirality in rational drug design and modern therapeutic development.

## 2. Roadblocks of antibody–drug conjugates (ADCs) and peptide–drug conjugates (PDCs)

Most conventional cancer chemotherapeutics often lack selectivity, resulting in toxicity to normal cells.^25^ At the beginning of the 20th century, Paul Ehrlich first proposed the concept of “magic bullets”, where certain compounds could selectively target cancer cells, laying the foundation for modern targeted therapies in cancer research.^26^ The target cancer cells can be identified by thoroughly assessing the specifically overexpressed receptors such as epidermal growth factor receptor (EGFR), folate receptors (FR), transferrin receptors (TfRs), CD44, solute carrier transporters (SLCs), and Mucin-1 (MUC-1), and target them sparing the normal cells to attain improved therapeutic efficacy.^10^ To overcome these limitations requires new approaches, such as drug conjugation strategies, ADCs, and PDCs, an intriguing class of next-generation targeted therapeutics enabling specific targeting and enhancing therapeutic outcomes. Compared with conventional drug therapy, ADCs, and PDCs follow the core benefits of delivering cytotoxic payloads selectively in cancer cells, enhancing efficacy with reduced side effects, and thus allowing better tumor penetration.^27^

### 2.1. Limitations of ADCs and PDCs

ADCs and PDCs, despite their advantages compared to conventional chemotherapy, face several challenges of poor stability, rapid renal clearance, limited oral bioavailability, drug resistance, and cost-effectiveness. Furthermore, immunogenicity, toxicity, and low penetration ultimately constrain their therapeutic window.^12,28^ Many PDCs fail in clinical trials due to the challenge of rendering rational conjugate designs into potent anticancer therapy.

#### 2.1.1. Immunogenicity

Administration of biotherapeutics elicits an immune response against recombinant therapeutic proteins (enzymes, cytokines, and mAbs), peptide modalities (lixisenatide and teduglutide), and oligos (mipomersen sodium).^29^ The heterogeneity of the drug payload during conjugation steps of ADCs containing an unconjugated antibody fraction limits the efficacy of ADCs in competitive binding for target antigens and triggers immunogenicity by a T-cell-mediated response.^30^ Anti-drug antibodies (ADAs) are developed against certain domains of ADCs, such as the linker, the cytotoxic payload, mAb-derived neoepitopes, and monoclonal antibody (mAb) epitopes. Since forming large ADC–ADA immune complexes enables uptake by non-target immune cells, potentially causing cytotoxic effects and impairing immune cell viability, developing ADAs against cytotoxic drugs raises significant safety concerns.^31^

Clinical administration of brentuximab vedotin (chimeric anti-CD30 conjugated with MMAE; Seattle Genetics) showed infusion reactions and an ADA incidence rate of 37% with the presence of an epitope/mAb neutralizing antibodies (NAb). Gemtuzumab ozogamicin (Wyeth/Ayerst), humanized anti-CD33 conjugated with calicheamicin, showed a response rate of 1.1% due to the involvement of an ADA epitope: calicheamicin/linker, with a transient shortness of breath reported as a side effect.^29^

#### 2.1.2. Toxicity

Conjugation of a lipophilic payload with an antibody increases mAbs hydrophobicity, impairs stability, and induces aggregate formation. ADC aggregation enhances off-target cytotoxicity in target-negative cells, mainly through FcγR activation. FcγR-blocking antibodies or Fc-engineering reduced this toxicity.^32^ ADCs exhibit complex pharmacokinetic properties, showing prolonged systemic circulation and elimination from the body compared to small molecules. The extended half-life of ADCs is attributed to toxicities, including hematotoxicity, hepatotoxicity, and gastrointestinal toxicity. Additionally, nephrotoxicity has been associated with the immune response induced by the antibody component.^33^ Tivdak® (tisotumab vedotin), an FDA-approved ADC for metastatic cervical cancer, despite its therapeutic benefits, shows toxicity like neutropenia, ulcerative keratitis, and peripheral neuropathy.^34^ On the other hand, PDCs have a short half-life because of their smaller size, facilitating penetration into the tumor site; however, non-specifically hepatic uptake results in dose-limiting toxicity.^35^ Lu-177 DOTA-TATE is an FDA-approved PDC for neuroendocrine tumors; it binds to malignant cells overexpressing somatostatin receptor type 2, accumulates within tumor cells, and delivers cytotoxic radiation. However, the presence of a conserved sequence FWKT (Phe-Trp-Lys-Thr) pharmacophore allows non-selective binding to other somatostatin receptors (somatostatin-14, somatostatin-28 and cortistatin-14), leading to toxicities in the kidneys and liver, and bone marrow suppression.^35,36^

#### 2.1.3. Low permeability

ADCs and PDCs often face challenges in penetration into tumor stroma due to their higher molecular weight (160 kDa and 2–20 kDa, respectively) compared to small molecules.^37^ Studies show that less than 1% of effector molecules reach target cells, with the highest estimate being 1.5%. The average drug-to-antibody ratio (DAR) in clinical ADCs is 3.5–4, resulting in low drug delivery to tumor cells.^38^ A significant challenge associated with PDCs is the difficulty of administration due to low bioavailability and stability issues, which limit their intravenous use and preclude oral administration.^35^

### 2.2. Advantages of amino acid conjugates over ADCs/PDCs

A comparative table has been prepared based on the literature cited in the preceding sections, summarizing key findings and highlighting that overcoming the above challenges requires developing novel bioconjugation strategies with amino acids, carbohydrates, and lipid molecules, enhancing pharmacokinetic properties and receptor specificity. Targeted drug delivery systems are designed to selectively deliver cytotoxic agents to tumor cells by exploiting tumor-specific surface receptors.

As mentioned in Table 1, amino acid conjugates are generally low in molecular weight in comparison with ADCs, enabling faster penetration into tumor cells. Their SLC mediated uptake enhances target selectivity and reduces the off-target effect. Amino acid conjugates exhibit a shorter half-life and faster clearance, reducing systemic toxicity. Considering all the above factors, amino acid conjugates could serve as a complementary alternative strategy to ADCs and PDCs.

**Table 1:** Comparison of amino acid conjugates with antibody drug conjugates

Properties	Amino acid–drug conjugate	Antibody–drug conjugate	Peptide–drug conjugate
Typical size	Small molecule or short peptide with amino acid promoiety	mAb (∼150 kDa) + linker + cytotoxic payload	Peptide (5–30 a.a) + linker/payload
Production	Low-moderate production cost	High manufacturing cost	Solid-phase peptide synthesis
	Simple chemical synthesis	Complex biological production	Scalable with moderate yield
Clinical adaptation	Early clinical or preclinical stage	Advanced (15 approved by the FDA) (*e.g.* trastuzumab emtansine and brentuximab vedotin)	Still emerging with 96 clinical trials and 1 FDA approved (Lu-177DOTA-TATE)
Toxicity	Lower systemic toxicity	High toxicity if off-target	Lower systemic toxicity
	Challenges in biodistribution and target specificity	Complex pharmacokinetics and pharmacodynamics	PDCs show intermediate biodistribution with predominant renal clearance unless chemically stabilized
Targeting	SLC transporter	Antigen receptor mediated	Receptor-mediated uptake

## 3. Properties of amino acids to enhance pharmacokinetics in prodrug design

Among biological molecules, amino acids are fundamental building blocks of macromolecules integral to various cellular activities and metabolic processes. Each amino acid possesses a distinctive side chain that confers unique physiochemical properties, classifying them into four categories: (i) electrically charged (positive or negative), (ii) polar uncharged, (iii) hydrophobic, and (iv) exceptional cases such as glycine (Gly), cysteine (Cys), and proline (Pro).^39^ Amino acid side chains often have a combination of more than one property that facilitates the development of therapeutics by addressing challenges related to solubility, membrane transport, efflux mechanisms, and the stability of drug molecules, including their half-life. By leveraging the specific interactions of amino acid side chains, it is possible to enhance the pharmacokinetic and pharmacodynamic profiles of the drug molecules, thereby improving their efficacy and stability in clinical applications.

**Fig. 1:**
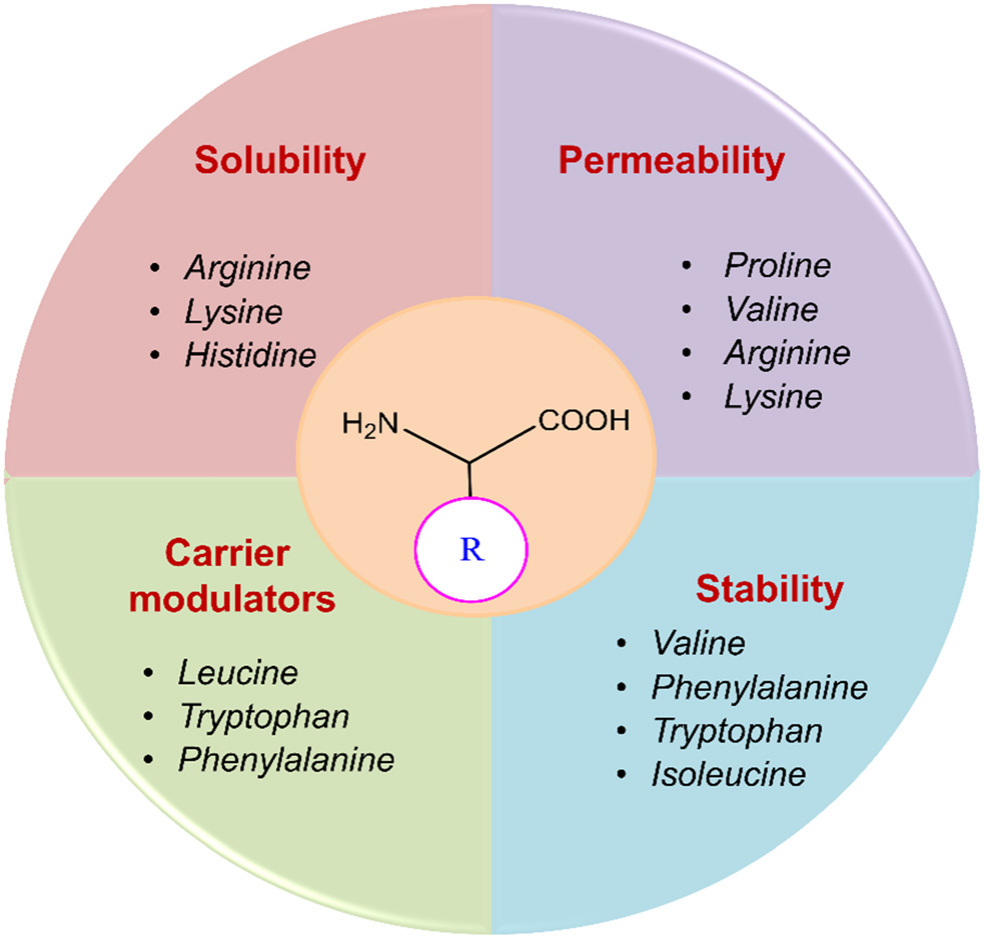
Advantages of amino acids as prodrugs.

Amino acids enhance the pharmacokinetic properties of active pharmaceutical ingredients (APIs). When an API exhibits acidic properties, it can form a salt with a base, thereby improving its solubility and dissociation rate. Salt formation is a critical process in the pharmaceutical industry for optimizing API solubility and bioavailability.^40^ Amino acids are particularly advantageous as co-formers due to their non-toxic nature and the diverse functional groups provided by their side chains, facilitating various formulation interactions.^41^ Basic amino acids, such as arginine (Arg) and lysine (Lys), possess amino groups in their side chains, which interact with acidic groups of APIs to form salts. When formulated with Arg, this interaction significantly enhances the solubility and thermal stability of indomethacin and ciprofloxacin.^42,43^ Amino acid prodrugs are engineered to foster high chemical stability and are transformed into the active parent drug enzymatically. The high chemical stability of the prodrug minimizes early conversion to the parent drug and increases its shelf life.^44^ Enzymatic activation promotes rapid and efficient conversion of the prodrug upon administration. Prodrugs based on ester and amide bonds are hydrolyzed by different hydrolases and acid- or base-catalyzed processes (Fig. 1).^45^

Enzymatic activation is a key factor for the therapeutic efficacy of an amino acid conjugated prodrug. Amino acid conjugates are designed to deliver active drugs in a controlled and site-directed fashion, frequently based on the expression of a tissue- or disease-selective hydrolytic enzyme. This approach not only enhances drug bioavailability but also maximizes the therapeutic efficacy index by reducing off-target effects. The most ubiquitous enzymes responsible for activation, including carboxyethyl esterases, peptidases and phosphatases, identify and cleave particular linkers including esters, amides, and phosphate esters. Esterases, especially carboxyethyl esterases CES1 and CES2, are widely expressed in the liver, intestines and plasma. These enzymes are involved in the catalyzation of amides and the ester linkage in amino acid conjugates.^46,47^ A prime example is valacyclovir, which is an l-Val ester prodrug of acyclovir. Valacyclovir is transported into intestinal cells by PEPT1-mediated transport and subsequently hydrolysed rapidly by CES2 to the active drug acyclovir. B. Yang *et al.* performed an *in situ* perfusion study using wild type and PEPT1 knockout mice, and the result showed that the jejunum uptake of valacyclovir increased by 82% (the *K*_m_ value is 10.2 mM). In *in vivo* mouse studies deletion of PEPT1 leads to reduce in *C*_max_ by 4.6 fold and 2.8 fold long *T*_max_ of acyclovir as compare to wild type mice.^48^ The second prodrug is irinotecan, which is activated by CES2 to release SN-38, a strong inhibitor of topoisomerase I. The kinetic parameter of CES2 for irinotecan has a *K*_m_ value in the low micromolar range, which indicates high affinity and effective activation at therapeutic concentrations.^49^

Drug delivery entails the migration of molecules with diverse hydrophilic and lipophilic properties through biological membranes, enabling efficient membrane penetration for targeted drug delivery. Amino acid prodrug strategies using overexpressed endogenous solute transporters in specific organs offer a promising method for enhancing targeted drug delivery.^50^ Among the several types of transporters, l-type amino acid transporters (LAT-1 and LAT-2) play a crucial role in the movement of large neutral amino acids (His, Ile, Leu, Met, Thr, Trp, Phe, Tyr, and Val) into cells from extracellular fluids, which in turn affects pharmacokinetics. The transport of hydrophobic amino acids from the blood to the brain is facilitated by LAT-1, a transmembrane protein abundantly expressed at the blood–brain barrier (BBB). Because of their strong affinity for LAT-1, l-Leu, l-Trp, and l-Phe act as natural substrates that pass across the BBB efficiently.^51^ Although free amino acids exhibit low permeability, they represent a promising class of permeation enhancers when structured into peptides with cell-penetrating capabilities or modified into amphiphilic derivatives. Amino acids provide multiple polar groups capable of hydrogen bonding, which is crucial for permeation enhancement. Specifically, amino acids like Arg and Lys exhibit higher permeability rates, making them valuable in pharmaceutical formulations to improve drug delivery and efficacy.^52^

**Fig. 2:**
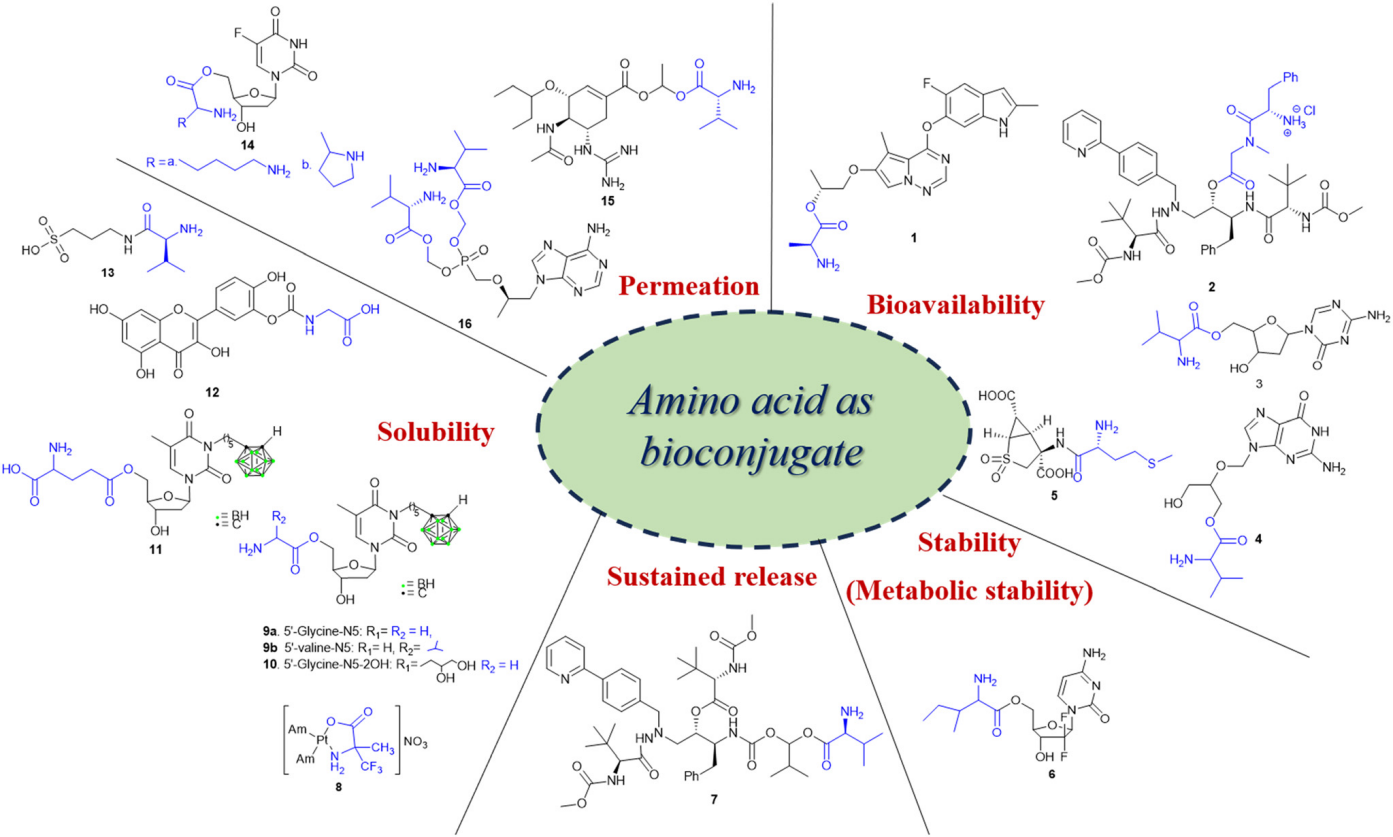
Amino acid conjugates to enhance pharmacokinetic properties.

Recently, small molecule drug conjugates (SMDCs) found a promising strategy for targeted cancer therapy, wherein small molecules act as ligands to selectively deliver cytotoxic agents to the tumor microenvironment, enhancing the therapeutic efficacy. Among other small molecules, amino acid conjugates are designed using principles analogous to those of ADCs and PDCs, yet their non-immunogenicity, reduced molecular weight, and easy synthetic methods render them highly effective in penetrating tumor cells, providing distinct advantages over ADCs in terms of delivery and therapeutic potential. Also, amino acid drug conjugates can be a promising alternative to PDCs, overcoming key limitations such as the off-target effect, low bioavailability, and poor stability.^53^ Amino acids play a pivotal role in different conjugations due to their unique structural properties, such as diverse side chains, reactive functional groups, and cysteine's thiol (–SH) group, which allows precise drug conjugation through methods like thiol–maleimide chemistry. These properties make amino acids valuable for bioconjugation in drug delivery and biomaterials. Examples of amino acid-conjugation structures and their pharmacokinetic properties are listed in Fig. 2 and Table 2. Thus, the diverse structural properties and stereospecific selection of amino acids should be meticulously considered in the design of amino acid conjugates to optimize therapeutic efficacy while minimizing systemic toxicity.

**Table 2:** Amino acid conjugated prodrugs

Purpose (properties)	Amino acid conjugate prodrug (structure #)	Amino acid	Transporter	Drug	Target	Screening (*in vitro* and *in vivo*)	Reference	Clinical trial status
Bioavailability	Brivanib–Ala conjugate (BMS-582664) (1)	l-Ala	Unknown	Brivanib	VEGFR, FGFR inhibitor	Liver, intestinal fractions, plasma, and Caco-2 cells	54	Phase I (NCT00437437), phase III (NCT00640471)
	Atazanavir–Phe conjugate (2)	l-Phe	Unknown	Atazanavir	HIV-protease inhibitor	Caco-2 cells and MT-2 cells (leukocytes)	55	—
	Decitabine–Val conjugate (3)	l-Val (high oral bioavailability), l-Phe, l-Trp	SLC15A1/PEPT1	Decitabine	DNA methyltransferase (DNMT)	Intestinal absorption in rats, Caco-2 cells	56, 57	—
	Ganciclovir–Val conjugate (valganciclovir) (4)	Val	PEPT1, PEPT2	Ganciclovir	Nucleoside analog	Rat renal proximal tubule cells (SKPT) and Caco-2 cells	58	Phase II (NCT00478465), phase II/III (NCT07079735)
	Pomaglumetad methionil conjugate (5)	Met	PEPT1	LY404039	Metabotropic glutamate 2/3 (mGlu2/3) receptor	HeLa transfected cells and schizophrenia patients	59	Phase II (NCT00845026)
Stability (metabolic stability)	5′-l-Isoleucyl conjugate (6)	l-Ile	PEPT1	Gemcitabine	Di- and tri-phosphorylated gemcitabine metabolites hinder the incorporation of CTP and dCTP nucleotides, causing nucleic acid synthesis termination	HeLa/hPEPT1 (recombinant PTP1 expressed), Caco-2 cells	9	Preclinical study
Sustained release	Atazanavir–Val conjugate (7)	l-Val	Unknown	Atazanavir	Azapeptide-based HIV-protease inhibitor	*In vivo* (SD-rat model), Caco-2 cells	60	—
Solubility	Platinum(ii) complex–Ala conjugate [Pt(NH_3_)_2_(α-Tfm-Ala)] (8)	Ala	Unknown	Cisplatin	Cell cycle arrest	HCT-116 cells (human colon cancer cells), A549 (NSCLC) cells	61	—
	Prodrugs of two 3-carboranyl thymidine analogs (3-CTAs) (9, 10, 11)	l-Gly (N5), l-Gly (N5-2OH), l-Glu (N5)	Unknown	3-Carboranyl thymidine analog	Double strand DNA break^62^	*In vitro* studies in bovine serum	63	—
	QC12 (3′-(*N*-carboxymethyl) carbamoyl-3,4′,5.7-tetrahydroxyflavone) conjugate (12)	l-Gly	Unknown	Quercetin	Induces late S/early G2 cell cycle arrest	A2780 (ovarian cancer cell line)	64	—
	Valine–tramiprosate conjugate (ALZ-801) (13)	l-Val	Unknown	Tramiprosate	β-Amyloid (Ab) anti-oligomer and aggregation	127 patient samples (phase I clinical study)	23	Phase II (NCT04693520), phase III (NCT04770220)
Permeability	Lysyl– and prolyl–floxuridine conjugates (14)	Pro, Lys	PEPT1	Floxuridine	Cell cycle arrest	PEPT1 overexpressed cells such as HeLa cells, Madin–Darby canine kidney (MDCK) cells, Caco-2 cells	9	
	Guanidine oseltamivir carboxylate (GOCarb)–l-Val conjugate (15)	l-Val	PEPT1	Oseltamivir	Neuraminidase inhibitor	Caco-2 cells, *in vivo* model (male Swiss Webster (CFW) mice, male albino Wistar rat)	65	Preclinical study
	Bis(l-valine) ester prodrug of tenofovir (TFV) conjugate (16)	l-Val	Unknown	Tenofovir	Inhibits replication of HBV	HepG2.2.15 cells (Hep G2 transfected with cloned hepatitis B virus DNA)	41	

### 3.1. Comparative pharmacokinetic properties of d- and l-amino acids

Both l and d-amino acids have been used in therapeutic design with applications ranging from antibacterial to anticancer strategies to targeted drug delivery. Despite structural resemblance, l- and d-amino acid differ substantially in pharmacokinetics shaping their clinical use. LAAs are well characterized due to their prevalence in nature; however, DAAs exhibit unique stability and clearance properties. The differences in their pharmacokinetic properties are mentioned in Table 3. This may guide rational therapeutic design in future. Incoporation of DAA can prolonge half-life and enhance metabolic stability; however, it may also alter tissue distribution and potentiate immunogenicity. These factors must therefore be carefully considered during therapeutic design.

**Table 3:** Comparison of pharmacokinetic properties of d and l-amino acids

Features	l-Amino acid	d-Amino acid	References
Absorption	• Recognized by intestinal amino acid transporters (LAT-1, PEPT1)	• Low absorption *via* receptors	66
	• Absorbed in the intestines and reabsorbed in renal proximal tubules, low urinary loss	• Reabsorption is less efficient	
	• Example: l-Ala complete renal absorption	• Faster clearance	
Proteolytic stability	• Rapid enzymatic degradation	• Longer persistence, resistance to proteases	67
	• Aminotransferase and dehydrogenase highly stereospecific for l-AA	• Cleared by d-amino acid oxidases (DAOs), produces α-keto acids	
Immunogenicity	• Less immunogenic	• Potentially immunogenic	68, 69
Half-life/stability	• Short half life	• Long half-life (resistance to proteolysis)	70
Chiral inversion	• Stable in mammalian systems	• d to l inversion *in vivo via* racemase enzymes	71
	• l to d inversion rare in humans	• Inversion influences pharmacokinetic and therapeutic effects of molecules	

## 4. Coupling reagents used in the synthesis of amino acid conjugates

**Fig. 3:**
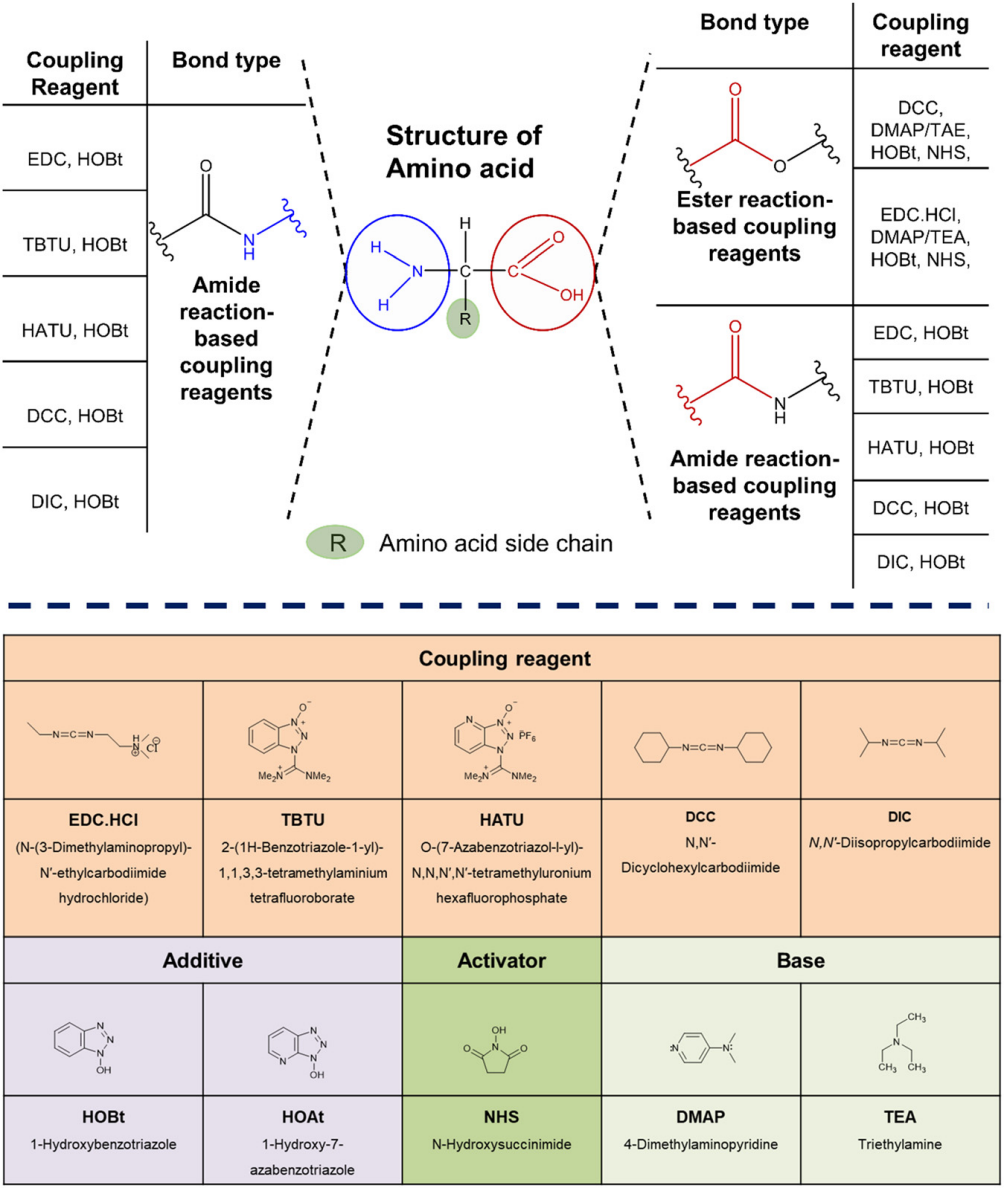
Structures of coupling reagents used in amino acid conjugation.

In amino acids, N- and C-terminals are reactive to facilitate the formation of an amide and ester bond in a bioconjugation reaction.^72^ Condensation of an amine with a carboxylic acid is the most prevalent process for forming amide bonds. Any residual reactive functional groups are usually protected to ensure that the carboxylic acid reacts efficiently with the amine. This reaction can be carried out in two steps: initially, the carboxylic acid is activated and isolated as a “trapped” intermediate, followed by its subsequent reaction with the amine (Fig. 3). Phosphonium, uronium, immonium, carbodiimides, imidazolium, organophosphorous, acid halogenating, and chloroformate chemicals are common peptide/amino acid coupling reagents.^73,74^

Aminium salts are incredibly effective peptide coupling agents that exhibit fast reaction times and low racemization. It is possible to eradicate racemization by adding an additive like HOBt. Aminium reagents are given to the carboxylic acid at equal molarity to prevent excess reagents from reacting with the peptide's free amine and prevent coupling. When phosphonium salts interact with carboxylate, two equivalent bases like DIEA are typically needed. As phosphonium does not react with the free amino group of the amine component, using phosphonium salts has several advantages over using iminium reagents.^75,76^ Various amino acid conjugates were synthesized using the microwave irradiation method using K_2_CO_3_ in anhydrous DMF.^77^ Basic amino acids like l-His, l-Arg, and l-Lys are often conjugated using coupling agents such as HATU and DIPEA.^78–80^

## 5. Chirality of amino acids

**Fig. 4:**
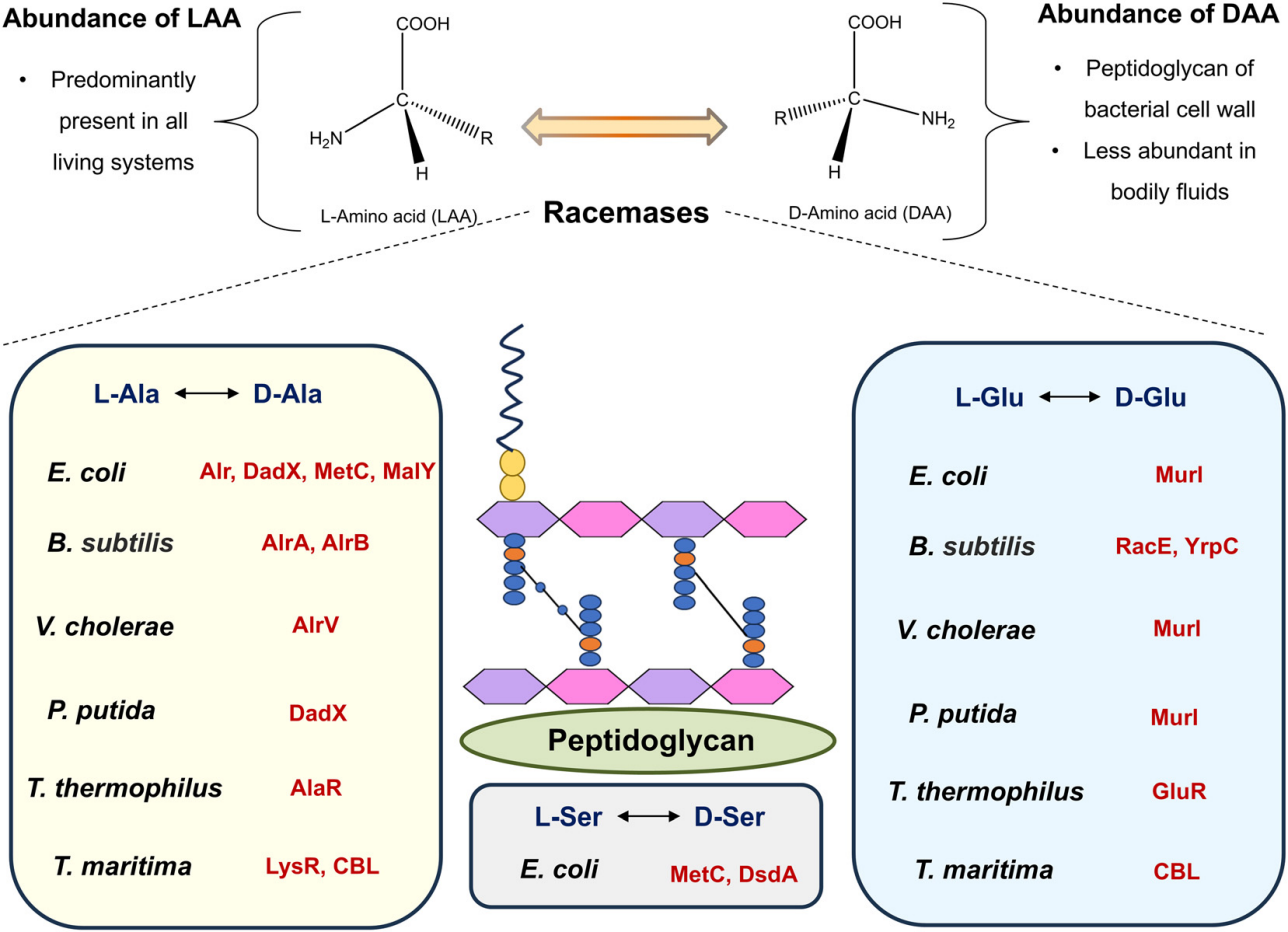
Abundance and interconversion of d- and l-amino acid stereoisomers facilitated by racemases in bacterial systems (abbreviations: *Escherichia coli* (*E. coli*), *Bacillus subtilis* (*B. subtilis*), *Vibrio cholerae* (*V. cholerae*), *Pseudomonas putida* (*P. putida*), *Thermus thermophilus* (*T. thermophilus*), and *Thermotoga maritima* (*T. maritima*)).

Stereoisomers in naturally occurring biological molecules have different spatial arrangements of atoms and are classified as d- and l-isoforms based on their structure (Fig. 4). In biological systems, chirality acts as a fundamental property that influences numerous physiological and biological processes. All twenty of the most prevalent proteogenic amino acids except glycine display chirality.^81^

Eukaryotic cells predominantly use l-amino acids (LAAs), l-phospholipids, and d-nucleotides, whereas bacterial cell walls contain d-amino acids (DAAs), which contribute to post-translational modifications and resistance to proteases.^82^ Although they are chemically indistinguishable, LAAs were evolutionarily chosen for the formation of peptides and are thus most prevalent in contemporary proteins. DAAs like d-serine (d-Ser), d-alanine (d-Ala), and d-aspartate (d-Asp) have been found recently in human neuroendocrine tissues, cerebrospinal fluid, and blood plasma.^83^d-Ser has been elevated in brain tissue in Alzheimer's disease condition and the level is correlated with β-amyloid plaque accumulation.^84^ Though mammals primarily utilize LAAs for physiological processes, imbalances of LAAs have been associated with metabolic disorders such as liver disease.^85^ DAAs like d-Ser (≥200 nmol g^−1^ wet tissue of the rat frontal brain region), d-Asp (rat pituitary gland at >3000 nmol g^−1^ wet tissue), and d-Ala (rat pituitary gland at >3000 nmol g^−1^ wet tissue) have been found in mammalian tissues like the brain, pituitary gland, and pancreas at levels up to ≥3000 nmol g^−1^, indicating their physiological functions. Their occurrence in neuroendocrine systems and biological fluids is indicative of diagnostic and functional relevance in NMDA-mediated neurotransmission and hormone-stimulating gene expression.^86,87^ Substituting l-peptides with d-peptides increases proteolytic resistance, which enhances *in vivo* stability and increases the circulation half-life, rendering d-peptide-based drug delivery systems more biostable and efficacious.^88^

In synthetic peptide chemistry and peptide drug design, the chirality of amino acids plays a significant role. It also markedly influences whether an amino acid will be used in protein synthesis or in regulating biological processes. For example, LAAs are proteogenic and often act as precursors for synthesizing DAAs. The change in the stereochemistry from LAAs to DAAs is catalyzed by a group of racemase enzymes, such as pyridoxal-5-phosphate (PLP)-dependent and PLP-independent enzymes.^89^ Bacteria adapt to environmental threats by synthesizing DAAs, predominantly through amino acid racemase, which catalyzes d- and l-stereoisomer interconversion. A diverse range of racemases from the isomerase family, involved in metabolic processes, have been identified in bacterial systems. These include His racemase (YgeA, MalY, & BsrV), cystathionine β-lyases (MetC & MalY), Lys racemase (LysR), Ala racemase (Alr & DadX), Glu racemase (GluR, RacE, YrpC, & MurI), and Ser racemase (MetC) (Fig. 4).^90^ These racemases play a major role in clinical antibiotic resistance mechanisms, for example overexpression of alrA (d-alanine racemases) in *M. tuberculosis* leads to d-cycloserine resistance, and alrA catalyzes the conversion of l-Ala to d-Ala which is an important component of cell wall peptidoglycan synthesis. In vancomycin resistance strain *Enterococcus* spp., VanT (ser/ala racemase) provides d-Ser for peptidoglycan remodeling. However, resistance in *Enterococcus faecalis* and *Enterococcus faecium* is mediated by the VanA and VanB gene clusters, which produce enzymes such as d-Ala-d-Lac ligases and alanine racemase (Alr). These enzymes alter the d-Ala-d-Ala end of peptidoglycan precursors to d-Ala-d-Lac, lowering vancomycin binding affinity by 1000-fold.^91–93^

Amino acids are very effective and less likely to cause bacterial resistance, and thus are employed to synthesize antimicrobial peptides, drug adjuvants, drug excipients, drug solubility enhancers, and anti-biofilm agents, among other significant uses. Also, the distinct stereochemistry of DAAs and LAAs influences their interaction with biological receptors and enzymes. Substituting DAAs with LAAs enhances their proteolytic stability and increases their plasma half-life. For example, selepressin exhibits a longer half-life than vasopressin, even though both target the same receptor.^94^ Since LAAs are more prevalent in biological systems, most human enzymes and receptors are adapted to recognize and process LAAs. In contrast, proteolytic enzymes are less likely to act on DAAs, thereby contributing to the increased stability of DAAs in biological systems.^95^ Moreover, incorporating DAAs creates resistance to enzymatic degradation, as proteolytic enzymes such as aminopeptidases exhibit reduced affinity for DAA-containing peptides. A practical application of this principle has been reported, such as modifying somatostatin to octreotide by incorporating DAAs for treating conditions like gastrointestinal tumors.^96^ This modification of stereochemistry significantly enhances the stability of the peptide and exemplifies how stereochemical adjustment can optimize peptides for better therapeutic efficacy and safety under *in vivo* conditions.

## 6. Role of d-amino acids in therapeutic design

### 6.1. Role of d-amino acids and biofilm disassembly

Bacteria produce diverse DAAs which are essential for peptidoglycan synthesis, metabolism, spore germination, and other physiological processes.^97^ Peptidoglycan cell wall synthesis follows the MurA-F pathway, where murein catalyzes the formation of oligopeptide substituents of *N*-acetylmuramic acid residues. Targeting the murein enzyme involved in peptidoglycan biosynthesis makes it an interesting target for antibacterial drugs.^98^

The peptidoglycan's peptide portion contains canonical amino acids, including d-Ala, d-Glu, and d-Gln, while l-ethionine, and *meso*-diaminopimelate are non-canonical amino acids.^99^ Alternatively, d-Ser and d-Asp are also found in peptidoglycan at the 5′ position of the stem peptide in vancomycin-resistant *Staphylococcus aureus (*VRSA) and *Enterococcus gallinarum*, creating resistance to bactericides (such as vancomycin). d-His and d-Ala potentially suppress spore germination in bacillus species pathogenesis.^100^ Several known antimicrobial, antifungal, and antiprotozoal agent structures are based on amino acid scaffolds, with the amino acid skeleton being essential for their activity. Daptomycin & vancomycin (glycopeptides), cycloserine, polymyxin B (lipopeptide), magainin (synthetic peptide), mersacidin (lanthipeptide), and gramicidin & cyclosporin (cyclopeptides) act on the cell membrane and function as structural analogs of various intermediates in different microbial biosynthetic pathways.^101^ The production of DAAs in bacteria is crucial for adaptation to environmental threats, as their incorporation affects bacterial growth through peptidoglycan synthesis. This, in turn, influences structural integrity and cell wall stability.

Biofilms, a microbial aggregate, often produce an extracellular matrix comprising exopolysaccharides, DNA & proteins. They are formed initially with substrate attachment, followed by monolayer to multilayer microcolony formation. Bacterial biofilms contribute to the persistence and severity of infection, making them a significant challenge.^102^ Biofilms increase bacterial antibiotic resistance and enable immune response evasion, leading to prolonged inflammation, chronic infections, and tissue damage. In medical and industrial settings, biofilms contaminate surfaces and implants, posing health risks.^100^ Therefore, to combat the severity of biofilms, several anti-biofilm approaches have been developed, such as: (i) matrix-degrading enzymes (α-amylase, DNase I, and dispersin B) to enhance antimicrobial efficacy and promote biofilm disassembly in bacteria such as *Vibrio cholerae* and *P. aeruginosa*. (ii) Disrupting quorum sensing signaling in bacteria through QS inhibitors (such as 3-benzene lactic acid (PLA), AHL-lactonases, naringenin, and oxidoreductases).^77^ (iii) Nanoparticle and antimicrobial fouling agents also offer potential solutions against biofilm production. Combining therapies of DAAs and antibiotics that target active and dormant cells is key to overcoming biofilm resistance.

**Fig. 5:**
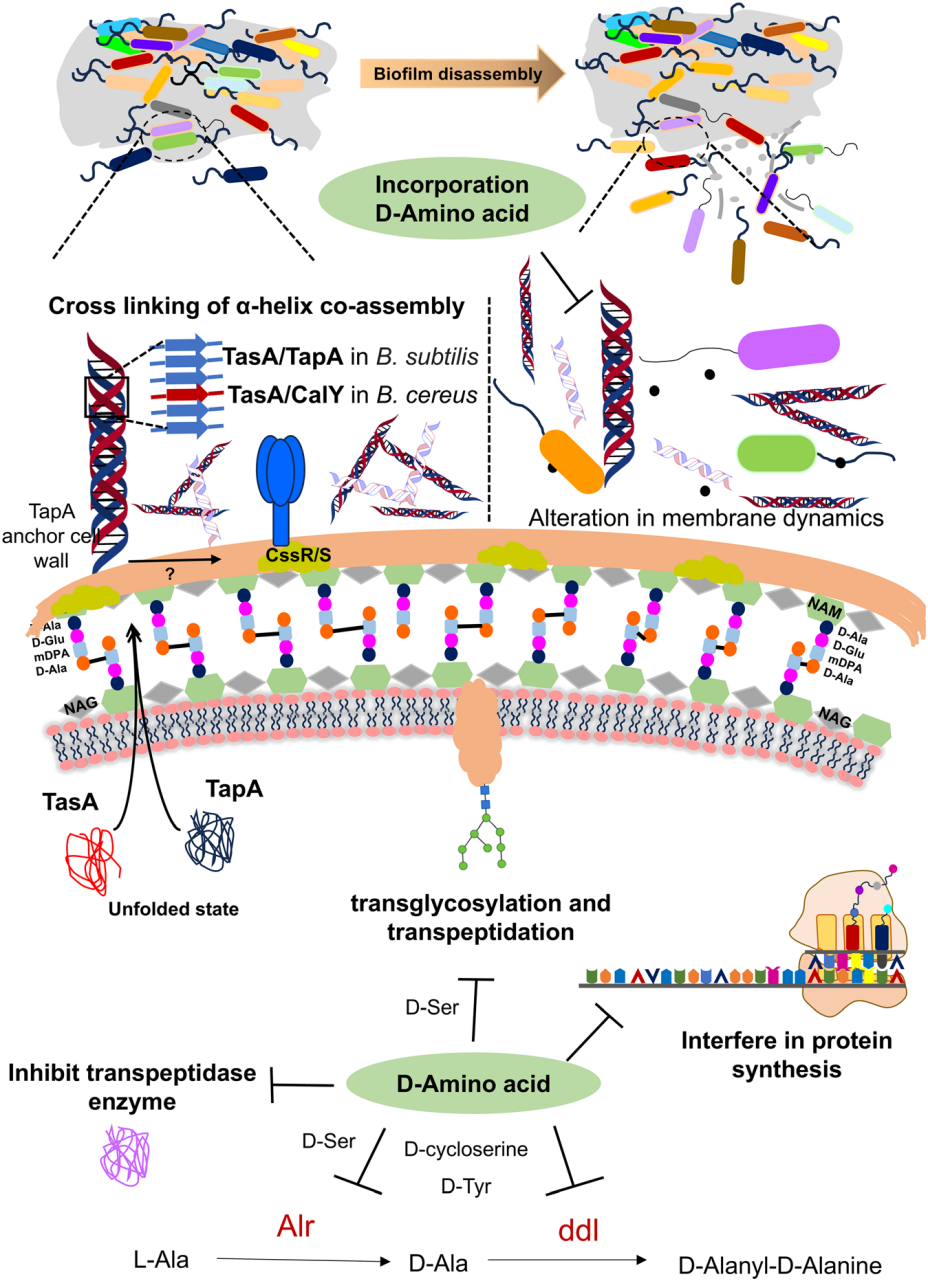
Mechanism involved in biofilm disassembly through DAAs (abbreviations: major biofilm matrix component (TasA), TasA anchoring/assembly protein (TapA), two component transcriptional regulatory protein system (CssR/S), alanine racemase (Alr), and d-alanine–d-alanine ligase (ddl)).

In recent years, DAAs have received much attention in initiating biofilm disassembly. d-Trp, d-Tyr, d-Met, and d-Leu hold the potential to initiate the disassembly of biofilms by altering multifunctional amyloids such as TapA/TasA protein in *B. subtilis* & polymerization and attachment to peptidoglycan.^103^ Mutation in TasA of *B. subtilis* leads to bacterial cell death by cell membrane destabilization, which subsequently disrupts membrane dynamics and alters matrix gene expression (Fig. 5).^100^ Later, it was found that DAAs indirectly inhibit biofilm formation in the *B. subtilis* strain by interfering with protein synthesis through the *dtd* gene, which encodes the d-tyrosyl-tRNA deacylase enzyme (*dtd*) for proofing the tRNA-amino acid bond. Mutation in the *dtd* gene leads to the misincorporation of DAAs, which disrupts protein synthesis and inhibits biofilm formation. Repairing the *dtd* gene restores enzyme function, making biofilms resistant to DAA inhibition. The inhibition of biofilm formation by DAAs is attributed to growth deficiency caused by interference with protein synthesis.^104^ In *S. aureus*, A. I. Hochbaum *et al.* demonstrated that 500 μM d-Tyr, d-Pro, or d-Phe effectively reduces the *S. aureus* SC01 biofilms. However, combining these three DAAs was effective at <100 μM. DAAs affected the protein constituent of the extracellular matrix, impairing cell wall adherence to the surface during the initial stage of biofilm formation. This disruption prevents the development and expansion of initial microcolonies into bigger assemblies.^105^

In *Staphylococcus epidermidis*, biofilm disassembly is mediated by the accumulation-associated protein (Aap), which contains a PG-binding motif and undergoes polymerization to form fibers.^106^ Here, the authors proposed that DAAs interfere with Aap's polymerization capacity, leading to biofilm disintegration. DAAs (d-Leu, d-Tyr, d-Pro, d-Phe, d-Met, and d-Ala) significantly reduce biofilm production with certain strains. However, the inhibition was also strain-dependent, thus failing to exert potent inhibition in other strains. Among all tested DAAs, d-Met was the most effective against biofilm formation.

### 6.2. Therapeutic role of DAAs as antimicrobials

Chronic wounds are a significant cause of patient morbidity and are prevalent in people with underlying medical diseases, including diabetes mellitus, as well as wounds from traumatic injuries.^107^ Chronic wounds contain several bacterial species, with *Staphylococcus* and *Pseudomonas* species being the most often isolated organisms. In wounds, bacteria primarily develop in a surface-attached biofilm state.^108^ DAAs have been demonstrated to exhibit a dispersive effect against *P. aeruginosa* and *S. aureus* biofilms. DAAs function through various mechanisms, including (i) suppressing the growth and expression of genes involved in biofilm matrix production^104^ and (ii) reducing the surface expression of fibres involved in forming biofilms due to incorporation of DAAs into the bacterial cell wall.^109^

C. J. Sanchez *et al.* evaluated the effect of DAAs against a panel of clinical isolates of *S. aureus* strains that are resistant to methicillin and found that d-Phe, d-Met, d-Trp, and d-Pro were successful at dispersing formed biofilms *in vitro*; they observed that the biofilm-dispersive activity was increased when d-Met, d-Pro, and d-Trp were mixed in an equimolar combination (0–10 wt% total; 1 : 1 : 1). The dissemination of biofilms was a unique characteristic of DAAs but did not show any effect on the growth inhibition of the bacteria. *In vitro* data and SEM images reveal that adding DAAs to polyurethane scaffolds (PUR) at a concentration of >1 wt% results in significantly reduced adherent bacteria and biofilm formation compared to PUR scaffolds without DAAs. Here, PUR scaffolds without DAAs and PUR scaffolds with 0.1% DAAs displayed significant bacterial colonization and the existence of biofilms on the surface from the bacterial count. In addition, *in vivo* studies reveal that the PUR (+DAA) implant showed a weak biofilm producer in rats segmental with 10^2^ CFU infection of *S. aureus* (Xen36 strain). However, the PUR(−DAA) implant exhibited extensive bacterial adhesion and biofilm formation.^110^

In another study, C. J. Sanchez *et al.* reported DAA activity in genetically varied clinical isolates of *S. aureus* and *P. aeruginosa* for antibiofilm activity. Individual treatment of d-Met, d-Phe, and d-Trp showed reduced biofilm production to >50%, compared to the untreated control. In contrast, combining d-Met, d-Phe, and d-Trp in an equimolar mixture (1 : 1 : 1) increased biofilm dispersal activity. The combined exposure of antimicrobial agents with DAAs is more effective against biofilm production in *S. aureus* and *P. aeruginosa*. It also reduced the observed MBIC by 4- and 8-fold for rifampicin, 6- and 4-fold for clindamycin, and 2- to 4-fold for vancomycin in *S. aureus* (methicillin-resistant and methicillin-susceptible *S. aureus*). Also, ciprofloxacin and colistin alone were ineffective against *P. aeruginosa*, but combining them with DAAs increased the effectiveness against several clinical strains. DAAs enhanced antibacterial activity, such as bactericidal levels, against *P. aeruginosa* and *S. aureus*. Thus, the biofilm dispersal activity of DAAs is an effective strategy against biofilm production and enhances the antimicrobial activity of antimicrobials.^111^

During biofilm formation, cells are held together by a self-produced extracellular matrix (ECM) consisting of proteins, exopolysaccharides, and DNA. However, certain DAAs incorporated in peptidoglycan are a natural signal to release matrix protein components. According to research by A. I. Hochbaum *et al.*, DAAs such as d-Phe, d-Pro, and d-Tyr effectively prevent biofilm formation by *S. aureus*. In contrast, LAAs were unable to inhibit biofilm formation. Fluorescence microscopy revealed that DAAs inhibit the expansion of cell foci into larger assemblies but do not prevent initial cell adhesion to the surface, thus indicating that LAAs exhibit rigid biofilm growth and DAAs prevent biofilm development.^105^

### 6.3. Nanoparticles with d-amino acids targeting antimicrobial resistance/MDR

During the cell wall synthesis, bacteria secrete various DAAs like d-Ala and d-Gln that are incorporated into peptidoglycan. Transglycosylation and transpeptidation processes are facilitated by penicillin-binding proteins (PBPs) that are crucial enzymes in the process. DAAs play a crucial role in bacterial survival and their improper incorporation can disrupt peptidoglycan synthesis causing proteotoxicity. In vancomycin-sensitive bacteria, peptidoglycan synthesis incorporates the d-alanyl–d-lactate residue in the peptidoglycan crosslink, whereas the vancomycin-resistant strain modifies peptidoglycan to d-alanyl–d-serine.^101^ Under antibiotic stress, methicillin-resistant *Staphylococcus aureus* (MRSA) can synthesize peptidoglycan with anomalous DAA residues even at a very low concentration of methicillin (0.3%).

Bacteria produce various d-stereospecific peptidases that hydrolyze peptides with DAAs through stereospecific cleavage. These d-stereospecific peptidases act as a bacterial defense mechanism, providing self-resistance against non-ribosomal peptide antibiotics such as polymyxin, vancomycin, and teixobactin.^112^

A major global public health issue in the twenty-first century is the rise of antimicrobial resistance, leading to an increase in multidrug-resistant (MDR) infections. Several antibiotics have been used to treat bacterial infections, but their misuse leads to MDR.^113^ Antimicrobial peptides (AMPs) are a class of small peptides involved in the innate immune system that have garnered attention as potential alternative antibiotics. Most AMPs are cationic and typically comprise 10–60 amino acid residues, whereas acidic amino acids (especially Asp and Glu) are found in different anionic AMPs.^114^ DAA peptides interact with enzymes or receptors and resist proteolytic cleavage by self-associating within the bacterial membrane rather than entering the cytosol. J. H. Shim *et al.* synthesized various d-amino acid-based surfactant (DAAS) gold and silica nanoparticles to increase the efficiency of antibacterial agents. Among them, *N*-α-lauroyl-d-arginine ethyl ester hydrochloride (d-LAE), d-Pro dodecyl ester (d-PD) and d-Ala dodecyl ester (d-AD) exhibited antibacterial activity against Gram-(+) and Gram-(−) bacteria but showed reduced efficacy against Gram-(−) bacteria. d-LAE is more effective against Gram-(−) bacteria than d-PD and d-AD. The d-LAE antibacterial activity was unaffected by AuNP or SiNP coating. However, the d-PD and d-AD efficacies were improved when coated with AuNPs. The antibacterial activity of DAAs and their nanocomposites with AuNPs and SiNPs was strongly impacted by amino acid chirality, emphasizing their potential as effective agents against multidrug-resistant infections.^115^

DAAs have an opposite physiological effect on the pace of bacterial growth from suitable solutes with a comparable structure. Trp-rich peptides are a significant subset as they exhibit a high level of membrane-disruptive activity, allowing Trp-containing antimicrobial drugs to interact with the surface of microbial cell membranes. Additionally, Trp itself exhibits broad and robust antibacterial activity.^116^ Several strategies, including antimicrobial photodynamic therapy (aPDT) and antimicrobial sonodynamic therapy (aSDT), can be employed to increase the effectiveness of AMPs against microbes. Hypericin nanoparticles (HypNPs) are photo-sonosensitizers that are excited upon exposure to ultrasonic or visible light waves. HypNP and d-Trp combination as HypNP@d-Trp shows synergistic activity against *A. baumannii*, enhancing the antibacterial efficacy of a photo-sonodynamic therapy (PSDT). The cell viability decreased by 5.10 log_10_ CFU mL^−1^ after treatment with HypNP@d-Trp, and significant biofilm degradation was observed in HypNP@d-Trp compared to controls. Molecular docking studies found that Hyp had a high binding affinity for AbaI (−9.41 kcal mol^−1^), and a PSDT treatment downregulated the *AbaI* gene expression by 10.32-fold. Thus, the Trp combination with HypNPs is a promising strategy to reduce the growth of biofilm production during pathogenic infection by *A. baumannii*.^117^

DAAs signal to disassemble biofilms by altering metabolic pathways, which provides an opportunity to improve antibiotic efficacy. The W. Feng *et al.* group developed 3D poly(α-*N*-acryloyl-d-phenylalanine)-*block*-poly(β-*N*-acryloyl-d-aminoalanine NPs (FA NPs) to inhibit intracellular metabolism and destabilize proteins in extracellular polymeric substances. α-Amino and α-carboxyl groups of d-Ala present on their surface ensure that FA NPs were efficiently inserted into bacterial peptidoglycan (PG) through the assistance of PG binding protein 4 (PBP4). Thus, FA NPs composed of d-Ala motif enhance the penetration and efficacy of encapsulated sitafloxacin sesquihydrate, achieving complete eradication of staphylococcal biofilms in mice and offering great potential for bacterial biofilm infection treatment.^118^

## 7. Role of l-amino acids in therapeutic design

### 7.1. Amino acid conjugates as antibacterial and antifungal agents

**Scheme 1:**
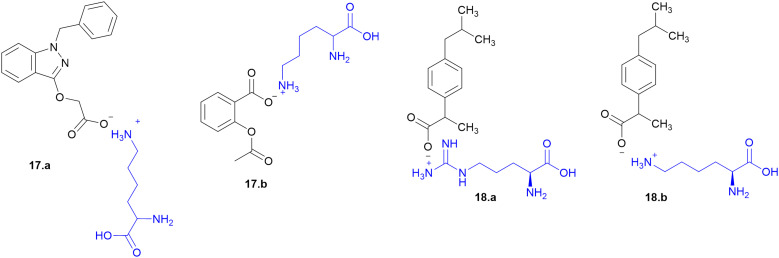
Amino acid containing pharmaceutical drug co-crystals.

Many APIs exhibit poor solubility issues, which lead to poor bioavailability and therapeutic efficacy. To overcome these limitations, APIs combine with co-formers to form co-crystals and hydrates. In the search for greener and safer options, amino acids represent attractive co-formers due to their safety, natural abundance, non-toxicity and high water-solubility, facilitating greener methods for co-crystallization processes. Incorporation of amino acids into co-crystals has been shown to improve solubility, permeability and reduced adverse effects. Based on these properties, various pharmaceutical co-crystals have been developed such as bendazac Lys (17a), Lys acetylsalicylate (17b) and ibuprofen Arg (18a) or Lys (18b) (Scheme 1).

**Scheme 2:**
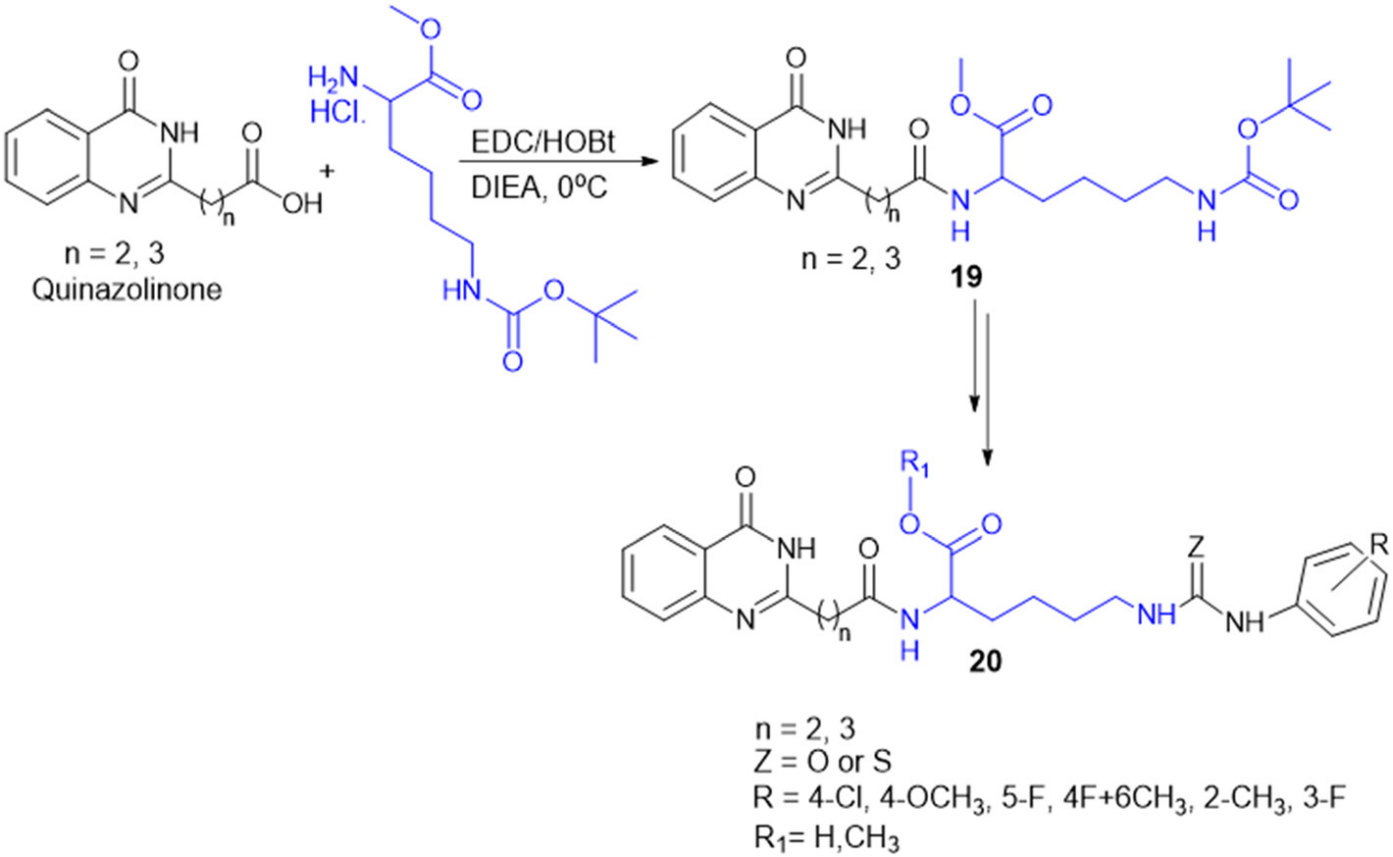
Synthetic method for quinazolinone–Lys conjugates.

Considering the benefits of amino acids, the authors synthesized a series of quinazoline-l-Lys (19) conjugates (Scheme 2). Further, the ε-amino group of Lys-modified substituents is screened for antibacterial activity against Gram-(+) (*B. subtilis*) and Gram-(−) (*E. coli*, *P. fluorescens*, and *X. campestris*) strains. According to the activity profile, compounds containing fluoro group, urea, and thiourea (20) groups exhibited antibacterial activity. The activity drastically decreased when the ε-amino group of Lys was acetylated and sulphonated.^88^ Quinazolinones were linked to Lys for the conjugation reaction using DIEA as a base and EDCl/HOBt as a coupling agent. The ε-amino group of Lys was substituted to urea, thiourea, acetamide, and methyl sulphonamide derivatives from isocyanates, isothiocyanates, acetyl chloride, and methyl sulphonyl chloride, respectively.^119^

**Scheme 3:**
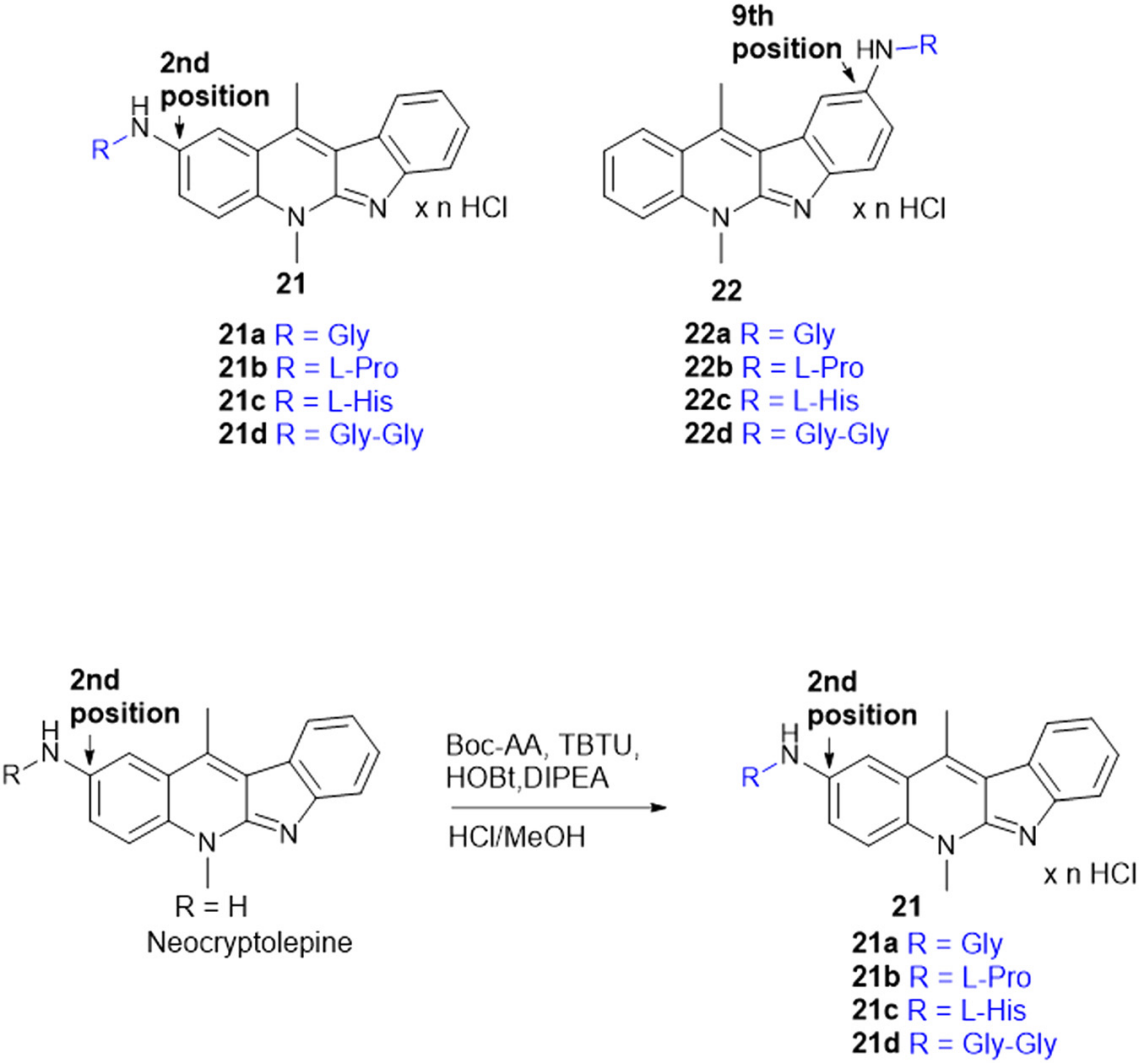
Synthesis of neocryptolepine–amino acid conjugates.

Indoquinoline alkaloids, mainly comprising quinoline and indole moieties, are the active ingredients of an African plant species *Cryptolepis sanguinolenta*. Neocryptolepine (5-methyl-5*H*-indolo[2,3-*b*]quinoline) is a minor alkaloid of *C. sanguinolenta*, although the principal alkaloid of the roots, cryptolepine (5-methyl-5*H*-indolo[3,2-*b*]quinoline), is claimed to have complex biological effects. K. Sidoryk *et al.* synthesized a set of neocryptolepine derivatives substituted with an amino acid or a dipeptide at the C-2 or C-9 position evaluated for antiproliferative and antimicrobial/antifungal activities. The synthesized compounds were screened for antiproliferative activity in cancer cell lines (KB, A549, MCF-7, and LoVo) and normal mice fibroblast cells (BALB/3T3). Gly substitution at C-2 position (21a) (Scheme 3) showed the most potent antiproliferative activity against all cell lines. Also, all the synthesized molecules were tested for antibacterial and antifungal properties. The synthesized molecules were active against Gram-(+) bacteria and *Candida* species but inactive against Gram-(−) bacteria. Neocryptolepine conjugates of l-Gly and l-Pro substitution at C-2 and C-9 positions (21a, 21b, 22a, and 22b) showed antibacterial properties against Gram-(+) bacteria. Meanwhile, 21a, 22a, and 22b showed the highest activity against the *in vitro* fungal biofilm model.^120^

**Scheme 4:**
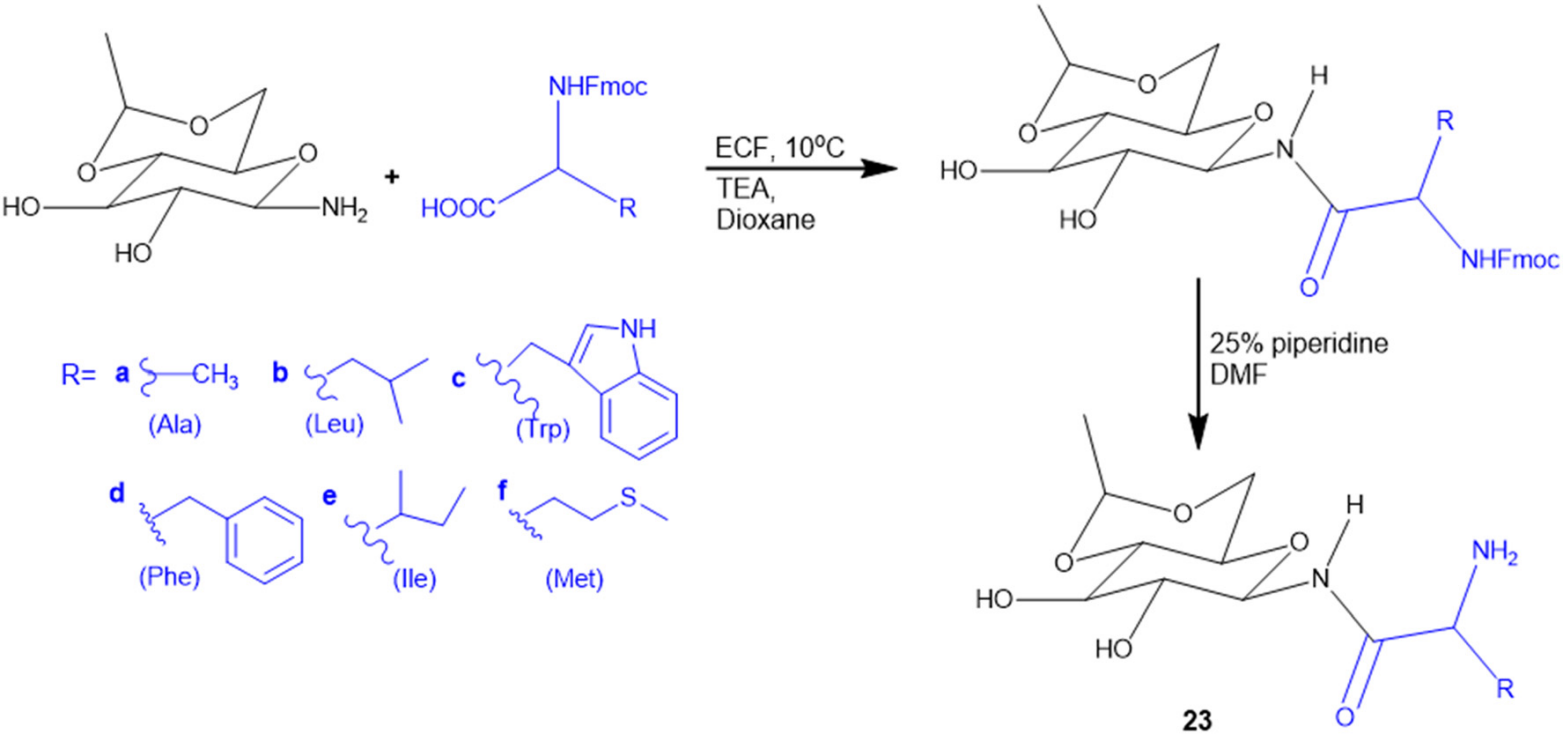
Synthesis of glycol-conjugated amino acid.

Different amino acids (Ala, Val, Trp, Phe, Ile, and Met) were conjugated with carbohydrates to obtain a series of amino acid-derived *N*-glycoconjugates of d-glucose-containing molecules (23) (Scheme 4). The synthesized molecules were screened against Gram-(+) (*B. cereus*) and Gram-(−) (*E. coli and K. pneumoniae*) bacterial strains. Among conjugates of compound 23, in comparison with the standard drug chloramphenicol, 23c (Trp conjugate) showed the highest potency against *E. coli* and *B. cereus*. Meanwhile, 23e (Ile conjugate) showed the highest antibacterial activity against *K. pneumoniae*; all of the compounds' minimum inhibitory concentrations (MICs) were in 16–32 μg mL^−1^ range. The structural similarities among the synthesized molecules with quinolone antimicrobial drugs like clorobiocin and novobiocin act by inhibiting bacterial type II topoisomerase DNA gyrase, composed of GyrA and GyrB subunits. Clorobiocin and novobiocin exert antibacterial activity by inhibiting the GyrB-associated ATPase through their chiral oxygen-bridged six-membered ring system. In contrast, the synthesized amino acid-glycoconjugates in this study contain structural feature more closely related to quinolone-based antimicrobials. Among all the amino acid conjugates docked with GyrB (PDB: 3TTZ), the docking study revealed that compounds 23c and 23e showed the highest glide scores, −9.15 and −8.03, respectively. The Trp moiety formed key hydrophobic interactions with the GyrB binding pocket, while the indole phenyl ring contributed additional hydrophobic contacts. In contrast, the amine group and the amide –NH– engaged in hydrogen bond interactions, supporting ligand stabilization within the active site.^121^

**Scheme 5:**
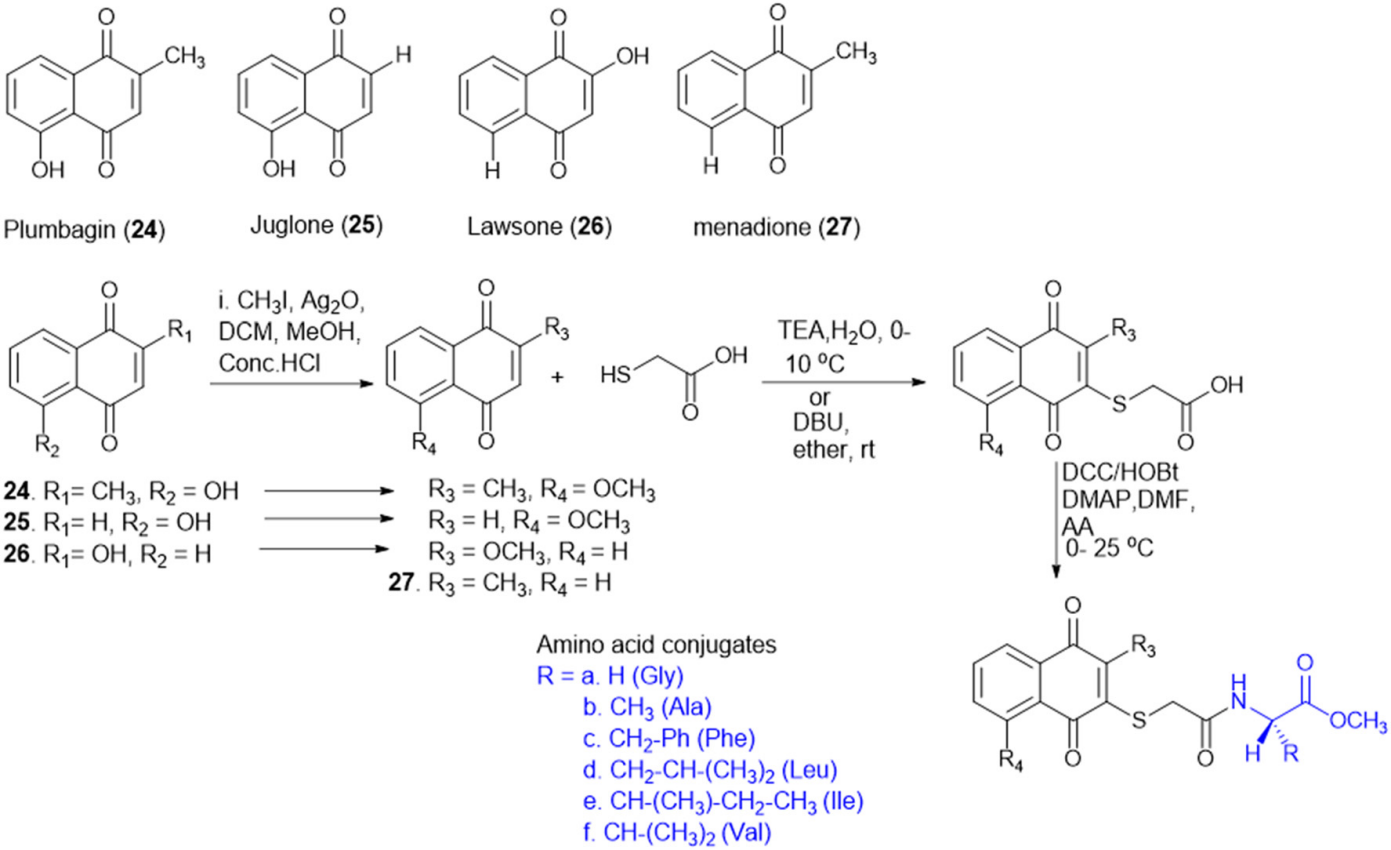
Synthesis of naphthoquinone–amino acid conjugates.

T. Sreelatha *et al.* synthesized a series of naphthoquinone amide derivatives containing different amino acids, based on bioactive quinones such as plumbagin, juglone, menadione, and lawsone (24–27; Scheme 5). Natural anticancer agents were conjugated to the thiol group and neutral amino acids, including Gly, Ala, Phe, Ile, Leu, Val, and γ-aminobutyric acid (GABA). The anticancer activity of all the compounds was assessed against HeLa (cervical cancer) and SAS (oral cancer) cancer cell lines. The cytotoxic effects of all the parent quinones (plumbagin, juglone, menadione, and lawsone) and their derivatives were more sensitive to SAS cells than to HeLa cells, indicating that the cytotoxicity may vary amongst cancer cells of different tissue origins. Additionally, the compounds 24–27 were tested for antifungal and antibacterial effectiveness against different human pathogens: fluconazole-resistant *C. albicans* (FRCA), Gram-(+) methicillin-resistant *S. aureus* (MRSA), and Gram-(−) *P. aeruginosa*. GABA amide conjugates showed the most potent antibacterial properties. Compared to the parent quinones, the amide derivatives demonstrated better antibacterial efficacy against MRSA and FRCA with a zone of inhibition (ZOI) of up to 26 mm. The molecule 25a, the Gly amide of juglone, had noteworthy antibacterial and anti-yeast properties with MIC values of 3.9 and 7.8 μg mL^−1^, lower than the MIC of juglone, the parent drug. Moreover, compound 26d, lawsone's Leu amide, had strong antibacterial and anti-yeast properties at an MIC of 7.8 μg mL^−1^, less than lawsone, the parent molecule. The Ile amide of plumbagin (27e) showed good antibacterial and anti-yeast activity at an MIC of 7.8 μg mL^−1^ compared to its parent chemical plumbagin with MIC = 125 μg mL^−1^.^122^

**Scheme 6:**
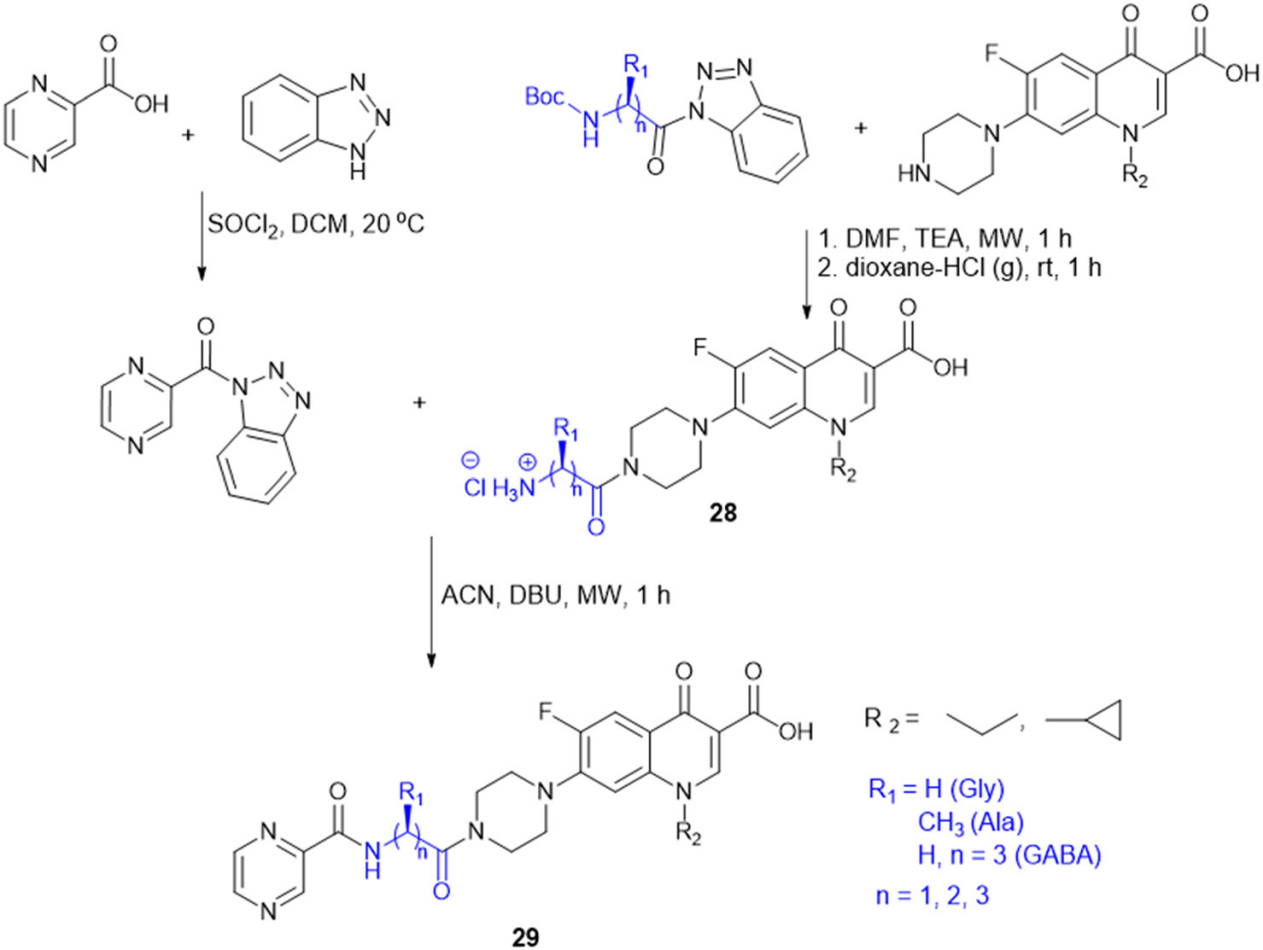
Synthesis of fluoroquinolone–pyrazine amino acid conjugates.

Amino acids have been reported as drug carriers and are used in the prodrug design of pyrazinamide molecules to enhance their antimicrobial properties against Gram-(+) (*S. aureus* and *S. pyogenes*) and Gram-(−) (*S. typhii*) bacteria: novel fluoroquinolone–pyrazine (28) conjugated with amino acids Gly, Ala, β-Ala, and GABA. The fluoroquinolone–pyrazine was prepared by coupling conjugate 28 with (1*H*-benzo[*d*][1,2,3]triazol-1-yl)(pyrazin-2-yl)methanone in the presence of DBU followed by reaction conditions mentioned in Scheme 6. Conjugate 29 (*n* = 3, GABA) showed enhanced antibacterial activity against Gram-(+) and Gram-(−) bacteria. Thus, the compound containing GABA as an amino acid linker seems a better choice for enhancing antibacterial activity.^123^

**Scheme 7:**
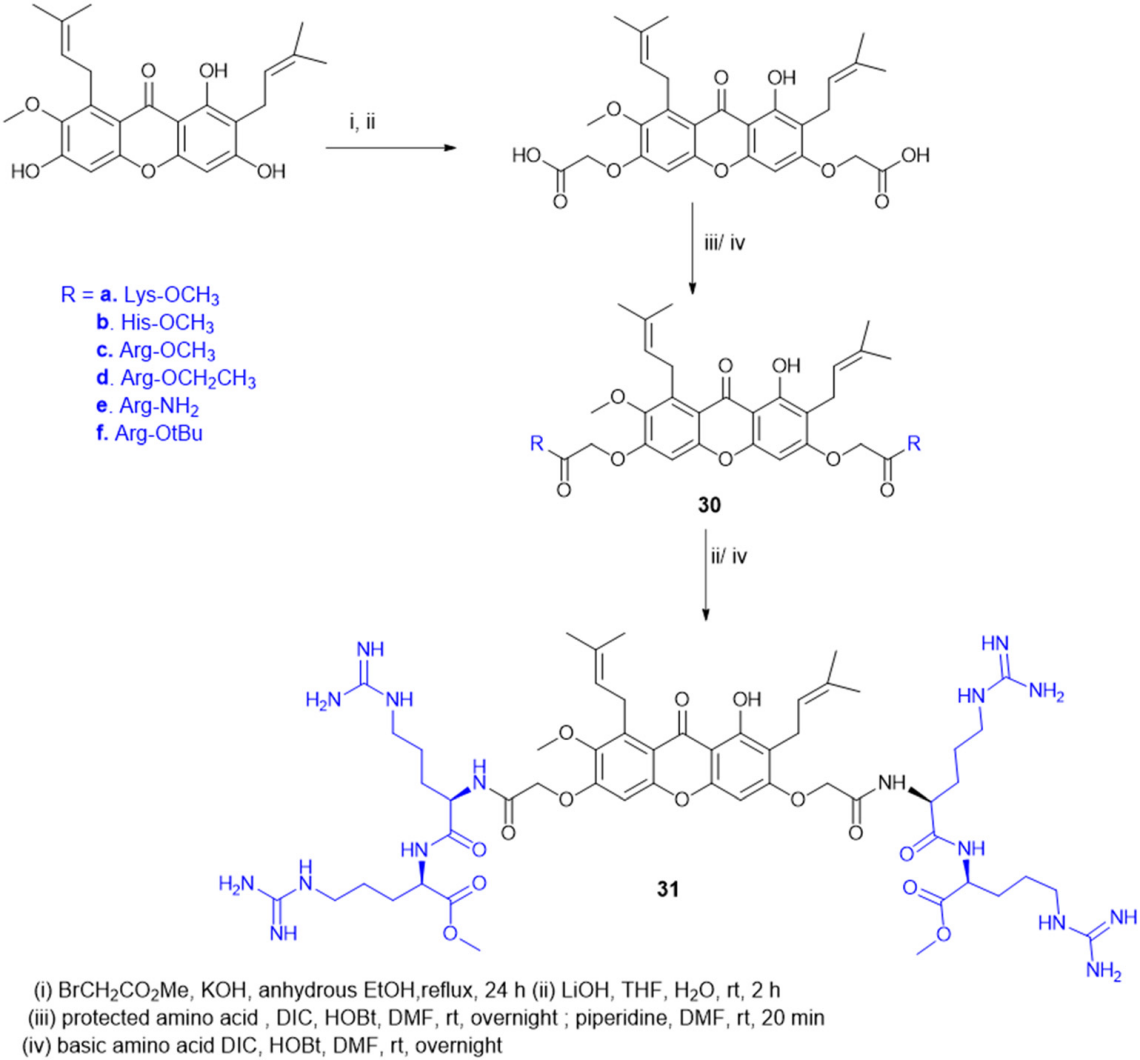
Synthesis of xanthone analogs (amino acid conjugates).

Antimicrobial cationic peptides were designed to produce membrane-targeting antimicrobials, where the xanthone core (α-mangostin) is used as a hydrophobic scaffold. Cationic moieties containing amino acids increase the membrane selectivity to form an amphiphilic structure and separate the bacterial membrane from the mammalian membrane. For example, cationic amino acids like Arg, His, and Lys have been conjugated to α- mangostin 30(a–f) (Scheme 7), and all the molecules were screened against MRSA and VRE strains. Among the synthesized molecules (30), the Arg conjugate (30c) showed good antimicrobial activity as compared to the Lys-containing molecule (30a) against multidrug-resistant Gram-(+) bacteria. Lys has a single ε-amino group, but the guanidinium group of Arg has a more scattered positive charge. The cationic moiety is necessary to create an amphiphilic structure to distinguish between the bacterial and mammalian membranes. Compound 31 shows antibacterial properties with MIC = 0.5–3 μg mL^−1^, whereas α-mangostin showed MIC = 2 μg mL^−1^. The selectivity of 30c and 31 was tested against membrane-active drugs in clinical trials (LTX-109, PMX-30063, and CSA-13), and they showed similar or better activity. Similarly, 30c and 31 treatments, in an *in vivo* wound healing model of corneal infection, showed reduced inflammatory signs and promoted wound closure in molecule 31. Arg's cationic properties with dispersed positive charges show immense therapeutic templates against antimicrobial activity.^124^

**Scheme 8:**
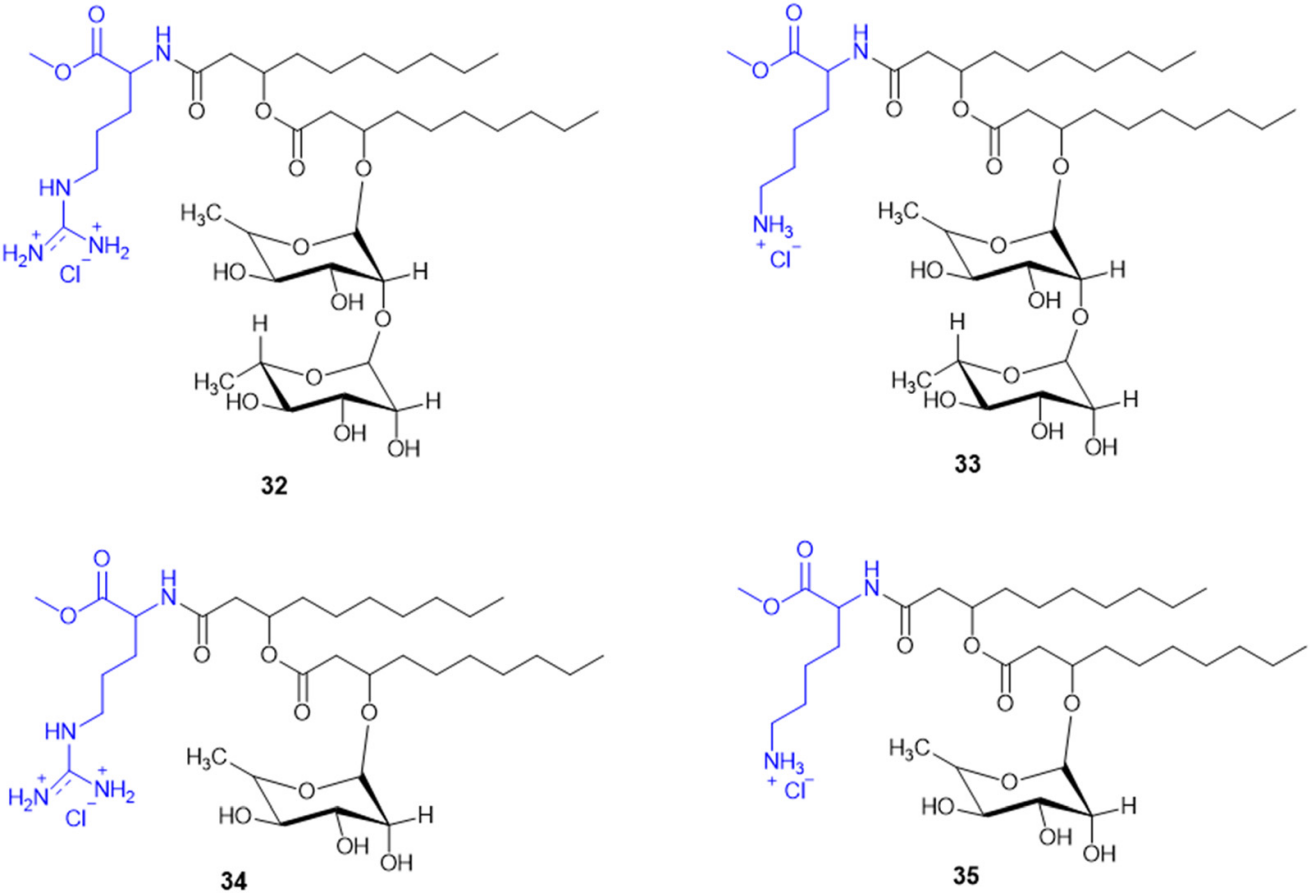
Synthesis of mono and di-rhamnolipid amino acid conjugates.

Rhamnolipids (RLs) from the *Pseudomonas* species are environmentally friendly surfactants, which when functionalized with cationic moieties show increased multifunctionality such as antimicrobial and drug and gene delivery properties, with sustainable production using low-cost substrates. New cationic Arg–rhamnolipid (RL) derivatives (Scheme 8) were prepared in one step by using H-Arg-OMe (32 and 34) and RLs and DCC/HOBt as coupling reagents. Lys-derived derivatives (33 and 35) needed a two-step approach of Cbz-Lys-OMe conjugation followed by hydrogenation-induced Cbz deprotection. The Arg-conjugated RLs showed the most effective antibacterial activity against Gram-(+) bacteria (*B. subtilis*, *S. epidermidis*, *S. auereus*, and *L. monocytogeneses*) with MIC values ranging from 4 to 32 μg mL^−1^. This improved activity can be ascribed to the cationic nature of Arg, facilitating stronger electrostatic forces with the negatively charged bacterial membrane. On the other hand, the cationic-RL derivatives were not active (>250 μg mL^−1^) against any of the Gram-(−) bacteria (*P. aeruginosa*, *K. pneumoniae*, and *E. coli*) because of their single outer membrane charged with lipopolysaccharides that acts as an effective permeability barrier. Biodegradation assays confirmed that these conjugates are environmentally sustainable, validating their application as biocompatible, multifunctional antibacterial agents for pharmaceutical or industrial purposes.^125^

A. Da Silva *et al.* reported the synthesis and biological assessment of Arg-derived cationic rhamnolipids (RLs) as multistep antimicrobial agents. The new RL conjugates (32–34, Scheme 8) displayed strong antifungal activity against fluconazole-resistant *Candida krusei* and non-resistant *C. albicans*, *C. parapsilosis*, and *C. tropicalis* with MIC values from 6.5 to 20.7 mg L^−1^. Besides, the compounds also displayed strong anti-biofilm activity by effectively destroying matured *Candida* sp. biofilms at concentrations higher than MIC values. Arg derivatization in RL conjugates improves antibiofilm activity by leveraging its cationic charge to facilitate biofilm penetration. Importantly, these RLs displayed complete amoebicidal activity against *Acanthamoeba castellanii* trophozoites at concentrations as low as 4 mg L^−1^. Mechanistic investigations revealed that the conjugates disrupt cellular membrane integrity, facilitating increased permeability and inducing apoptotic-like cell death. Cytotoxicity assessments conducted in the human keratinocyte (HaCaT) and human fibroblast (Hs27) cell lines reveal minimal cytotoxicity and the selectivity of the antimicrobial efficacy of these RLs (SI > 10). Taken together, the work offers conclusive evidence for amino acid-modified rhamnolipids, specifically with arginine-containing moieties, being an appealing class of biocompatible substances to fortify their therapeutic potency against multi-resistant fungal pathogens as well as protozoan parasites.^126^

**Scheme 9:**
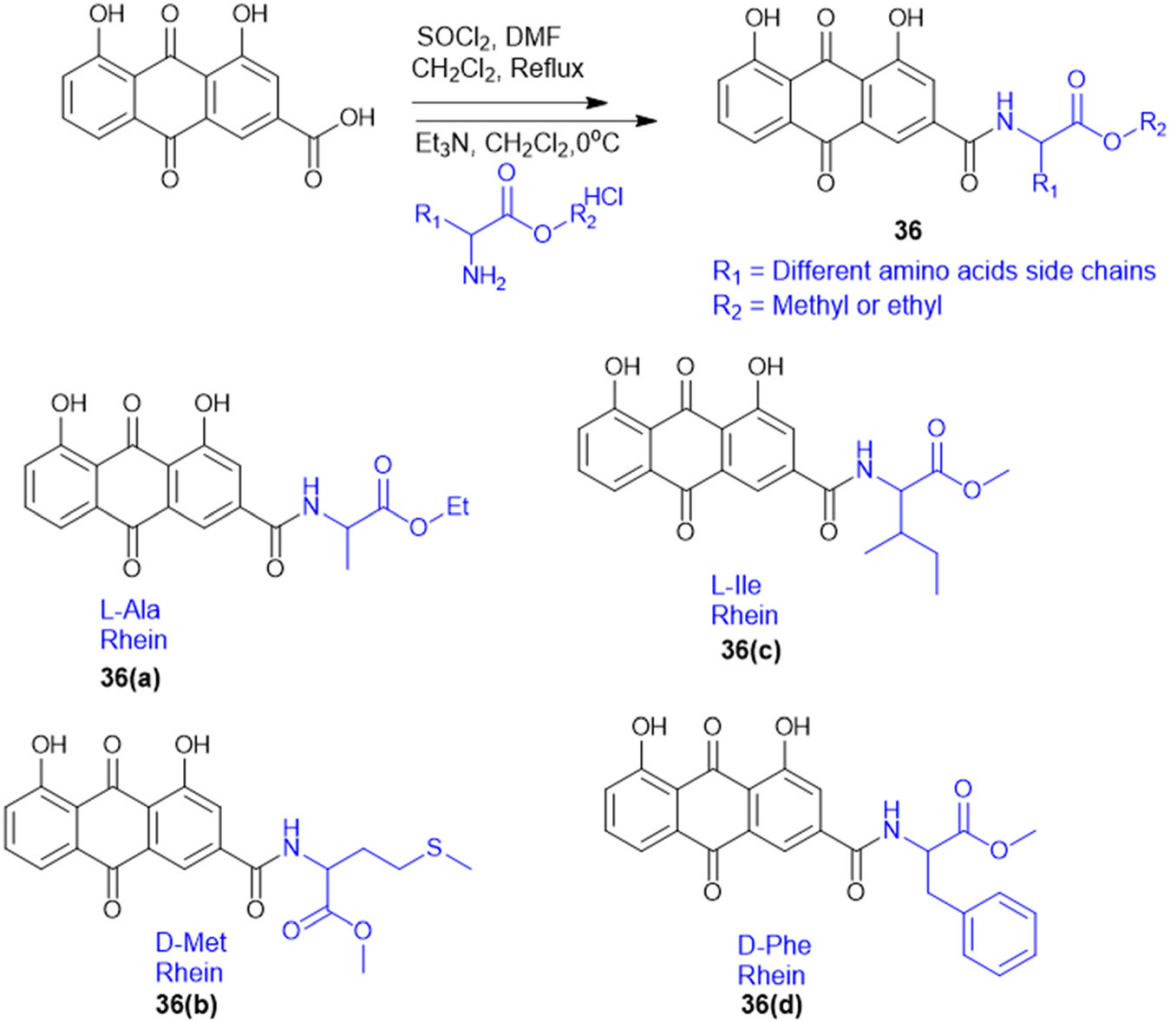
Synthesis of rhein amino acid conjugates.

S. Chen *et al.* prepared rhein–amino acid ester conjugates (36, Scheme 9) to maximize the biological activity of rhein by altering its pharmacological and structural features for antifungal activity against four phytopathogenic fungi (*Rhizoctonia solani*, *Sclerotinia sclerotiorum, Bipolaris maydis*, and *Phytophthora capsici*). Synthesis was done by esterification of rhein's carboxylic group with different l- and d-amino acid methyl or ethyl esters using EDC·HCl and DMAP as the coupling agents. The resulting compounds were tested for antifungal activity against *R. solani* and *S. sclerotiorum*. Among them, the rhein–l-Ala ethyl ester conjugate (36a) demonstrated the highest potency against *R. solani*, with an EC_50_ value of 0.125 mM, while the rhein–Met methyl ester conjugate (36b) showed the strongest activity against *S. sclerotiorum*, with an EC_50_ of 0.114 mM. Conjugates with l-Ile (36c) and d-Phe (36d) also displayed notable antifungal effects. Also, 36a showed better curative and protective effect against wheat powdery mildew. These results indicate that amino acid esterification significantly enhances the antifungal activity of rhein, likely due to improved lipophilicity, cell membrane permeability, and favourable interactions with fungal targets.^127^

**Scheme 10:**
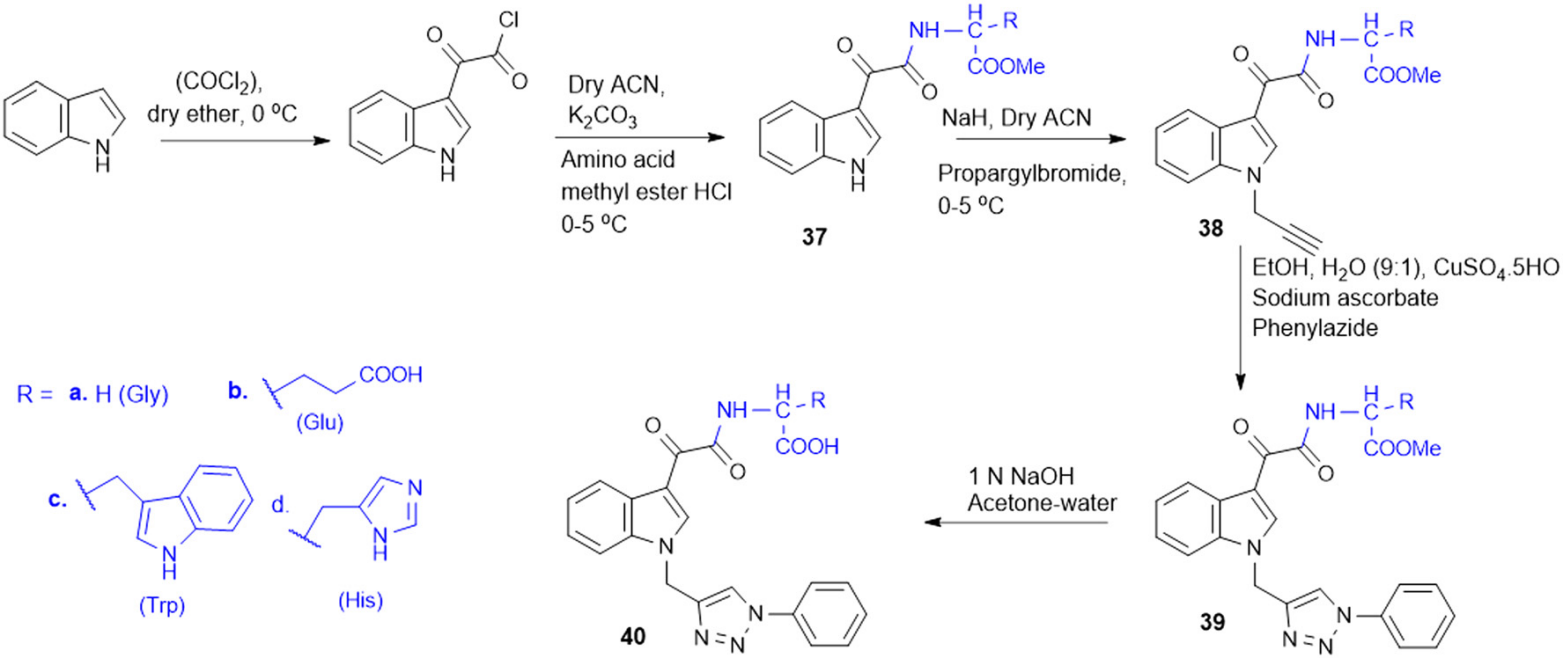
Synthesis of indole–triazole amino acid conjugates.

The indole–triazole–amino acid conjugates (37–40, Scheme 10), synthesized by Pawar K. and colleagues, represent promising antifungal agents designed through a strategic multi-step synthetic approach. The synthesis of these conjugates involves initial acylation of indole followed by coupling with an l-amino acid ester to form a 3-substituted indole which is further modified *via* propargylation and a Cu-catalyzed click reaction with phenyl azide to yield the final triazole-linked conjugate. Among them, four terminal conjugate molecules (Gly, l-Trp, l-His, and l-Glu-conjugated) were found to have strong antifungal activity against *C. albicans* with a high MIC-80 value in liquid culture of 312.5 μg mL^−1^. Furthermore, in synergy with ketoconazole, the Glu-triazole conjugate (40b) demonstrated a synergistic effect with a remarkable change in the chitin level, cell morphology, and fungal viability. The compound also caused significant cell wall stress, adding to morphological alteration and elevated rates of necrosis, further substantiating its promise as a powerful antifungal candidate by affecting several pathways in fungal cells.^128^

**Scheme 11:**
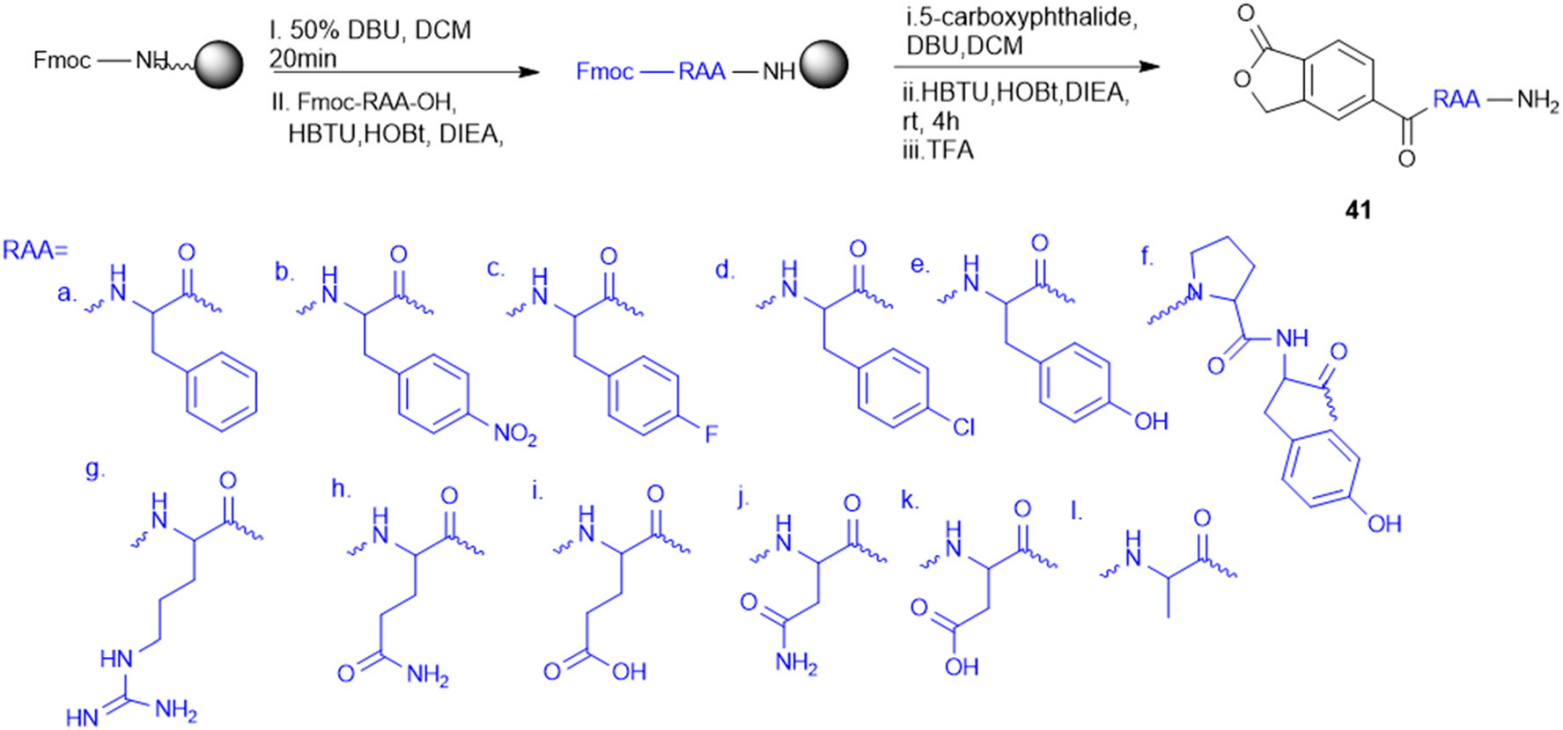
Synthesis of phthalide–amino acid conjugates.

**Scheme 12:**
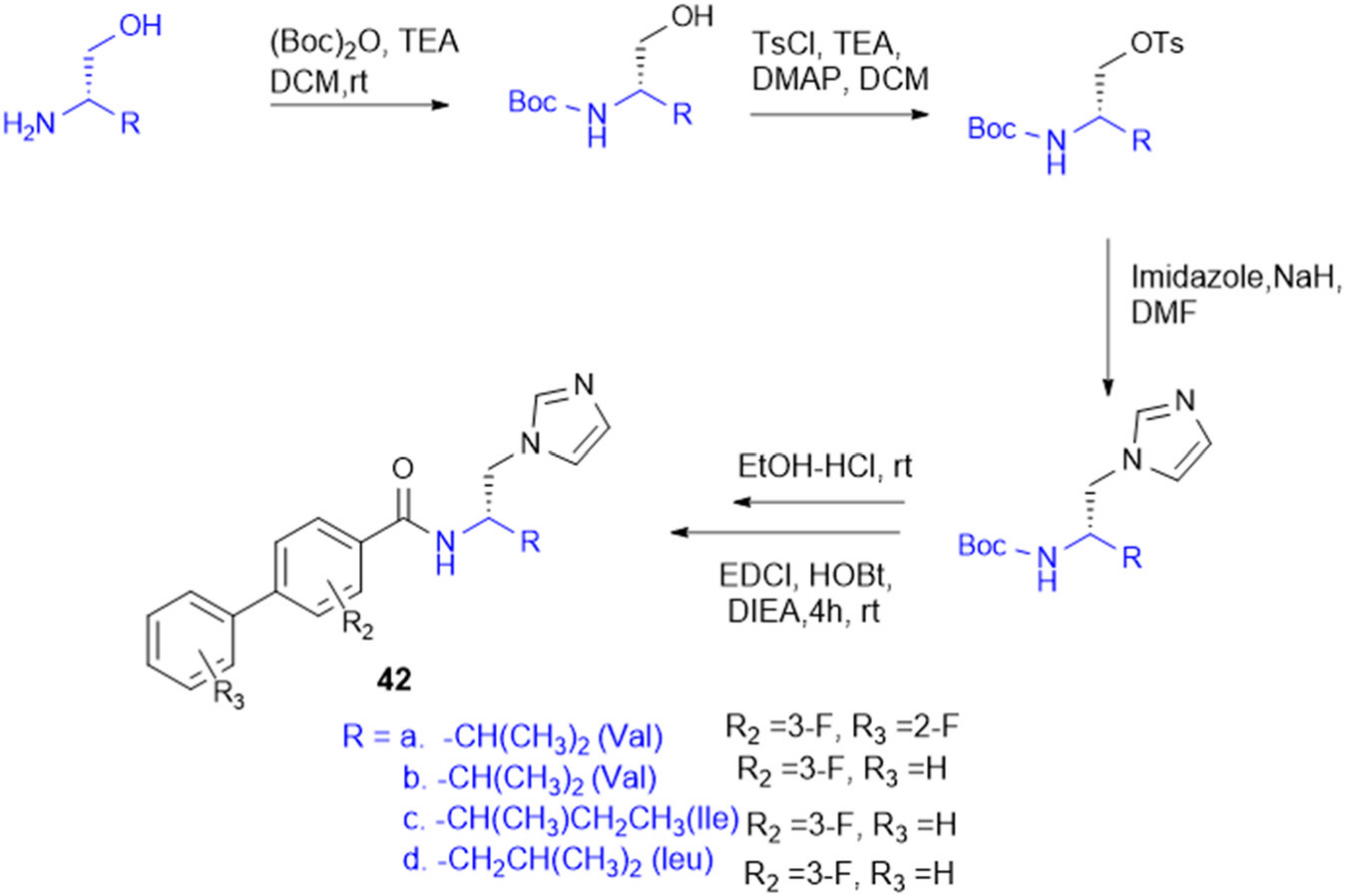
Synthesis of l-amino alcohol conjugates.

Amino acid-targeting phthalide derivatives (41, Scheme 11) were synthesized and screened for bioactivity against peanut southern blight, a fungal disease infection caused by *Sclerotium rolfsii*. Classical fungicides including succinate dehydrogenase inhibitors such as thifluzamide, flutolanil, and boscalid have shown toxicity to non-target organisms and an increased risk of resistance, necessitating the development of eco-friendly alternatives. To address this challenge, M. Wang *et al.* synthesized fourteen new phthalide compounds with amino acid moieties, where the Phe–phthalide conjugate (41) exhibited promising antifungal activity. *In vitro* results showed that 41a had an EC_50_ value of 332.21 mg L^−1^, slightly lower than polyoxin (EC_50_ = 284.32 mg L^−1^), yet it's *in vivo* curative efficacy was better, with a recovery rate of 57.75% at 600 mg L^−1^ compared to the 42.55% at 300 mg L^−1^ of polyoxin. The Phe–phthalide conjugated (41a) molecule displayed dramatic agronomic enhancements, confirming its efficacy by improved uptake and transport compared to traditional fungicides, which helps to better control diseases. Cytotoxicity tests confirmed its low cytotoxicity to human liver cells, further substantiating its utility as a green fungicide and opening up a new avenue for sustainable plant protection to be further developed for use in agriculture.^129^

l-Amino alcohol derivatives, broad-spectrum antifungal agents, were synthesised, aiming to address the fungal infection in immunocompromised patients. Zhao L. and colleagues employed two strategic approaches to modify existing azole antifungals, resulting in a novel class of l-amino alcohol derivatives using EDC/HOBt as a coupling agent. Among the synthesised molecules, compound 42 (Scheme 12) with 3-F substituted derivatives – specifically an alkyl group containing amino acids (Val, Leu, and Ile) – exhibits potent antifungal activity against *C. albicans*, *C. tropicalis*, *A. fumigatus*, and *C*. *neoformans*, with moderate effectiveness against fluconazole-resistant strains isolated from AIDS patients. Meanwhile, SAR analysis emphasizes that fluorine substitution increases antifungal activity by halogen and hydrogen bonding interactions in the CYP51 active site, inhibiting ergosterol biosynthesis and causing sterol composition modification like fluconazole. These results indicate that l-amino alcohol derivatives, especially the Leu-conjugated compound, are good candidates for further consideration as broad-spectrum, low-toxicity antifungal drugs.^130^

### 7.2. Amino acid conjugates as antitubercular agents

The cell wall of mycobacteria and related genera exhibits a distinct composition compared to that of Gram-(+) and Gram-(−) bacteria. It contains a high lipid content, contributing to a hydrophobic nature. Arabinogalactan (AG) and peptidoglycan (PG) are covalently linked, forming a robust backbone. Most of the mycobacterial cell wall consists of exceptionally long, high molecular weight fatty acids known as mycolic acids, which are esterified at the termini of AG molecules. The hydrophobic mycolic acid envelop of bacilli creates a protective barrier that guards against complement deposition, oxidative damage, and hydrophilic antibiotics.^131^

**Scheme 13:**
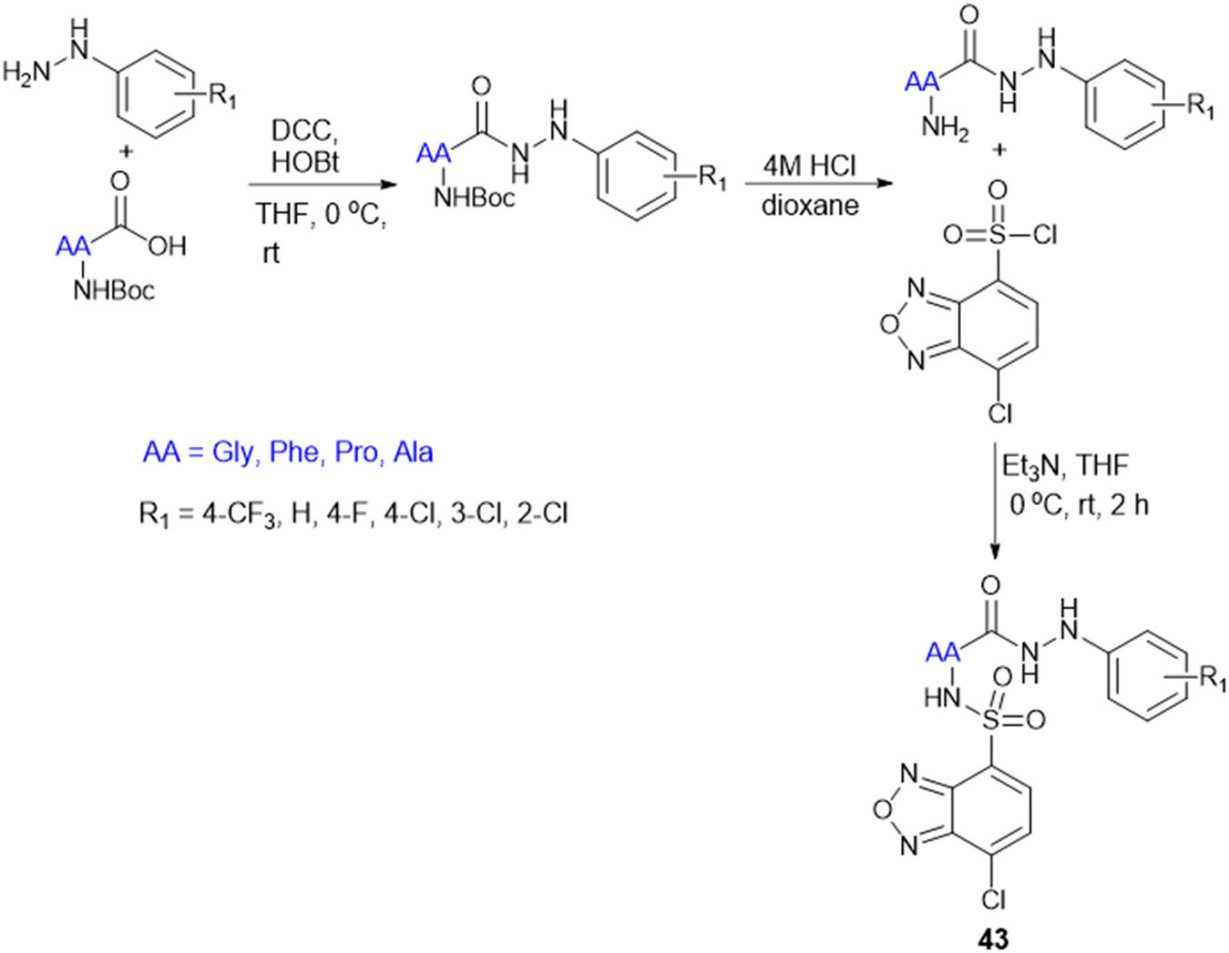
Synthesis of benzoxa-[2,1,3]-diazole–amino acid conjugates.

To address the ongoing worldwide burden of tuberculosis (TB) and the inadequacies of cost constraints in existing treatments, novel therapeutic options for the treatment of Mtb infection need to be developed. Amino acid conjugation with heterocyclic moieties helps to increase selectivity, potency, and efficacy and avoid toxicity. A series of benzoxa-[2,1,3]-diazole substituted amino acid hydrazides (43) were synthesized using *N*-Boc-amino acids (Gly, Phe, Pro, and Ala), and DCC and HOBt as coupling reagents (Scheme 13). Among these, the unsubstituted hydrazide containing Gly amino acid shows good antitubercular activity and is less toxic, indicating better selectivity for Mtb.^132^

**Scheme 14:**
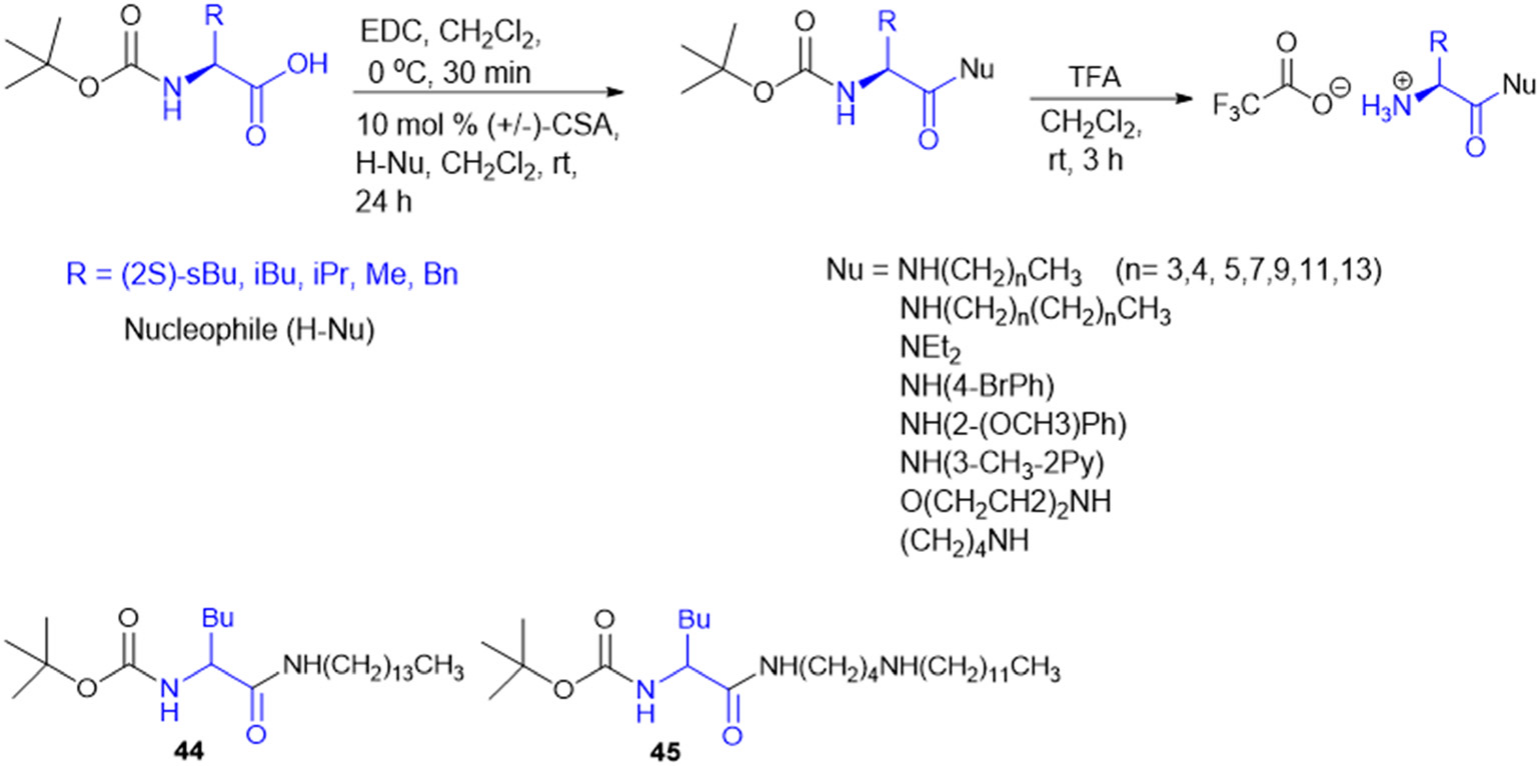
Synthesis of dual-protected amino acid derivatives.

For the design of drugs targeting the bacterial cell wall, DAAs or their analogues are preferred as the peptidoglycan layer of the cell wall predominately consists of DAAs. LAAs are employed in drugs designed to withstand bacterial enzyme metabolism and enhance activity. P. P. de Castro *et al.* synthesized a carbamate scaffold with LAA-conjugated compounds (l-Ala, l-Val, l-Leu, and l-Phe) against tuberculosis (Scheme 14). Various amines can functionalize carboxyl groups of amino acids to enhance stability, particularly compared to ester counterparts susceptible to enzymatic degradation. This functionalization allows for precise control over the lipophilicity of these molecules. Increased lipophilicity facilitates penetration into the mycobacterial membranes; therefore, lipophilicity is crucial in modifying the physicochemical properties that enhance pharmacokinetics. From all the synthesized molecules screened for antitubercular properties, compounds 44 and 45 (Scheme 14) had inhibitory activity (MIC_90_) below 40 μM. However, the most intriguing finding was the clear association between an increase in log *P* and an improvement in antitubercular activity, with most lipophilic substances (log *P* > 6) showing the best inhibition.^133^

**Scheme 15:**
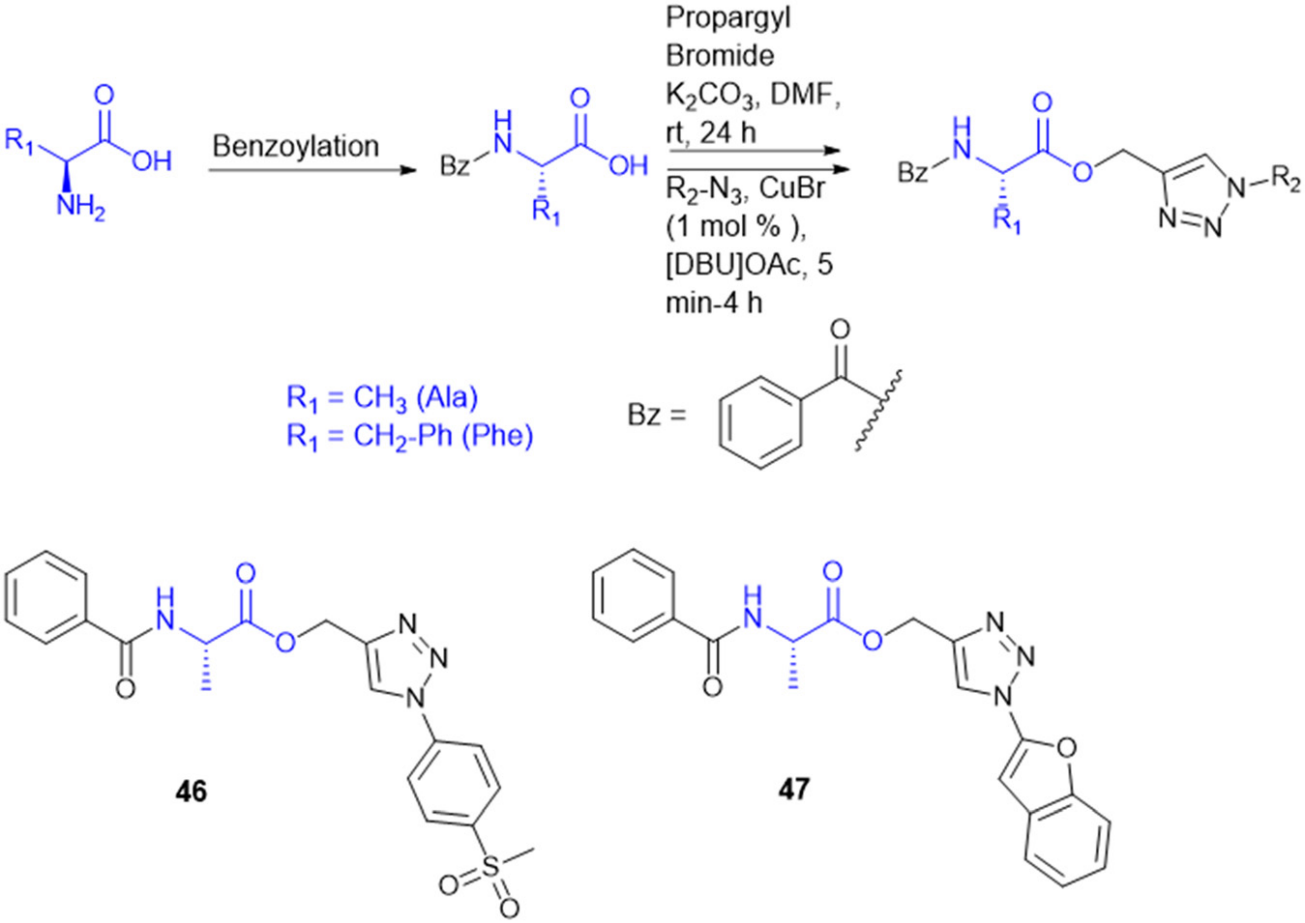
Synthesis of 1,4-disubstituted-1,2,3-triazole–amino acid conjugates.

A. Garg *et al.* synthesized novel amino acid containing 1,4-disubstituted 1,2,3-triazole molecules utilizing the ionic liquid [DBU]OAc (1,8-diazabicyclo[5.4.0]undec-7-ene acetate) under mild conditions at room temperature (Scheme 15). Among the twenty-one compounds studied, two compounds (46 and 47) with benzofuran and 4-(methylsulfonyl) phenyl moieties showed good antibacterial activity with an MIC value of 3.12 μg mL^−1^. Compound 46 exhibited the highest antibacterial activity against Gram-(+) and Gram-(−) bacterial strains. Compounds 46 and 47 were tested for cytotoxicity in bone marrow-derived macrophages; their selectivity index was 32.0, suggesting that they were not toxic compared to isoniazid (SI = 4000). Compounds 46 and 47 showed more selectivity than the first-line antitubercular drug ethambutol. Also, they exhibited favorable drug-likeness properties and good antibacterial activity.^134^

### 7.3. Amino acid conjugates as antiinfective agents

**Scheme 16:**
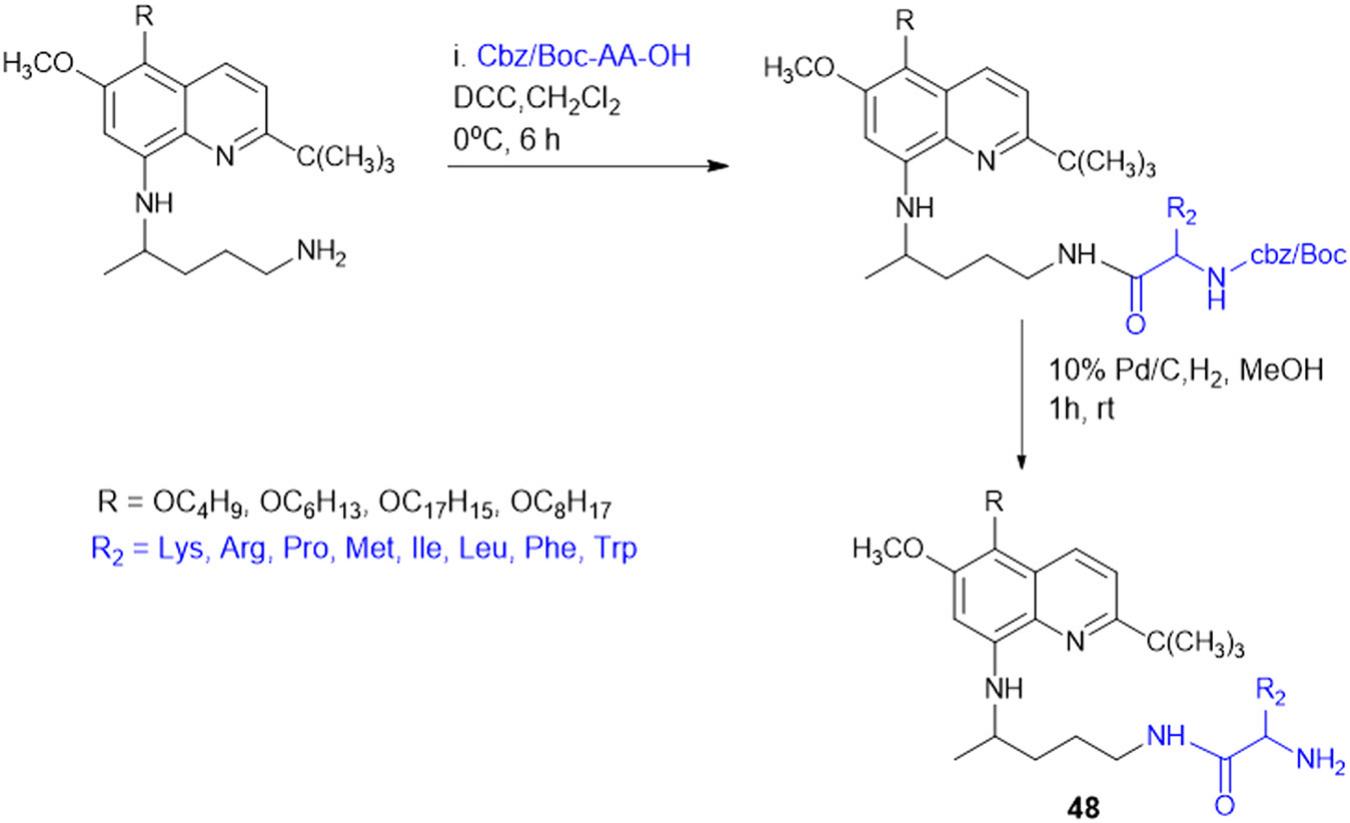
Synthesis of 8-quinolinamine–amino acid conjugates.

8-Quinolinamine derivatives conjugated with amino acids (48, Scheme 16) were synthesized and biologically screened as potential broad-spectrum antiparasitic agents directed against malaria and leishmaniasis. The synthesis adopts a multistep process to synthesize 6-methoxy-8-nitroquinolines through Skraup and phosphoric acid-mediated reactions followed by radical oxidative decarboxylation for 2-*tert*-butyl substitution. Reduction of the nitro group through RANEY® nickel-catalyzed hydrogenation provided the corresponding 8-aminoquinoline derivatives, which were alkylated using isoindolinedione-based linkers to form crucial precursors for amino acid conjugation. Hydrophobic and hydrophilic amino acids such as Lys, Arg, Leu, Phe, Met, and His were conjugated through dicyclohexylcarbodiimide (DCC)-mediated coupling reactions, improving biological activity and selectivity. The analogues exhibited antimalarial activity against both drug-sensitive (*Plasmodium falciparum* D6) and drug-resistant (*P. falciparum* W2) isolates, where the most efficacious analogue had IC_50_ values < 20 ng mL^−1^ that were comparable with those of chloroquine (CQ) and artemisinin (ART). With respect to basic amino acid conjugates, a number of compounds showed 100% cure rates against Swiss mice infected with *Plasmodium berghei* (25 mg kg^−1^ per day) and drug-resistant *Plasmodium yoelii nigeriensis* (50 mg kg^−1^ per day), validating their *in vivo* efficacy for inhibiting drug-resistant malaria strains. Conversely, antileishmanial assays showed that several 8-quinolinamine derivatives had IC_50_ values of 0.84–5.0 μg mL^−1^. Interestingly, basic amino acid conjugates lowered antimalarial activity, and hydrophobic amino acid modifications (Leu, Phe, and Met) increased antileishmanial activity, pointing to parasite-specific optimization against *Leishmania donovani*. Structural analysis further revealed that the presence of a methyl group at the C-4 position improved antileishmanial activity strongly while decreasing antimalarial potency. In addition, 8-quinolinamine amino acid conjugates were not active against antifungal activity, but these derivatives showed strong inhibition against *S. aureus* (IC_50_ = 1.33–18.9 μg mL^−1^), methicillin-resistant *S. aureus* (IC_50_ = 1.38–15.34 μg mL^−1^), and *Mycobacterium intracellulare* (IC_50_ = 3.12–20 μg mL^−1^). The most promising compound exhibited bactericidal activities with MIC values between 2.5 and 10.0 μg mL^−1^. Here, hydrophobic amino acid conjugates increased antibacterial activity, whereas basic amino acid modifications were less influential. The results introduce the 8-quinolinamine-based conjugates as suitable dual-targeting antiparasitic drug candidates, opening the gateway to further structural optimizations and improvements in therapeutic use.^135^

**Scheme 17:**
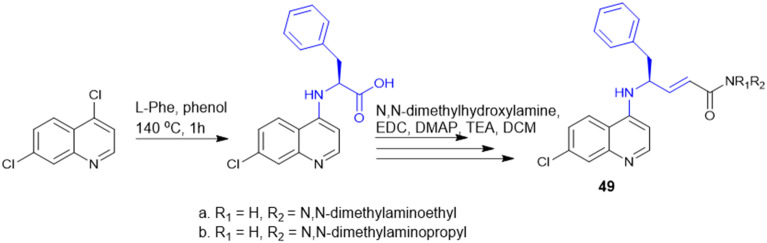
Synthesis of chloroquine derivatives.

Quinoline drugs such as chloroquine (CQ) are underscored as excellent antimalarial drugs, but over-extensive use of CQ leads to development of resistance by the malaria parasite *P. falciparum*. Thus, to overcome the CQ-resistance, Yeo J. S. group synthesised two novel derivatives by coupling 4,7-dichloroquinoline and Phe (Scheme 17), followed by modification that introduced a phenylmethyl group and α,β-unsaturated amide through Weinreb amide formation and the Horner–Wadsworth–Emmons reaction. Among the two CQ-conjugates, the *N*,*N*-dimethylaminoethyl containing derivative exhibited 1.28 fold higher efficacy than the CQ drug in CQ-resistant *P. falciparum*. Mice infected with *P. berghei* revealed that the CQ-conjugate with the *N*,*N*-dimethylaminoethyl group completely inhibited parasite growth and malaria-induced anaemia, increasing the survival rate from 40% to 100% over 12 days. The two key structure effects incorporated were identified: first, the larger phenyl group (from Phe) enabled better target binding than the smaller Ala derivative; second, modifying the alkyl group on the amide nitrogen allowed fine-tuning of anti-malarial activity based on size, bulkiness, and polarity offering a promising strategy for developing next-generation anti-malarial drugs.^136^

**Scheme 18:**
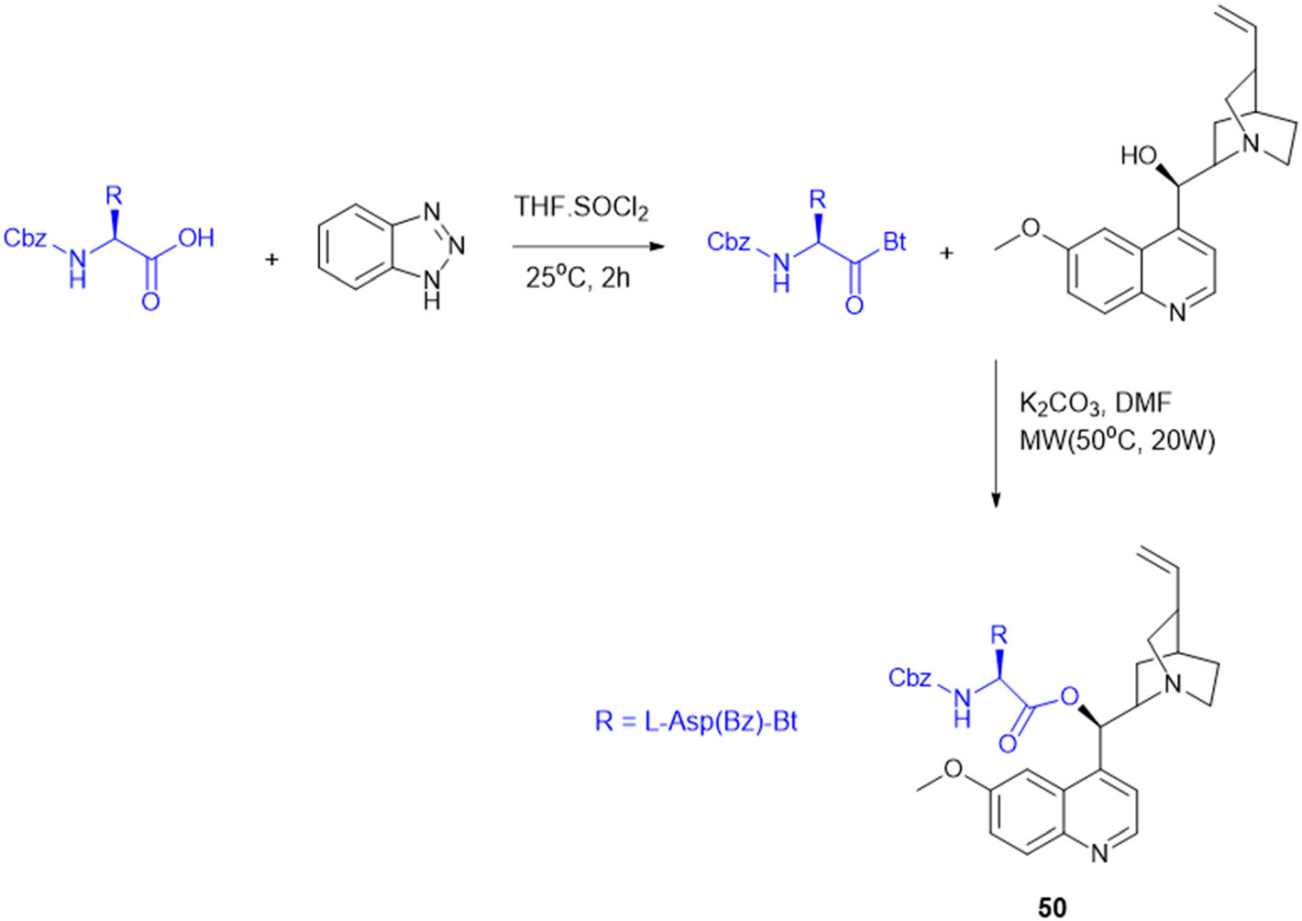
Synthesis of quinine–amino acid conjugates.

The synthesis and antimalarial bioassay of quinine–peptide conjugates explore the chemical strategy of amino acid and peptide conjugation to enhance quinine's antimalarial efficacy. These quinine–amino acid/peptide conjugates (Scheme 18) were synthesized through amide bonds by benzotriazole-mediated microwave-assisted coupling reaction. Among fourteen amino acid/peptide conjugated molecules, the *Z*-l-Asp(Bz)-QN (50) derivative exhibited the strongest activity (IC_50_ = 17 nM) compared to quinine (IC_50_ = 18 nM). The findings support the use of amino acid conjugation as a rational design strategy to boost the potency and selectivity of existing antimalarial drugs.^137^

**Scheme 19:**
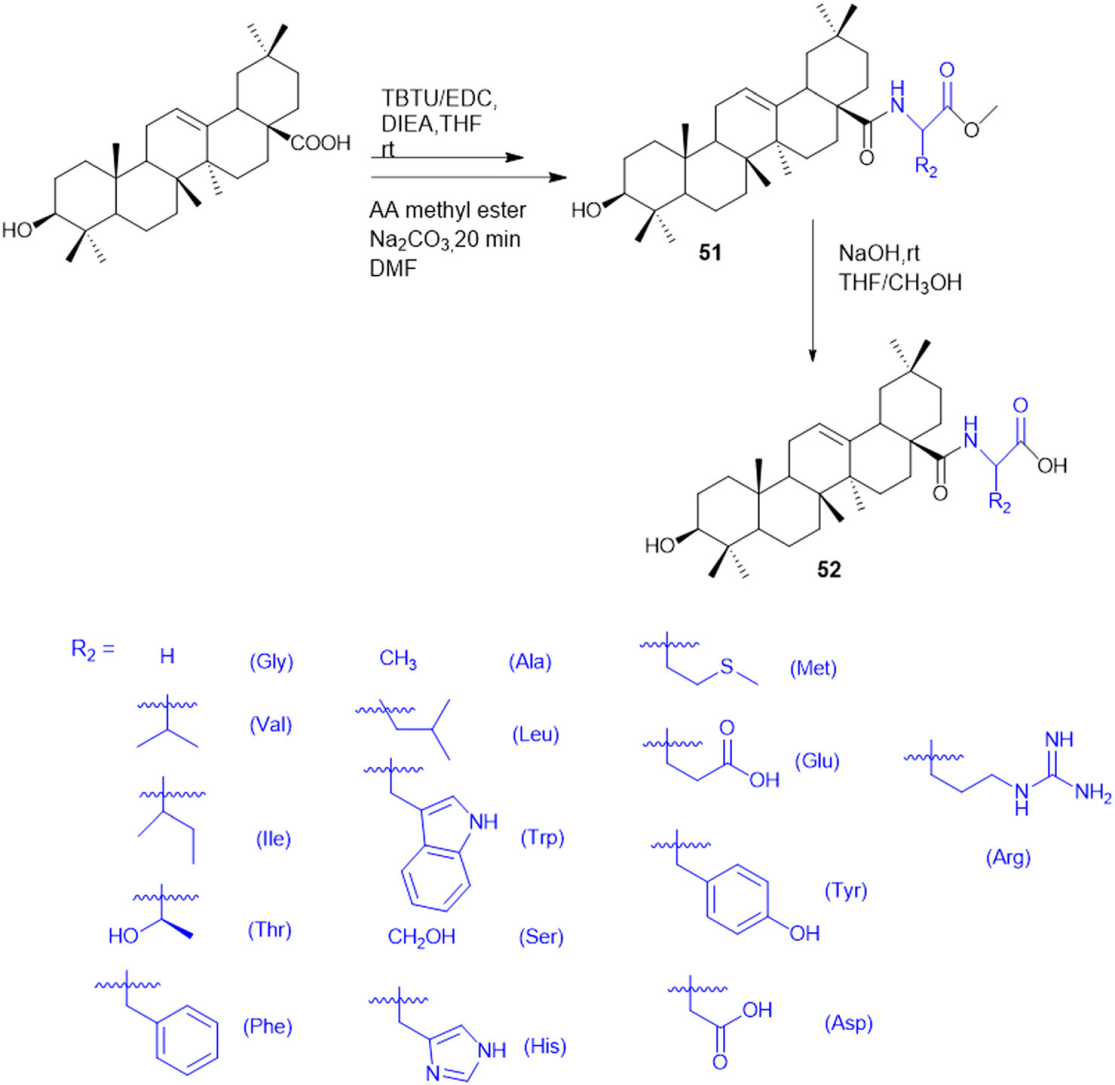
Synthesis of oleanolic–amino acid conjugates.

Meng *et al.* group synthesized and evaluated amino acid–oleanolic acid conjugates (52, Scheme 19) for anti-viral activity against influenza virus, based on their chemical properties for the antiviral mechanism. Overall, thirty-two oleanolic acid (OA) with C-28 carboxyl group conjugates of Met, Glu, Tyr, Asp, and Arg were synthesized *via* an EDCL/HOBt coupling reaction for amide bond formation. Among these, the OA–Arg conjugate exhibited the best inhibitory potency (with an IC_50_ of 6.64 μM) against H1N1 virus in addition to a broad anti-influenza spectrum for H3N2, BX-35, and BX-51B compared to oseltamivir phosphate. From the *in silico* studies, the hydrogen bonding of S193, A137, and Q226 residues with the OA–Arg conjugate at its conserved HA binding pocket was observed to be critical for sialic acid recognition. Thus, these finding highlights the OA–amino acid conjugates as promising influenza therapeutics, offering a novel strategy for targeting the viral entry mechanism and overcoming drug resistance challenges in influenza treatment.^138^

**Scheme 20:**
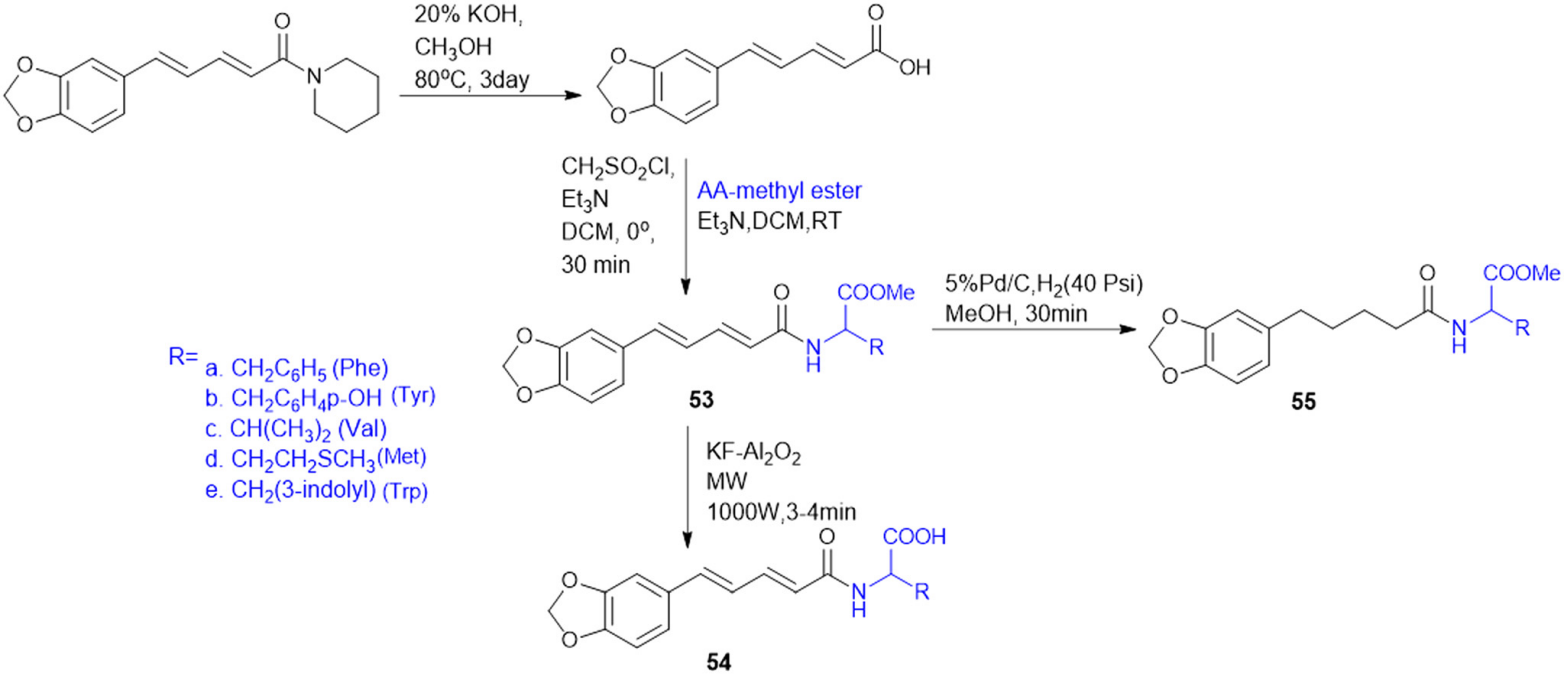
Synthesis of piperoyl–amino acid conjugates.

I. P. Singh *et al.*, synthesized a series of piperoyl–amino acid conjugates (Scheme 20), to identify new antileishmanial agent. Three sets of compounds: piperoyl–amino acid methyl ester conjugates, piperoyl–amino conjugates, and tetrahydropiperoyl–amino acid methyl conjugates were synthesized through amide bond formation between piperic acid and amino acid esters, followed by microwave-assisted deprotection or catalytic hydrogenation. The *in vivo* antileishmanial activity of a total of twenty-two molecules was tested against *L. donovani* promastigotes and amastigotes, and the most potent one was piperoyl–valine methyl ester 55c (IC_50_ = 0.075 mM against amastigotes). SAR analysis indicated that fluorine substitution and branched-chain amino acids improved binding affinity to bind *L. donovani* adenine phosphoribosyltransferase (APRT) to Ala150, Thr151, Gly152, and Thr154 *via* hydrogen bonding, coordinating with the Mg^2+^ ions, providing a possible inhibition mechanism. The results point to piperoyl–amino acid conjugates as a new avenue to develop possible antileishmanial drugs.^139^

### 7.4. Amino acid conjugates in cancer therapy

#### 7.4.1. Amino acid metabolism and transporters

Amino acids play a significant role as tumor regulators, interacting with the tumor microenvironment, signaling pathways, and epigenetic changes, among many other bidirectional interactions contributing to tumorigenesis.^140^ Moreover, metabolic reprogramming follows highly pervasive effects in cancer cells by maintaining an amino acid pool for fundamental processes, energy supply, and redox homeostasis.^141^ Pathways involved in macromolecular biosynthesis of proteins (60%), lipids (15%), and nucleic acids (5%) facilitate cell division and tumor progression, together constituting a significant portion of mammalian cell mass.^142^ Signaling pathways that regulate cell growth, particularly the PI3K-Akt-mTOR pathway, play a crucial role in controlling the biosynthesis of these macromolecules and are frequently activated in cancer due to tumorigenic mutations.^143^ These pathways start with the uptake of nutrients from the extracellular environment, which are then converted into biosynthetic intermediates through fundamental metabolic processes such as glycolysis, the pentose phosphate pathway (PPP), the tricarboxylic acid (TCA) cycle, and non-essential amino acid synthesis. Subsequently, larger molecules are assembled through ATP-dependent mechanisms.^144^ Cancer cells exhibit aerobic glycolysis, producing energy through glucose oxidation, or “the Warburg effect,” in many cancer cell types and tumors, following the generation of lactic acid. The efficacy of PET scans based on glucose transport for cancer diagnosis reinforces the idea that glucose transport and its utilization are at the core of cancer-cell metabolism.^145^ However, inhibiting the activity of pyruvate kinase for ATP production still fails to prevent tumorigenesis despite a high glycolytic rate in cancer cells wherein the mitochondrial metabolism revives to provide the essential TCA intermediates to refill the carbon requirement *via* the anaplerotic cycle.^146,147^ Also, amino acid and lipid dependencies supply metabolites to the TCA cycle, which sustains mitochondrial ATP production.^148^ Nine of the twenty amino acids, Lys, Trp, Phe, Met, Thr, Ile, Leu, Val, and His, are essential amino acids (EAAs), that are not synthesized by humans and must be obtained through diet. On the other hand, non-essential amino acids (NEAAs) like Gly, Ala, Pro, Tyr, Ser, Cys, Asn, Asp, and Glu can be synthesized by the body; moreover, during metastasis, *de novo* synthesis of NEAAs is increased to meet the nutrient burden in cancer progression.^149^

Amino acids possess hydrophilic properties, rendering them unable to pass through the plasma membrane unassisted and necessitating the involvement of selective transport proteins. Mammalian cells exhibit the differential expression of numerous amino acid transporters based on tissue-specific and developmental factors.^150^ Amino acid transporters (AATs) are membrane-bound solute carrier proteins that facilitate the movement of amino acids into and out of cells or cellular organelles. These transporters play various functional roles, including neurotransmission, acid–base balance regulation, intracellular energy metabolism, and the facilitation of anabolic and catabolic reactions. As per the HUGO Gene Nomenclature Committee (HGNC), the solute carrier (SLC) protein superfamily makes up more than half of all transport-related proteins and roughly 10% of the membrane proteins encoded by the human genome. With more than 400 annotated members and 66 families, it is recognized as the largest superfamily of membrane transporter proteins.^151,152^ P. Kandasamy *et al.* classified solute transporters based on substrate specificity and functional properties; AATs have been classified into various categories based on structural heterogeneity and coupling mechanisms such as system A (Na^+^-dependent pH sensitive cotransporter), N (amino acids with nitrogen atoms in the side chain), ASC (alanine/serine/cysteine-preferring transporters), B (transporter accepting neutral amino acid), L (Leu-preferring transporter), T (aromatic amino acids), x^−^_ct_ (X-anionic amino acids), y^+^ (cationic amino acids), the GltPh acidic AAT superfamily, the amino acid polyamine (APC) (d-Ser) superfamily, the major facilitator superfamily (MFS), the mitochondrial carrier family (MCF), and the SWEET PQ-loop AAT superfamily.^153^

**Fig. 6:**
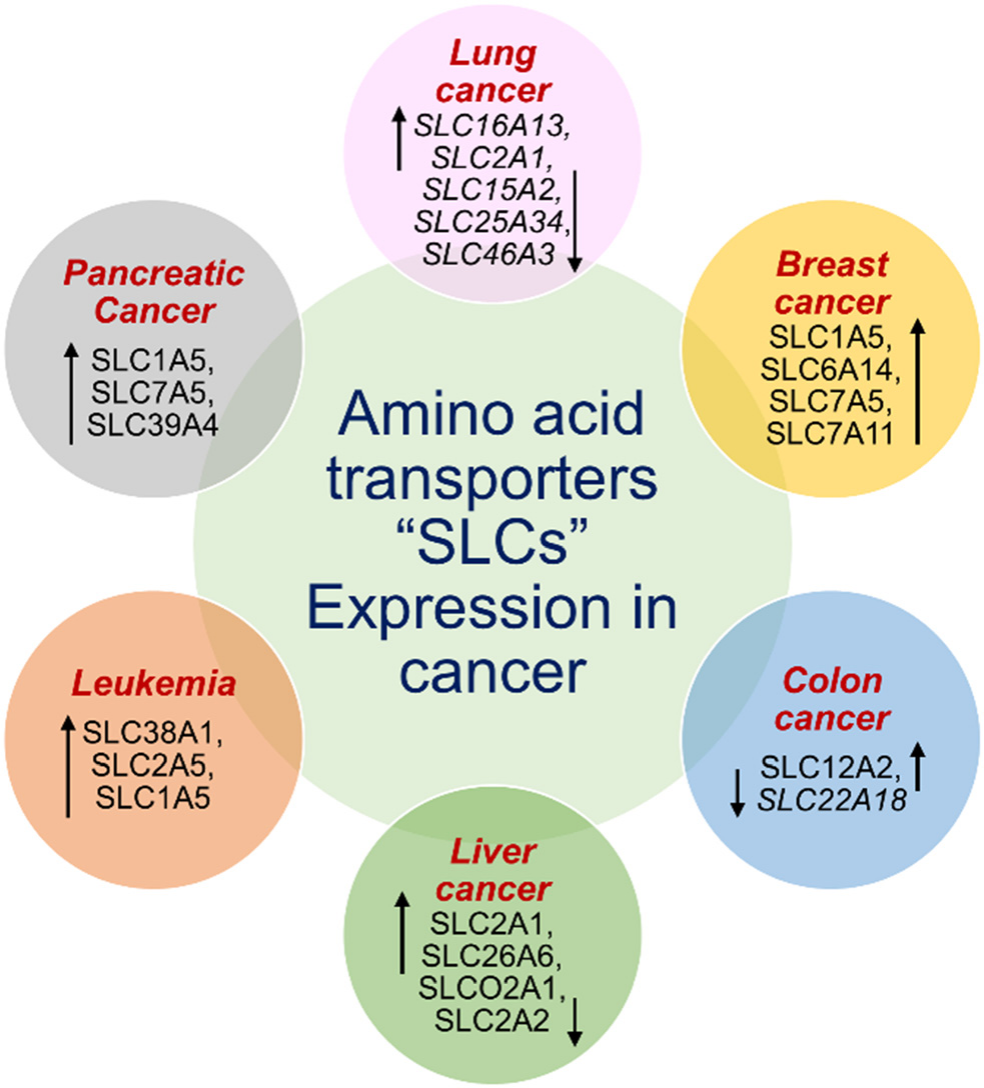
Expression of SLCs in cancer subtypes.

**Fig. 7:**
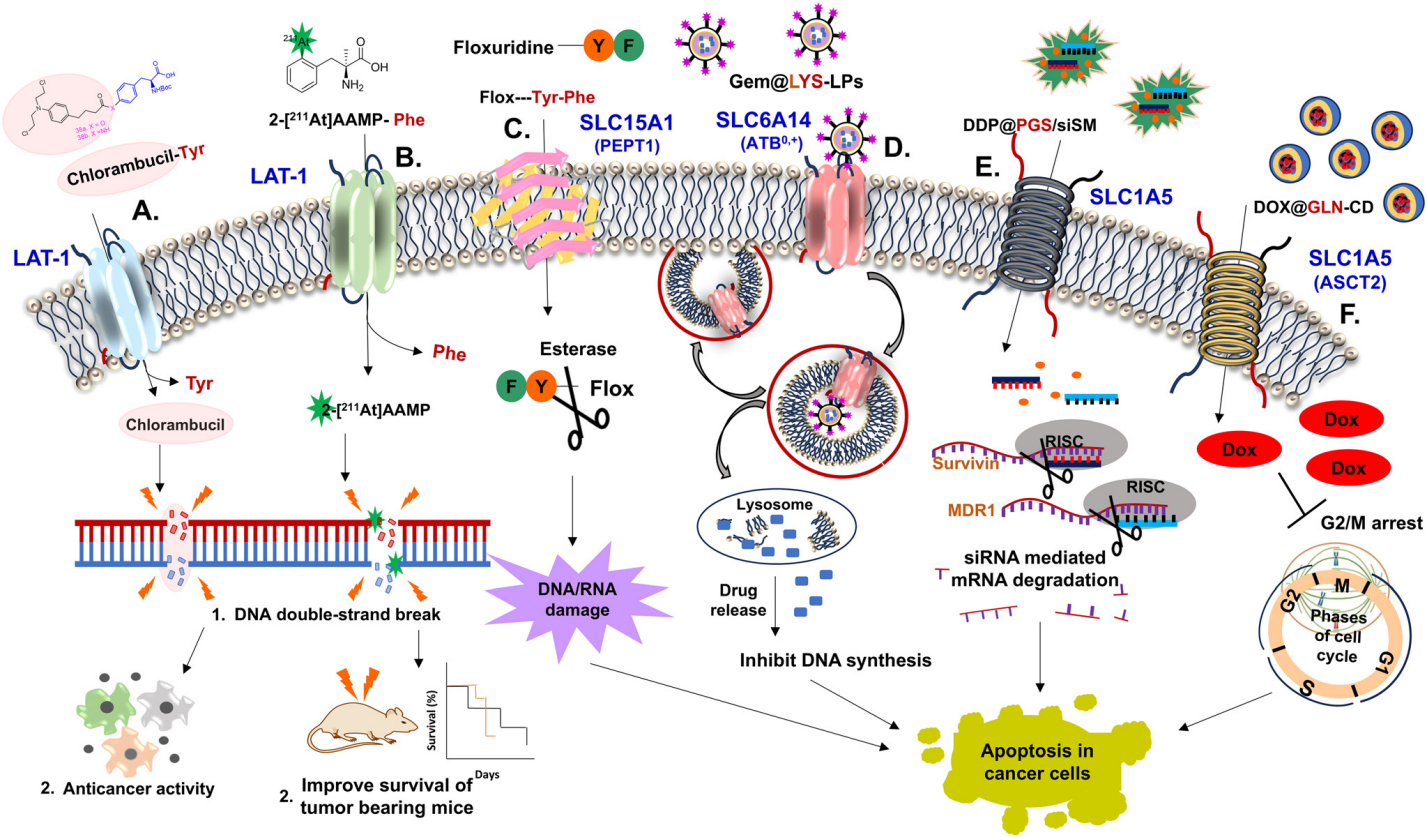
Targeted drug delivery using amino acid bioconjugates *via* SLC transporters. Abbreviations: l-type amino acid transporter 1 (LAT-1), peptide transporter 1 (PEPT1), RNA-induced silencing complex (RISC), small interfering RNA (siRNA), floxuridine (Flox), and alanine, serine, cysteine transporter 2 (ASCT2).

AATs are expressed on cell membranes that monitor the concentration of amino acids and facilitate the exchange of amino acids between extra- and intracellular environments. The transport of amino acids is coupled to ion movements (Na^+^, H^+^, K^+^, and/or Cl^−^) based on the movement of other amino acids through anti-port and symport pathways.^154^ SLC transporters function as the “metabolic gate” of cells, facilitating the transfer of vital nutrients and metabolites, including glucose, amino acids, vitamins, neurotransmitters, and inorganic/metal ions. SLC transporters have been identified as causal genes in human genomic studies for various illnesses, including cancer, metabolic diseases, cardiovascular ailments, immunological problems, and neurological dysfunction.^155^ Apart from SLCs, anionic and cationic amino transporters mediate amino acid transport activity and exhibit overlapping expression patterns with their substrate specificities. Nutrient signals can be triggered by the amino acid concentration in a particular subcellular region when combined with the compartmentalized nature of amino acid metabolism.^156^ SLC transporters exhibit coordinated expression patterns in healthy tissues, suggesting a well-regulated network governing typical cellular processes. Tumour cells, however, change the expression of SLC transporters to meet their increased nutritional demands due to metabolic changes that occur during tumor development.^157^ Among numerous AATs, SLC1, SLC3, SLC6, SLC7, SLC15, SLC17, SLC18, SLC25, SLC32, SLC36, and SLC38 have been shown to serve as transporters of amino acids.^158,159^ Now, there is increasing evidence that dysregulation of SLCs is associated with several diseases of cancer, neurological disorders, and autoimmune diseases such as rheumatoid arthritis. Their membrane localization and druggability qualify them as good targets for small-molecule or prodrug design. In addition, the modulation of SLC activity presents an approach to overcome drug resistance by changing intracellular drug levels or metabolic dependencies. The expression levels of different SLCs in different cancer subtypes are shown in Fig. 6. SLCs are expressed differentially in the tumor microenvironment due to altered requirements that render them targeted in the treatment of cancer. By facilitating the effective transport of drugs into cancer cells, these strategies will be crucial in the future development of anticancer drug therapy (Fig. 7).

#### 7.4.2. SLC mediated drug delivery in cancer therapeutics

Overexpression of l-type amino acid transporters (LAT-1/SLC7A5) is well-reported in breast, prostate, lung, and ovarian cancers, which promotes cancer growth *via* mTOR signaling.^141^ From recent nanotherapeutics advancement as a targeted α-radionuclide therapy LAT1-selective α-radionuclide-labeled amino acid analog, 2-[^211^At]astato-α-methyl-l-phenylalanine (2-[^211^At]AAMP) was synthesized. From *in vitro* assessment in LAT-1^+^ cancer cells (SKOV3 cells; ovarian cancer cells), 2-[^211^At]AAMP treatment suppressed clonogenic growth (10 kBq mL^−1^) and induced cell death at 25 kBq mL^−1^ (unit of standard uptake value). Furthermore, 2-[^211^At]AAMP markedly increased the survival rate of mice harbouring tumors indicating that 2-[^211^At]AAMP may help enhance the therapeutic impact of positive malignancies for LAT-1.^160^

**Scheme 21:**
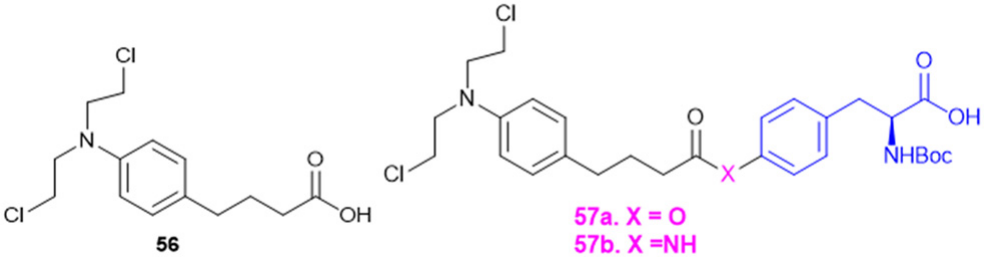
Chlorambucil–tyrosine conjugated prodrugs.

The amino acid conjugating prodrug approach also showed promising effective therapy against LAT-1^+^ cancer cells. L-Tyr, a natural LAT-1 substrate, was conjugated to chlorambucil by an ester or an amide bond (compound 56 or 57) which increased cellular uptake *via* the LAT-1 transporter overexpressed in breast cancer cells (MCF-7). Compounds 56 and 57 (Scheme 21) bound to LAT-1 similarly to l-Tyr, indicating efficient recognition and transport by the transporter. Treatment with derivatives 56 and 57 exhibited significantly higher antiproliferative activity and cell cytotoxicity with 25.8% ± 3.2% for 57a, 18.0% ± 2.5% for 57b, and 10.6% ± 1.3% for chlorambucil 56 alone. This outcome suggests that conjugation to the LAT-1 substrate facilitated enhanced cellular uptake of the derivatives.^161^

**Scheme 22:**
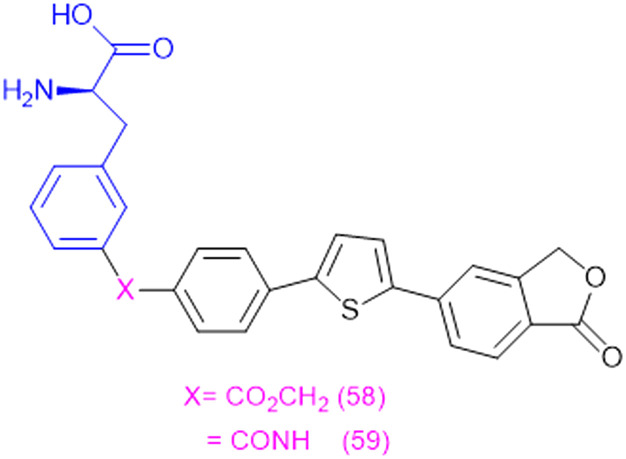
LAT-1 utilizing prodrug of perforin.

LAT-1 using prodrugs improves drug delivery into cancer cells by preventing the cellular uptake of [^14^C]-l-Leu, where all prodrugs exhibited good LAT-1 affinity. Compared to their parent medicines, prodrugs 58 and 59 (Scheme 22) showed higher penetration into MCF-7 cells *via* LAT-1. Additionally, these prodrugs use a different probenecid-sensitive transport mechanism at higher doses or with LAT-1 saturation. In liver fractions, ester prodrugs released their parent drugs enzymatically, whereas amide prodrugs are anticipated to do the same *in vivo*. Targeting LAT-1-overexpressing cancer cells finds potential use in the prodrug-efficient delivery of perforin inhibitors to lower the pH of cell organelles. These results encourage more research into the intracellular action of prodrugs and other transport pathways.^162^

P. Zohu *et al.* developed a doxorubicin (DOX) inclusion complex with a glutamine-β-cyclodextrin (Gln-CD) derivative as DOX@Gln-CD to target TNBC. By conjugating Gln to 6-hydroxy of β-cyclodextrin, Gln-CD entered ASCT2/SLC1A5 transporter overexpressed TNBC cells (MDA-MB-231 and BT549) where the Gln-CD and DOX complex (DOX@Gln-CD) induced G2/M arrest and apoptosis. The absorption of DOX@Gln-CD in TNBC cells was decreased by the ASCT2 inhibitor GPNA, indicating the involvement of ASCT2 in its transport. Compared to DOX alone, *in vivo* DOX@Gln-CD exhibited better treatment results, less toxicity to major organs, and a selective accumulation in tumors, establishing Gln-CD as an efficient carrier for targeted drug delivery in TNBC.^163^

Delivering siRNA gene regulators to Glu-dependent cancer cells involves polyglutamine (PGS), a glutamine analog. In cisplatin-resistant lung adenocarcinoma cells (A549/DDP), the overexpressed Gln transporter (SLC1A5) exhibited a strong affinity for PGS. Cancer cells exhibited considerable uptake of PGS/siRNA complexes due to low glutamine levels in tumors. PGS uptake was decreased when SLC1A5 was inhibited by raloxifene (SLC1A5 inhibitor) demonstrating its function in cellular internalization. PGS decreased intracellular Glu levels, slowing down cell division and further leading to increased expression of SLC1A5. When combined with cisplatin, PGS/siRNA significantly reduced the development of lung tumors *in vivo*, indicating a possible method for lung cancer treatment that targets glutamine metabolism.^164^

Lysine-conjugated liposomes (Lys-LPs) target the highly expressed SLC6A14 (ATB^0,+^) transporter in breast and pancreatic cancer cells. Lys-LPs, which rely on Na^+^/Cl^−^ dependent binding and endocytic internalization mechanisms, exhibited increased uptake in ATB^0,+^ positive MCF-7 cells compared to unmodified liposomes. This uptake was inhibited by the ATB^0,+^ inhibitor α-methyl-d, l-tryptophan (α-MT), a selective blocker of SLC6A14, indicating selectivity. Lys in Lys-LPs promoted initial binding and internalization, resulting in a temporary endosomal breakdown. Additionally, in an ATB^0,+^ dependent way, Lys-LPs improved pancreatic cancer therapy by targeted drug delivery.^165^

The PEPD gene encodes prolidase (peptidase D), a cytosolic metalloproteinase that cleaves imidodipeptides containing C-terminal proline or hydroxyproline of the extracellular matrix (ECM).^166^ Prolidase activity is regulated by the ECM and the β1 integrin-triggered Ras-Raf-MEK-ERK pathway, which is crucial for collagen recycling.^167^ Recent studies have shown that prolidase also acts as an external ligand for EGFR and ErbB2 (HER2) receptors, activating downstream signaling pathways like Akt, STAT3, and ERK that stimulate transcription of markers associated with differentiation and cell growth.^168,169^ The clinical significance of prolidase with higher expression is evident during pathological conditions of various skin, breast, lung, ovary and endometrium malignancies. As a result, it gained attention for site-specific prodrug conversion.^170^ Considering the prolidase activity among cancer subtypes, it serves as the basis for using Pro in pharmaceutical drug design. Melphalan is an anticancer medication that stops cell division by alkylating and cross-linking guanine and, perhaps, other bases in DNA. K. Chrzanowski *et al.* synthesized a prolidase substrate by conjugating melphalan with Pro through an imido bond, showing better efficacy against breast cancer cells (MDA-MB231).^171,172^ Due to the overexpression of prolidase and the problem of MTX resistance in various cancer cells, MTX–Pro conjugation is used to effectively enhance the efficacy of drug delivery compared to MTX.^172^

**Scheme 23:**
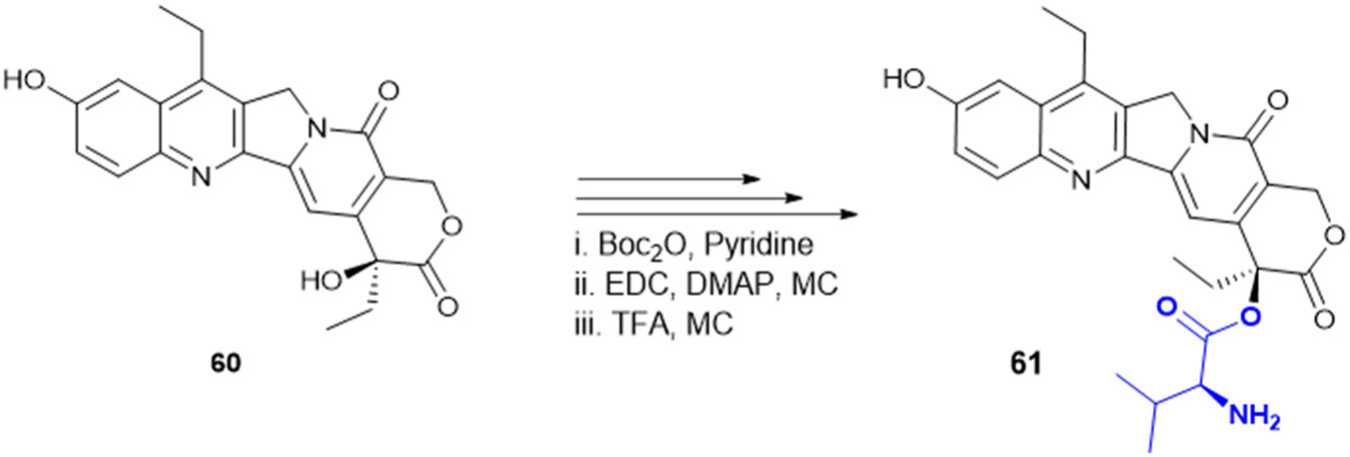
Synthesis of the Val-conjugated prodrug of SN-38.

During cancer relapses, the anticancer agent is effluxed out by the cancer cells attaining multi-drug resistance. Specific moieties have been utilized to design prodrugs to accumulate inside cancer cells. Val–SN-38 (61), an active ingredient in irinotecan, is designed by adding Val to SN-38 (60) (Scheme 23). 61 accumulated intracellularly in MCF-7 cells approximately five-fold more than its parent molecule, SN-38. This increase was attributed to the amino acid transporter ATB^0,+^ rather than alterations in membrane permeability. In addition, sodium-dependent amino acid transporters, specifically ATA1, ATA2, and ASCT2, were also engaged in the cellular uptake of 61. However, 61 contains an ester linkage, which is enzymatically unstable from esterase *in vivo*, whereas the 60 E-ring is essential to its cytotoxic effect. Therefore, amide linkages offer more stability and can be considered a better alternative.^173^

**Scheme 24:**
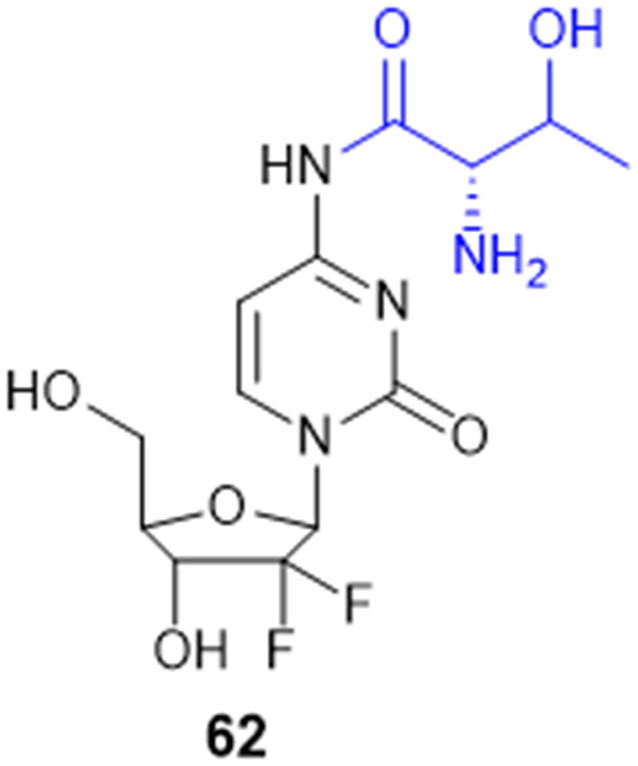
Structure of the gemcitabine–Thr prodrug.

Gemcitabine is a standard anticancer agent used to treat all stages of pancreatic adenocarcinoma with erlotinib and nab-paclitaxel in combinational therapy. S. Hong *et al.* synthesized another set of gemcitabine amino acid conjugated prodrugs such as Gem–Thr, Gem–Tyr, Gem–Val, Gem–Met, Gem–Ile, and Gem–Leu. Among all conjugates, Gem–Thr demonstrated greater potency than gemcitabine against the LAT-1 overexpressed cell line BxPC-3, MIAPaCa-2 (pancreatic cancer cells), and B16 (melanoma cells). Gem–Thr (62) (Scheme 24) poses anticancer activity with 44.7% (Gem–Thr) *vs.* gemcitabine, 54.1% of cell viability in BxPC-3 cells (*p* = 0.0464). The amide bond in the Gem–Thr conjugation improves metabolic stability and increases systemic exposure by 1.83-fold compared to free gemcitabine. Thus, Thr in a conjugate significantly improves the pharmacokinetics of a free drug and offers a promising treatment against cancer.^174^

K. Bahrami *et al.* (2023) explained the structural and kinetic understanding of amino acid conjugates with LAT-1. They used LAT-1 overexpressing HEK293 cells (HEK-hLAT1) and control HEK-MOCK cells, and performed uptake kinetics and induced fit docking to know how linker chemistry, the payload structure and molecular flexibility play crucial roles in interaction of phenylalanine conjugates with LAT-1 mediated uptake. The α-amino group (NH_3_^+^) and α-carboxylate (COO^−^) of the Phe moiety anchor the amino acid conjugate *via* hydrogen bonding to the TM1 GSG (Gly65-Ser66-Gly67) motif and TM6 residue (Gly255-Phe252-Asn258) and the aromatic payload interacts with TM10 (Phe400/Asn404) and TM3 (Glu136/Ser144) *via* π–π interaction. Smaller amino acids with torsional freedom between aromatic moieties show the highest uptake; the rigid planar structure reduces transport. The linker also plays a major role; the *para*-carbamate linker improves transport over the direct amide linker. Polar spacers between aromatic rings enhance hydrogen bonding and allow better accommodation in the LAT-1 binding pocket. However, highly lipophilic amino acid conjugates (naproxen/ flurbiprofen) tend to bypass LAT1 *via* passive diffusion. The highly selective l-DOPA phenylalanine conjugate exhibits a *V*_max_ of 1521 pmol min^−1^ mg^−1^ and a *K*_m_ of 100 μM in HEK-hLAT with increased 50-fold selectivity as compared to the control HEK-MOCK cells^175^ (Table 4).

**Table 4:** Docking poses of compounds to the LAT-1 cryo-EM structure and their kinetic parameters (*K*_m_, *V*_max_); data correspond to different cell lines^a^^,^^b^^,^^c^

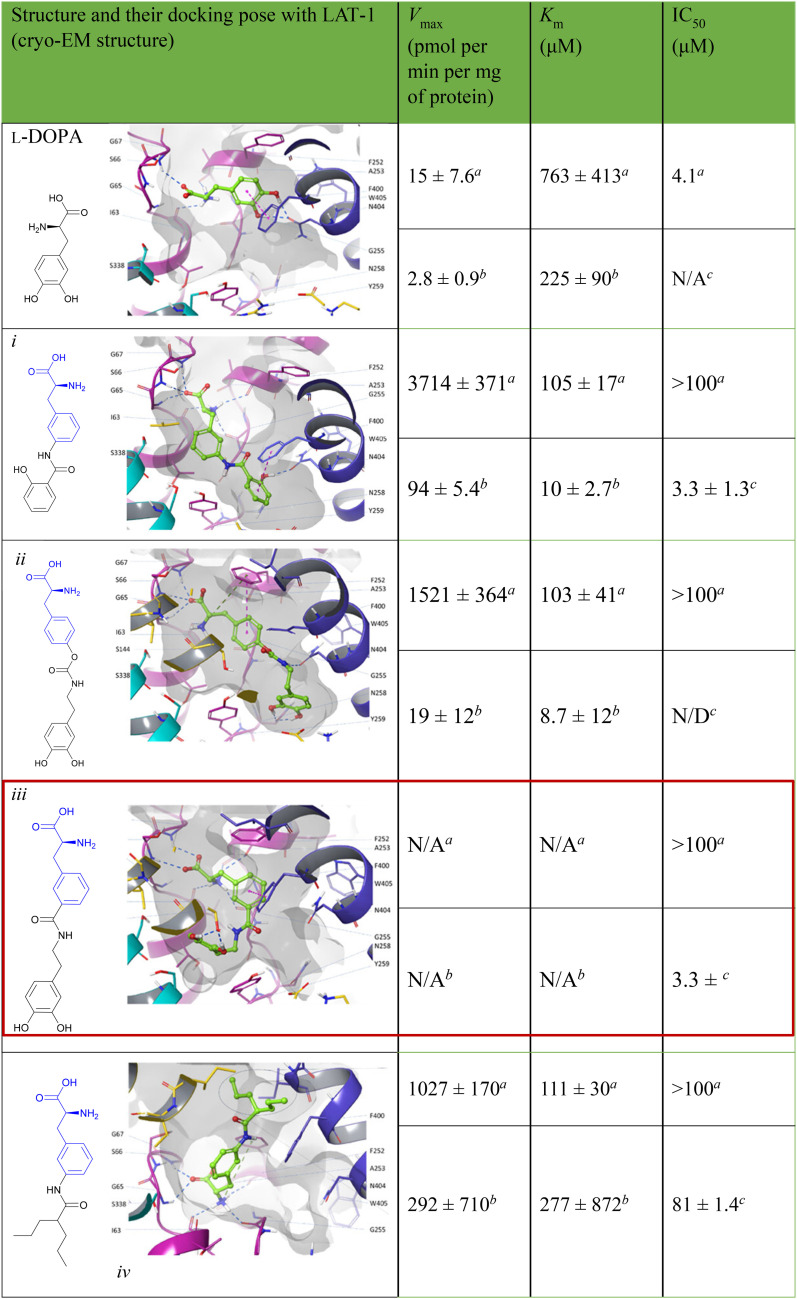

a HEK-hLAT1 cell line.

b HEK-MOCK cells.

c MCF-7 cells. The figure was reproduced with permission from ref. 171. Copyright 2023, American Chemical Society.

Y. Lee *et al.* (2025) explained the interaction mechanism of JPH203 which has a Phe moiety. It constitutes LAT-1 and a heterodimeric complex with CD98hc protein that is specifically involved in the transport of large neutral amino acids as well as structurally related drug conjugates. From the latest high-resolution cryo-EM structure, it is revealed that LAT-1 possesses a central binding pocket within the transmembrane helices (TM1 and TM6), where a number of conserved amino acids, including Ile63, Ser66, Gly67, Phe252, and Gly255, bind to the amino and carboxylic groups of the substrate. The LAT-1 substrate reorganization flexibility is due in most part to its side chain binding side, which consists of hydrophobic binding residues like Phe252, Trp405 and Ile139. The pocket is large enough to hold a bulkier side chain, including those present in amino acid-based conjugates. The location of small amino acids like Gly255 close to the pocket is responsible for the flexibility of the pocket so that it can change its conformation and accommodate a broad group of substrates. This flexibility is the reason behind the high transport efficiency of LAT-1 for both amino acid and bulky drug conjugates, including the melphalan-based amino acid conjugate and l-DOPA.^176^

Amino acid conjugates have shown promising anticancer activity in various studies. The conjugation of an amino acid with a heterocyclic motif provides novel potent molecules to enhance efficacy solubility and mitigates toxicity across diverse products. A series of amino acid conjugates of 2-aminothiazole and 2-aminopyridine molecules were synthesized to target multiple cellular pathways in cancer. Gly, Phe, Leu, and Ile were used in the synthesis, and the conjugated molecules were subsequently evaluated for their antioxidant and anticancer activities in A2780 (ovarian cancer cell line) and A2780CISR (cisplatin-resistant ovarian cancer cell line). Compound 63 showed the best activity with an IC_50_ value of 15.57 μM against the A2780 cell line and 11.52 μM against the A2780CISR cell line with a resistance factor of 0.74. However, cisplatin showed 0.61 μM and 16.43 μM for the A2780 and A2780CISR cell lines, respectively, with a resistance factor of 26.93. Varied effects on several proteins were evaluated by docking studies with EGFR, VEGFR, PDGFR, p^110a^/PI3K, RET, BCR-ABL, c-KIT, c-Raf, B-Raf, and CTLA-4, proteins expressed in cisplatin-sensitive and -resistant cancer cells. Compound 63 (Table 5) showed multiple hydrogen bonding interactions with PDGFR, c-KIT, and ALK with the lowest binding energy values of −5.2 kcal mol^−1^, −8.1 kcal mol^−1^, and −7.5 kcal mol^−1^, respectively.^177^ Amino acid transporters supply essential amino acids to cancer cells, making them useful targets for tumor-targeting therapy. In this context, designing noscapine amino acid conjugates of Ala, Val, Phe, Leu, and Trp as prodrug offer enhanced selectivity, and minimizing off-target effects. These conjugates were synthesized and screened against lung cancer cell line (A549). Among them, noscapine–Trp 64 conjugations followed better activity with IC_50_ – 32 μM than noscapine alone with IC_50_ – 73 μM and arrested the G_1_ phase of the cell cycle. Molecular docking and dynamics simulations revealed strong interactions and stable complex formation between tubulin and the noscapine–Trp complex without disrupting tubulin's active site. Thus, Trp-conjugated prodrugs enhanced anticancer activity.^178^

**Table 5:** Amino acid containing anticancer molecules

Compound	Amino acid used for conjugation	Amino acid with the highest activity	Cell line (IC_50_)
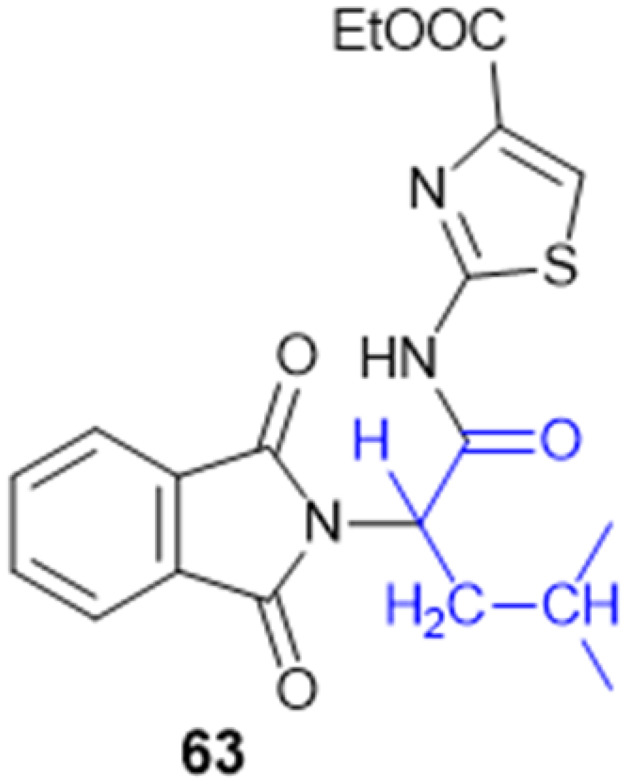	Gly, Phe, Leu, Ile	Leu	A2780 cells (15.57 μM) and A2780 CISR (cisplatin resistance) (11.52 μM)
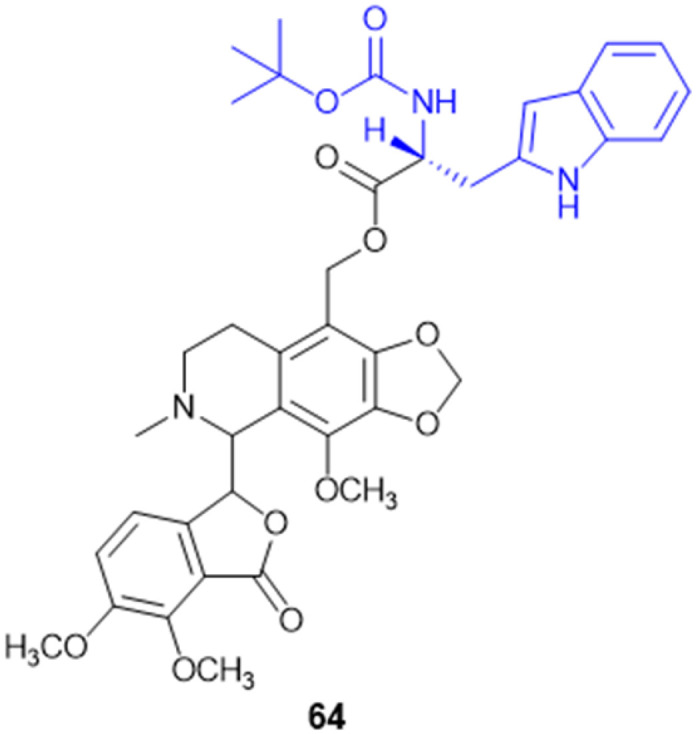	Trp, Ala, Val, Phe, Leu	Trp	A549 cells (32 μM)
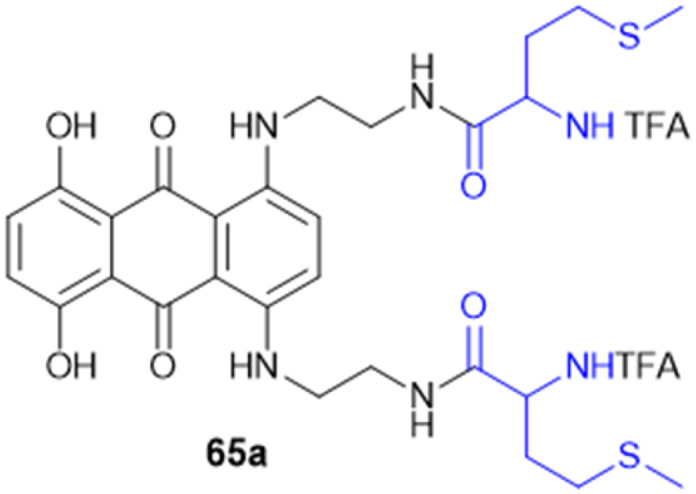	Met, Ser, Tyr, Lys	Met, Lys	MCF-7 (1.64 μM), NCI-H460 (0.38 μM), SF-268 (0.35 μM), PC-3 (0.28 μM) NCI-H460 (1.38 μM), SF-268 (2.03 μM), PC-3 (0.83 μM)
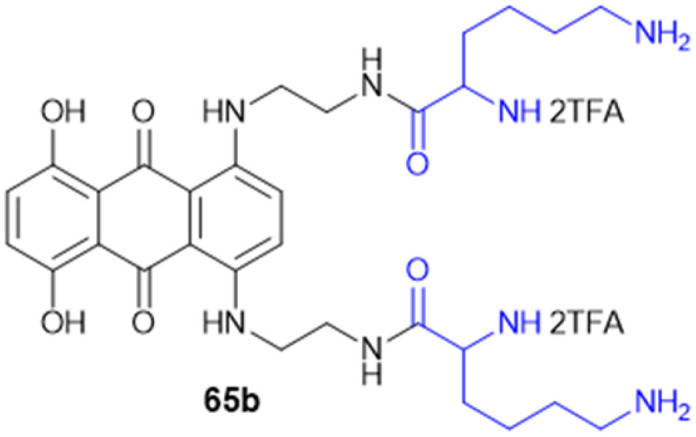			
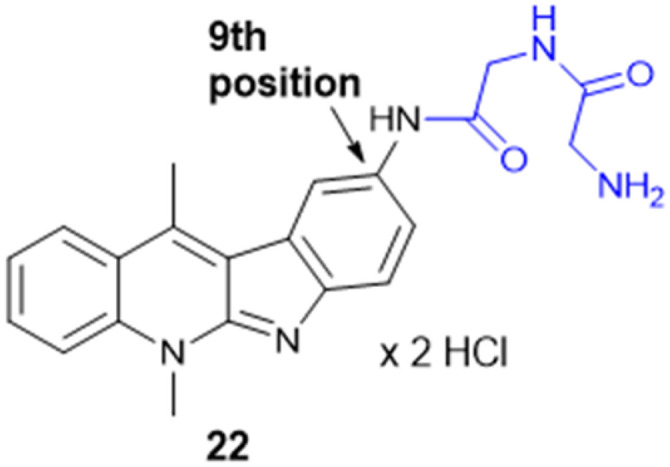	Gly-Gly, l-Pro, His, Gly, His-Gly, Pro-Gly	Gly-Gly	A549 (0.90 μM), MCF-7 (2.70 μM), LoVo (0.73 μM)
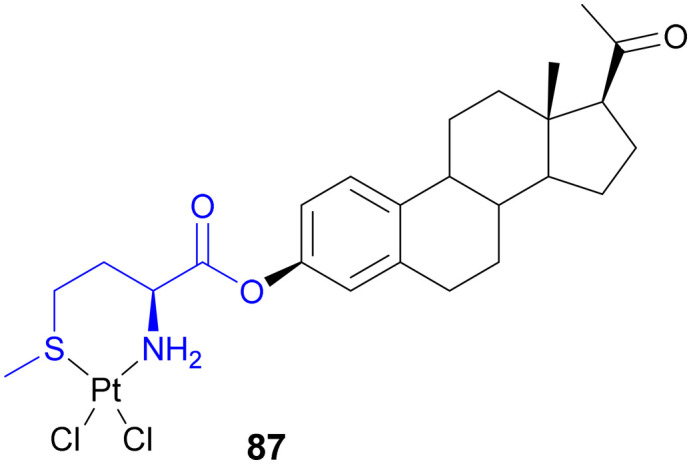	Met, His	Met	T-lymphoblastic leukemia (CEM) (18.1 μM), MCF-7 (>50 μM), A-549 (43.1 μM), RPMI-8226 (32.2 μM) (lymphoblast like multiple myeloma), BJ (>50 μM)
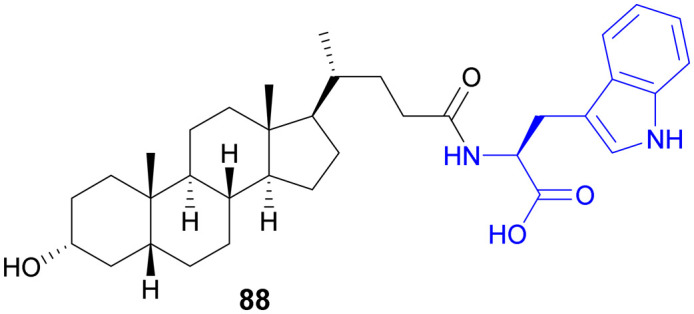	Gly, Ala, Val, Thr, Met, Phe, Tyr, Trp, Asp	Trp	PC3 cell (pIC_50_ = 5.69)
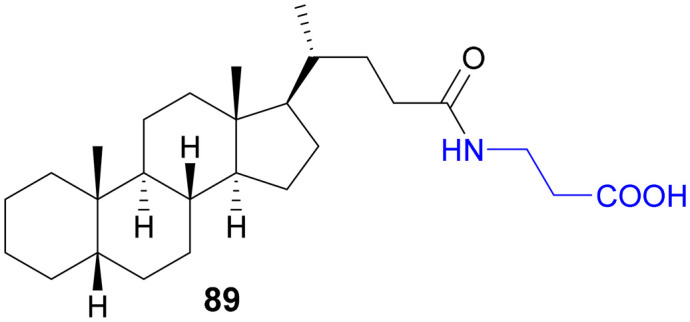	Trp, Phe, Gly, Ala, β-Ala	β-Ala	PC3 cell (pIC_50_ = 4.82)

Amino acids are also employed to modify pharmacological properties. In 1,4-bis(2-amino-ethylamino) anthraquinone–amino acid conjugates (BACs), ametantrone (AT)–amino acid conjugates (AACs) and mitoxantrone (MX)–amino acid conjugates (MACs) have been reported with DNA binding and cytotoxicity activity against MCF-7, NCI-H460, SF-268, and PC-3 cell lines. l-Met-MAC (65a) and l-Lys-MAC (65b) showed the maximal inhibitory activity (Table 5). In contrast, l-Met-MAC (65a) showed significantly less DNA binding activity with the highest cytotoxic activity. l-Lys-MAC (65b) showed higher DNA binding with reduced cytotoxic activity compared to l-Met-MAC.^179^

K. Sidoryk *et al.* designed and synthesized derivatives of neocryptolepine with an amino acid or a dipeptide at the C-9 position (22; Table 5). 5*H*-Indolo[2,3-b]quinoline (DiMIQ) has potent antitumor activity but shows severe toxicity. Amino acids like Gly, Pro, and His alter the physiological properties of neocryptolepine. When the hydrophilic peptide or amino acid was attached to the hydrophobic DiMIQ, the conjugate was more hydrophilic and less hemolytic. A screening of the synthesized conjugates against cancer cell lines (A549, MCF-7, and LoVo) and BALBc/BT3 mice showed the glycylglycine derivative as the most promising conjugation.^180^

**Scheme 25:**
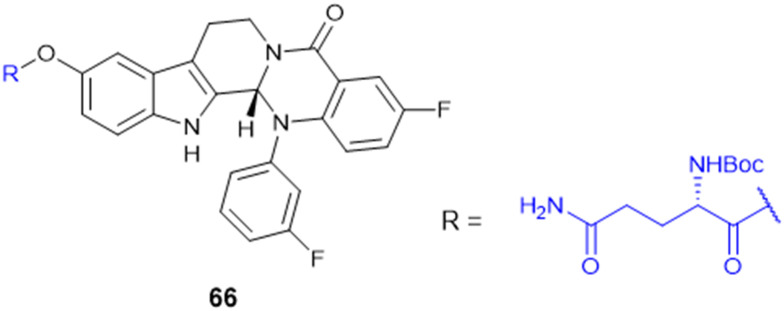
Evodiamine–amino acid conjugate.

Evodiamine (Scheme 25) is a bioactive quinolone alkaloid with broad antitumor activities but low aqueous solubility and metabolic instability that limit its application. To overcome these challenges, S. Chen *et al.* developed *N*-Boc amino acid derivatives of evodiamine as EDCI/HOBt coupling agents that enhanced its druggability and anticancer properties. Among them, the *N*-Boc-l-Glu (66) derivative showed the greatest potency, by greatly enhancing tumor cell uptake and retention, surpassing the parent compound with nanomolar IC_50_ values against LoVo and RKO colorectal cancer cell lines. Mechanistic studies confirmed that the derivative efficiently inhibited topoisomerase I, causing cell cycle arrest at the G2/M phase, and initiated apoptosis through mitochondrial membrane disruption. In LoVo and HT-29 xenograft mouse models, amino acid-modified evodiamine showed better antitumor activity than the control compound, revealing its potential as a candidate to be further developed in targeted cancer therapy.^181^

**Scheme 26:**
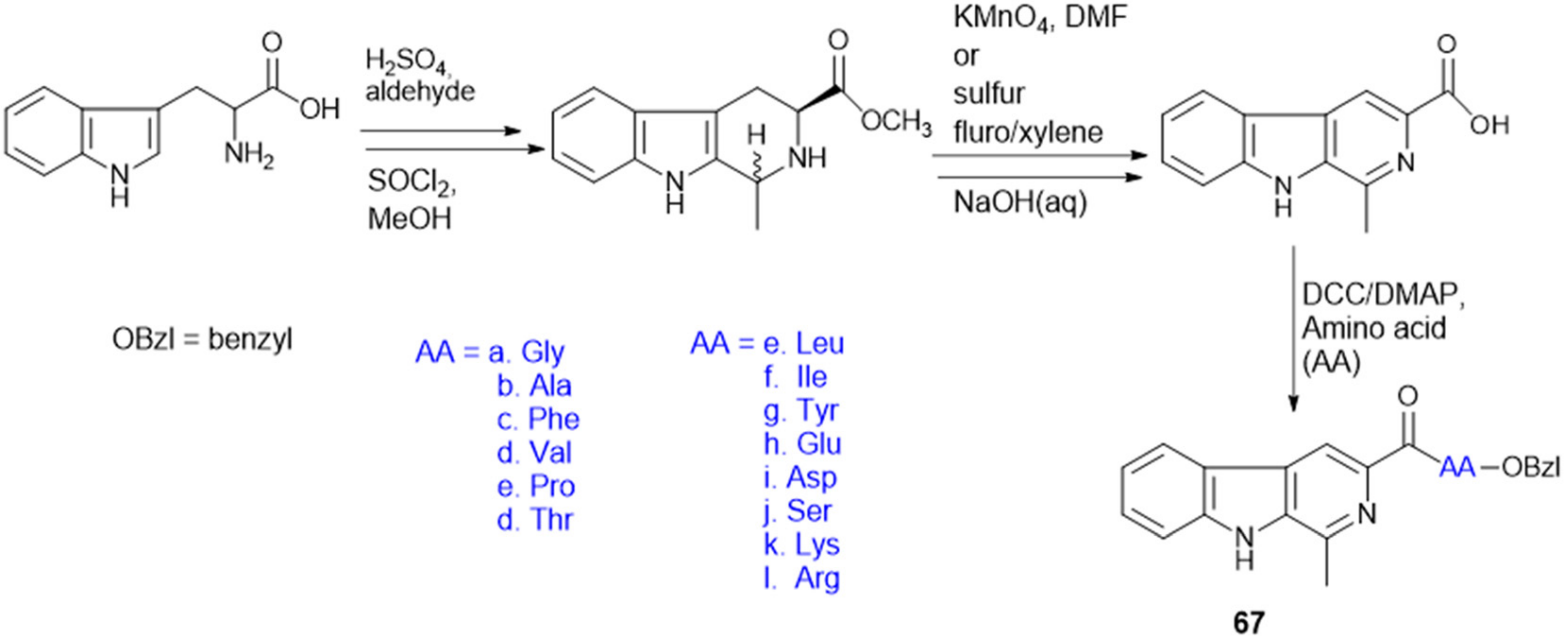
Synthesis of β-carboline derivatives of amino acid conjugates.

It is well known that many amino acids with functional side chains can interact with different types of DNA bases in base-specific ways. A series (Ala, Gly, Val, Phe, Thr, Tyr, Leu, Ile, Pro, Glu, Asp, Ser, Lys, and Arg) of β-carboline amino acid conjugates (compound 67; Scheme 26) have been synthesized, among which Lys and Arg conjugates were the most potent molecules as DNA binding agents with IC_50_ values of 4 and 1 μM against HeLa, MCF-7 and HepG-2 cells. Arg and Lys showed the highest membrane permeability (*P*_app_ (×10^−6^ cm s^−1^) = 2.01 and 1.97, respectively) in Caco-2 cells. Molecular modelling studies confirm that the guanidine moiety of Arg interacts with DNA and makes minor conformational changes.^182^

**Scheme 27:**
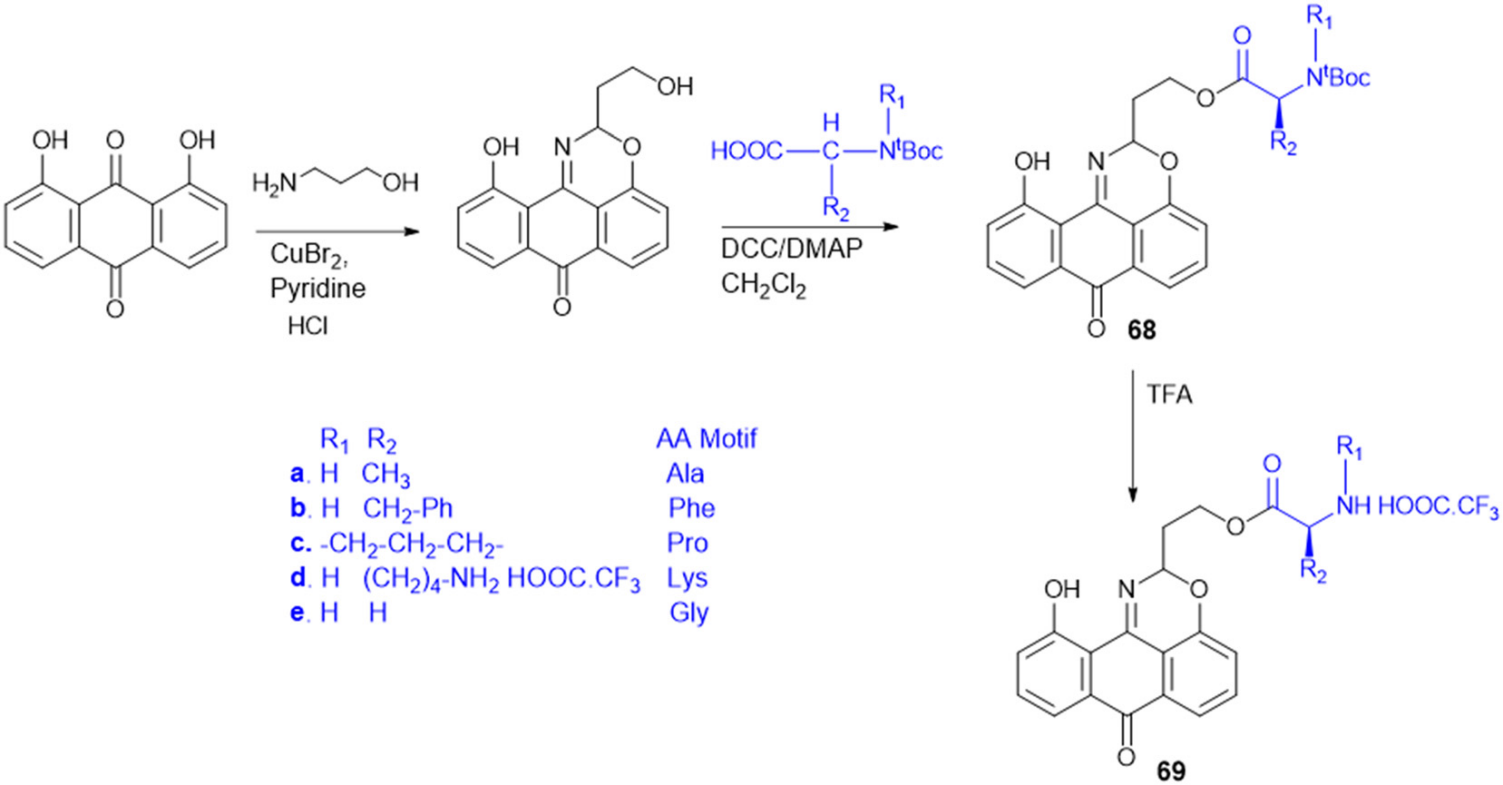
Synthesis of anthracene–amino acid conjugates.

N. Dias *et al.* synthesized a series of 1,8-dihydroxyanthracene-9,10-dione conjugated to an amino acid (68 and 69) Ala, Phe, Pro, Lys, or Gly (Scheme 27), which were studied for DNA binding and cytotoxicity. SAR studies suggested that addition of a neutral amino acid resulted in a substantially weaker DNA interaction while Lys addition with a cationic side chain significantly increased DNA binding; DNA binding seems to suffer with the introduction of a hydrophobic amino acid. The Lys conjugate showed staggering 20 times more cytotoxicity in human leukemia cells.^183^

**Scheme 28:**
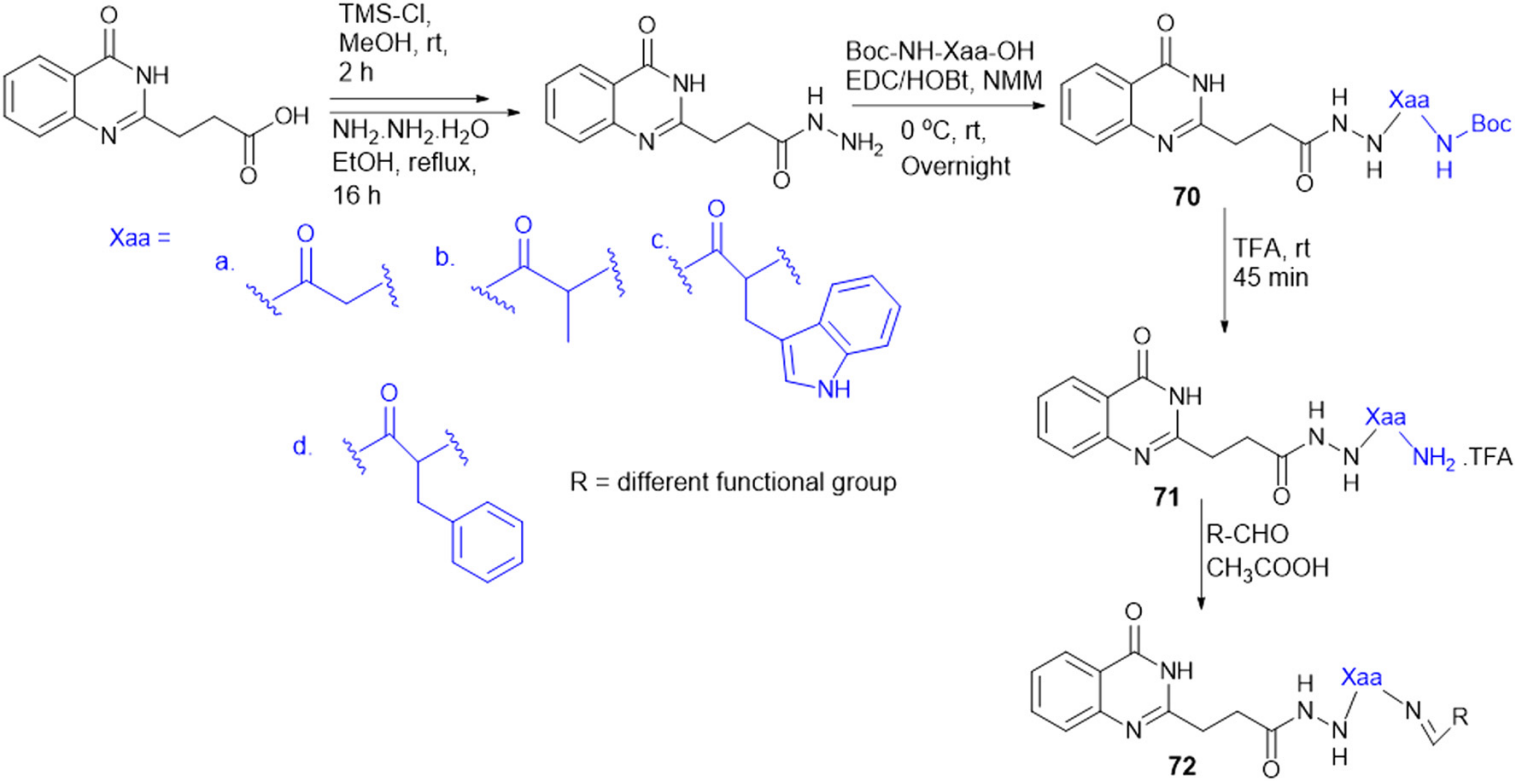
Synthesis of quinazoline amino acid conjugates.

A novel series of amino acid conjugated quinazolinone-Schiff's base (compounds 70, 71, and 72) were synthesized, which were potentially effective for DNA binding and exhibited anticancer properties against cancer cell lines MDA-MB-231, A549, and MCF-7. Trp and Phe, two hydrophobic and aromatic amino acids with an electron-donating group (–OH and –OCH_3_), were used to study the SAR. Trp and Phe conjugates showed more cytotoxic activity than Gly and Ala derivatives (Scheme 28). Thus, the aromaticity and hydrophobicity of Trp and Phe enhanced the anticancer activity. To synthesize quinazolinone hydrazides, 3-(4-oxo-3,4-dihydroquinozolin-2-yl)propanoic acid esterified with TMS-Cl/methanol was then reacted with hydrazine hydrate; the resulting hydrazide was conjugated with Boc-amino acids using coupling reagents like EDC and HOBt and a base like NMM. TFA deprotected the Boc group, and various substituted aldehydes (RCHO) were added using glacial acetic acid as a catalyst to obtain Schiff's base.^184^

**Scheme 29:**
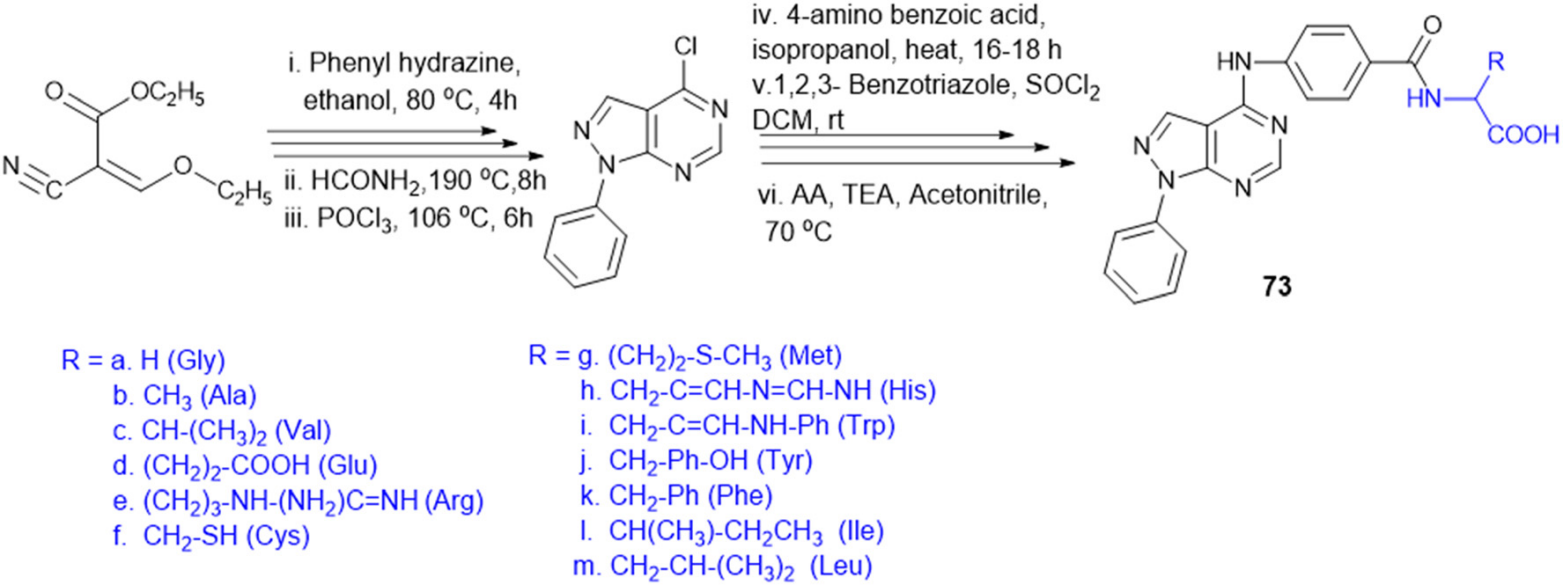
Synthesis of pyrazolo [3,4-*d*] pyrimidine amino acid conjugates.

Due to their structural similarity to MTX and MTX-related antifolates, a series of novel pyrazolo[3,4-*d*]pyrimidines with various amino acid conjugates were found to be effective antifolate agents. Various amino acid series are required to achieve a significant inhibitory level of the DHFR enzyme, to treat resistant cancer cell lines with classical antifolates, and to achieve superior DHFR inhibition activity; amino acids are conjugated based on their distinct hydrophilic or hydrophobic behaviour and aliphatic or aromatic nature. The synthesized molecules were evaluated for antiproliferative activity against HeLa, PC-3, HCT-116, BxPC-3, HepG2, and MCF-7 cancer cell lines. Among them, the molecule with the most potent anticancer activity found against the MCF-7 breast cancer cell line is pyrazolo[3,4-*d*]pyrimidine with an Arg amino acid (73e, Scheme 29) (IC_50_ = 4.65 mM) as compared to MTX. The pyrazolo[3,4-*d*]pyrimidine Arg conjugate 73e causes apoptosis by arresting the S-phase and induces pro-apoptotic protein expression (caspases and Bax) in MCF-7 cells.^185^

#### 7.4.3. Amino acid-based drug delivery *via* SLCs across the blood–brain barrier

**Scheme 30:**
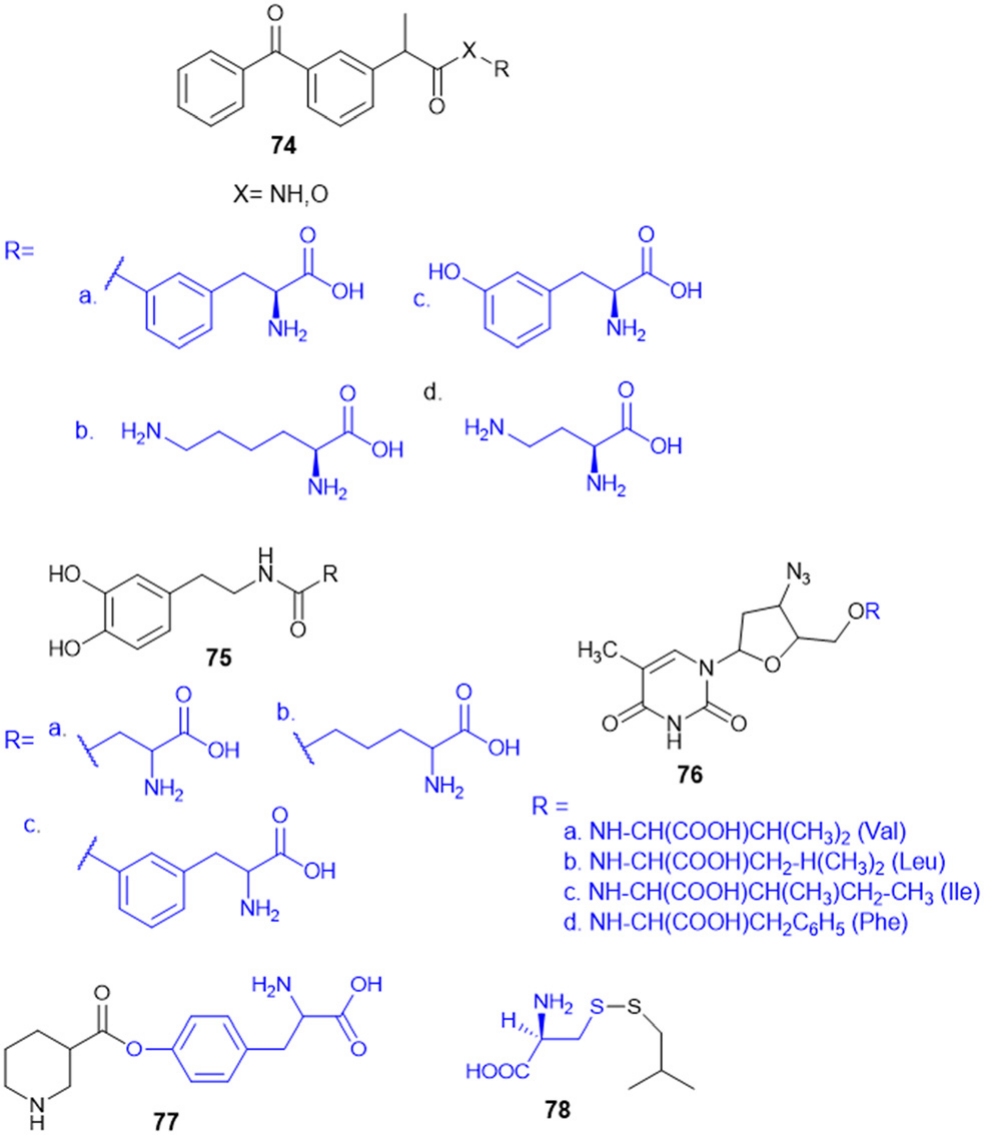
Amino acid drug conjugates.

LAT-1 overexpressed in the BBB and brain parenchymal cells supports selective drug penetration into the CNS, enhancing bioavailability of structurally compatible drugs. Using drug delivery of prodrugs improves ketoprofen (KPF) delivery for the treatment of neuroinflammation. Four KPF prodrugs (74, Scheme 30) targeted against LAT-1 were synthesized by esterifying or amidating KPF with aliphatic or aromatic amino acids and screened for cyclooxygenase (COX) inhibition and *in vivo* inhibition of prostaglandin E2 (PGE2) production. COX peroxidase activity assays showed that aliphatic amino acid-conjugated prodrugs performed as KPF derivatives (85% inhibition, IC_50_ ≈ 1.1 μM and 79%, IC_50_ ≈ 2.3 μM), whereas aromatic amino acid (Phe) ester prodrugs inhibited COX activity through bioconversion (90%, IC_50_ ≈ 0.6 μM), while amide prodrugs were not active. *In vivo* PGE2 quantitation confirmed efficient release of KPF from the amide prodrug, leading to significant PGE2 reduction in LPS-induced models of neuroinflammation, with greater metabolic stability and improved BBB permeability. The findings emphasize that LAT-1-mediated transport is a valuable method for brain-targeted delivery of NSAIDs, enabling better CNS penetration without elevating peripheral exposure, and has implications for treating neurodegenerative diseases such as Alzheimer's and Parkinson's disease.^186,187^ L. Peura *et al.* also chemically synthesized dopamine amino acid prodrugs designed for LAT1-mediated BBB transport, wherein Phe–dopamine (75c) conjugates were found to exhibit the highest affinity and brain uptake by LAT-1.^188^

SLC-mediated drug delivery confers a strategic advantage by avoiding efflux transporters like P-glycoprotein, thereby enhancing drug retention and CNS distribution. This is particularly valuable in the treatment of neurological disorders such as Alzheimer's disease (AD), Parkinson's disease (PD), epilepsy, schizophrenia, major depressive disorder (MDD), and brain tumors, where effective CNS drug delivery presents a key therapeutic challenge. Among the SLC transporters, the SLC1 family has APC that plays a critical role in high-affinity Na^+^-dependent glutamate transport, which is crucial for synaptic transmission and neuroprotection. Excitatory amino acid transporters EAAT1 (SLC1A3) and EAAT2 (SLC1A2) are responsible for clearing about 90% of Glu, preventing excitotoxicity and ensuring neural function. The transporters contain mutations which have been implicated in numerous neurological and psychiatric conditions, such as Huntington's disease, multiple sclerosis, dicarboxylic aminoaciduria, and episodic ataxia type 6. Although they hold clinical relevance, most SLC transporters have been poorly characterized, paving the way for increased understanding of their contribution to CNS drug delivery and disease etiology.^189^

Zidovudine (AZT) (76c, Scheme 30) is an anti-HIV agent; an isoleucinyl ester prodrug of AZT was synthesized to improve BBB permeability and brain targeting, exhibit similar or greater potency than AZT alone.^190^ Nipecotic acid, an inhibitor of neuronal and glial GABA uptake, is not effective as an anticonvulsant when administered systemically. To enhance its brain bioavailability, nipecotic acid was conjugated with glucose, galactose, and Tyr, and the nipecotic acid–Tyr (77) conjugate exhibited pronounced anticonvulsant activity through active BBB transport.^191^ Moreover, 6-mercaptopurine or 2-methyl-1-propanethiol (IBM) was conjugated to l-Cys (78) to develop a brain-targeted drug delivery system (BTDS) through LAT-1, a high-affinity transporter in cerebrovascular tissues that accepts a broad spectrum of drugs. Uptake experiments conducted in the presence of a competitive LAT-1 inhibitor, [14C]l-Leu, demonstrated that the IBM conjugate inhibited uptake by 92%, establishing LAT-1-mediated uptake.^192^

**Scheme 31:**
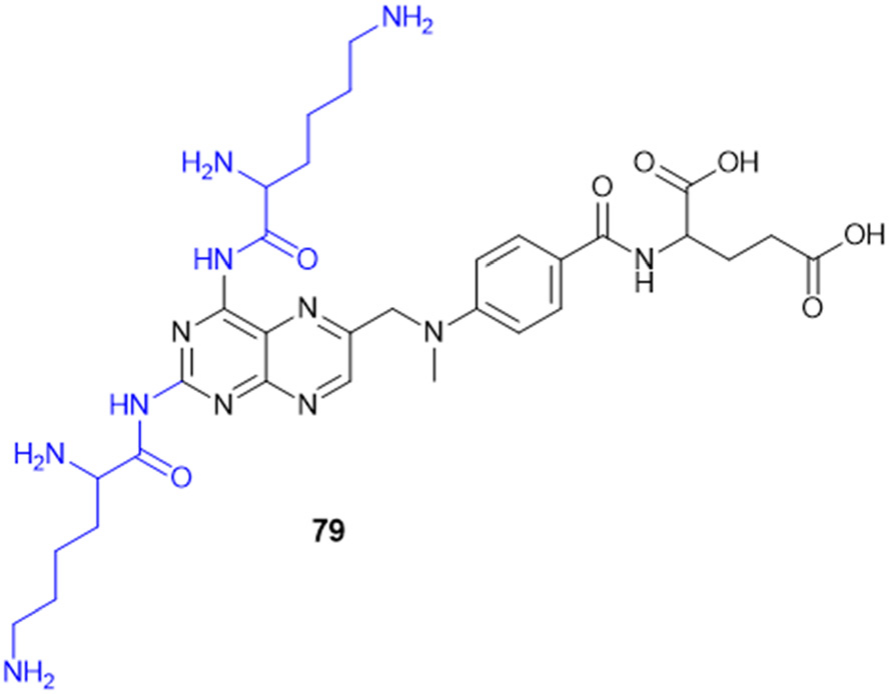
MTX–amino acid drug conjugate.

Methotrexate (MTX) is a chemotherapeutic drug with poor permeability across the BBB, making it unsuitable for brain tumor treatment. Thus, MTX was conjugated with l-Lys *via* an amide linkage, promoting the endogenous Lys transport system (LAT-1) at the BBB. Metabolic and stability studies suggested stable conjugation at physiological pH that slowly released MTX in the brain. The MTX–Lys conjugate (79, Scheme 31) showed promise in enhancing the brain delivery of MTX by improving its permeability across the BBB in albino mice with 2.81 μM kg^−1^ of treatment. The drug–amino acid conjugation approach leverages endogenous transport systems to expand the application of MTX for brain tumor treatment.^8^

#### 7.4.4. Amino acid-based drug delivery *via* SLCs in rheumatoid arthritis

Rheumatoid arthritis (RA) is an autoimmune disease characterized by chronic joint inflammation, which is primarily mediated by fibroblast-like synoviocytes (FLS) and immune cells such as T cells, B cells, and macrophages. Metabolic reprogramming in RA joints impacts glycolysis, pentose phosphate pathway, and lipid metabolism that supports abnormal FLS behaviour and immune dysregulation. Metabolomics studies in RA models highlight the metabolic changes important for regulating inflammation, immune cell function, and disease progression.^193^ Similar to their role in cancer, SLC transporters are involved in intracellular amino acid homeostasis and thus in maintenance of key physiological functions, including cellular metabolism, growth, and immune response. In RA, the FLS go through metabolic remodelling that is characterized by high glucose and other carbon sources like glutamine demand. It is facilitated through the upregulation of the amino acid transporters SLC38A1 and SLC7A5, which leads to increased uptake of glutamine. The increased glutamine metabolism promotes FLS proliferation, migration, and invasion, a feature of RA pathology. Concurrently, downregulation of SLC4A4, a bicarbonate transporter, also promotes an invasive phenotype by disrupting intracellular pH homeostasis.^194^ The convergence of SLC transporters' function in disease states highlights their value as therapeutic targets for metabolic modulation in disease states.

Chronic treatment of RA with DMARDs, steroids, and NSAIDs is frequently limited by the emergence of drug resistance. Recent insights revealed that dysregulated cellular metabolism is a key component of RA pathogenesis, more specifically driving aggressive FLS behaviour, macrophage activation, and immune cell infiltration. Such metabolic changes support chronic inflammation, proliferation, and invasion in the nutrient-poor synovial environment.^195,196^ As discussed previously, SLC transporters function as metabolic gatekeepers by controlling the influx of nutrients such as glucose, amino acids, vitamins, and ions. In RA-FLS, SLC-mediated transport is associated with unique epigenetic profiles in contrast to osteoarthritis FLS, which leads to disease-specific metabolic reprogramming. Parallel to cancer metabolism, emerging therapies can target or deliver drugs by SLC-mediated transporters to improve RA treatment and facilitate more precise patient stratification.

**Scheme 32:**
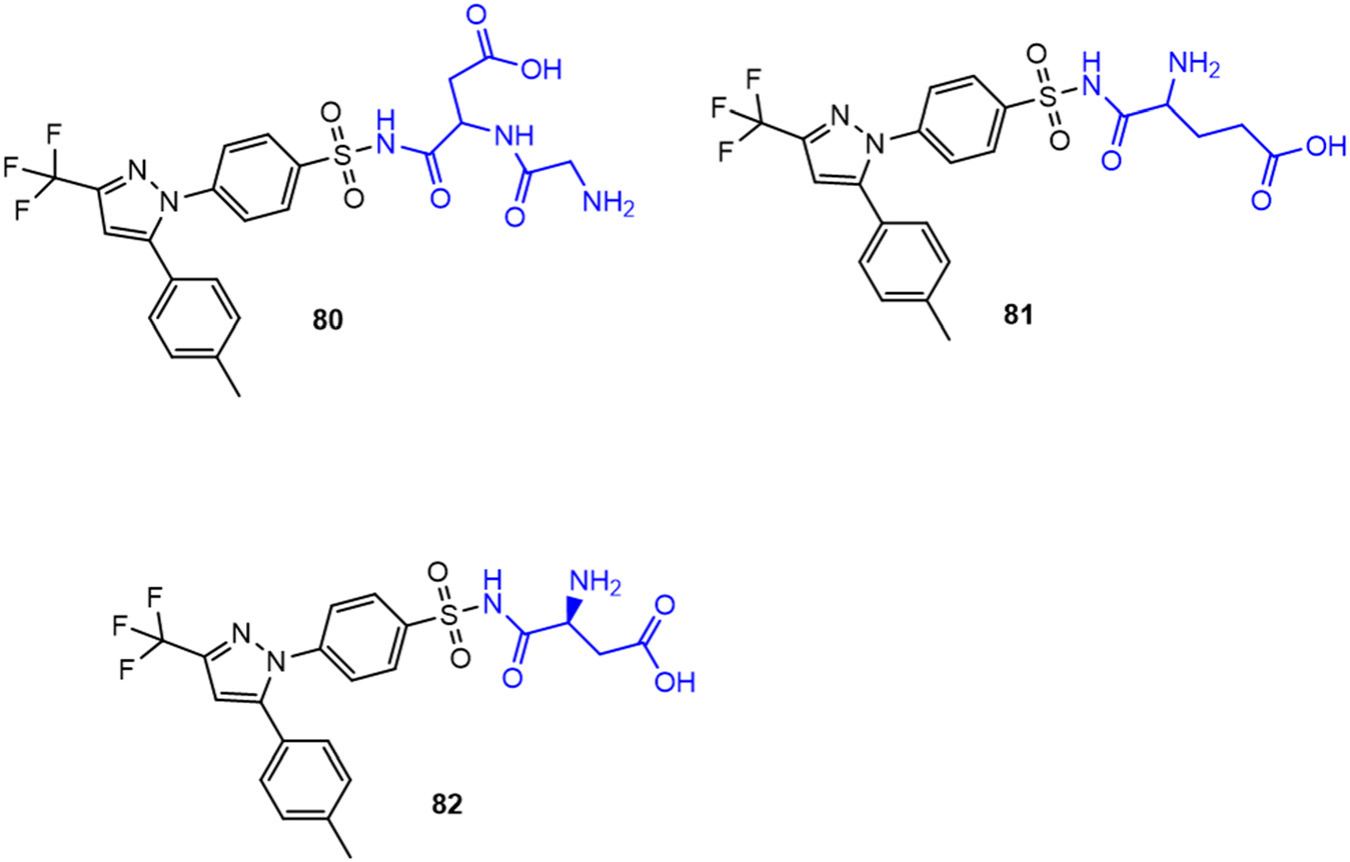
Celecoxib amino acid conjugates.

Recently, S. Cao *et al.* reported that l-Arg suppressed arthritis and inflammatory bone loss by remodelling osteoclast metabolism, converting energy production from glycolysis to oxidative phosphorylation, thereby raising ATP contents and interfering with TNFα-induced osteoclastogenesis. The metabolic imbalance results in increased inosine and hypoxanthine, critical inhibitors of osteoclast differentiation, thus decreasing joint inflammation and bone erosion. Findings suggest l-Arg as a potential dietary intervention for treating inflammatory arthritis.^197^ Y. Lee *et al.* synthesized a series of celecoxib–amino acid conjugates to counteract the pharmacokinetic limitations of the drug, including low oral bioavailability, non-linear absorption, and peak plasma concentration-related toxicity. The hydrophobic and selective COX-2 inhibitor celecoxib was conjugated with amino acids, *N*-glycyl-aspart-1-yl (N-GA1C) 80 (Scheme 32), glutam-1-yl (G1C) 81, and aspart-1-yl (A1C) 82, which were synthesized *via* amide bond formation between celecoxib's sulfonamide group and the carboxyl group of amino acids using EDC/NHS-mediated coupling. These modifications enhanced hydrophilicity and allowed better intestinal absorption with controlled systemic exposure. Among the conjugates synthesized, N-GA1C showed the most effective bioconversion to celecoxib in intestinal contents, with sustained plasma levels over 24 hours. The conjugates collectively enhance bioavailability and pharmacokinetic linearity, reduce toxicity, and extend anti-inflammatory activity compared to the parent drug.^198^

## 8. Miscellaneous

### 8.1. Amino acid as a linker

**Scheme 33:**
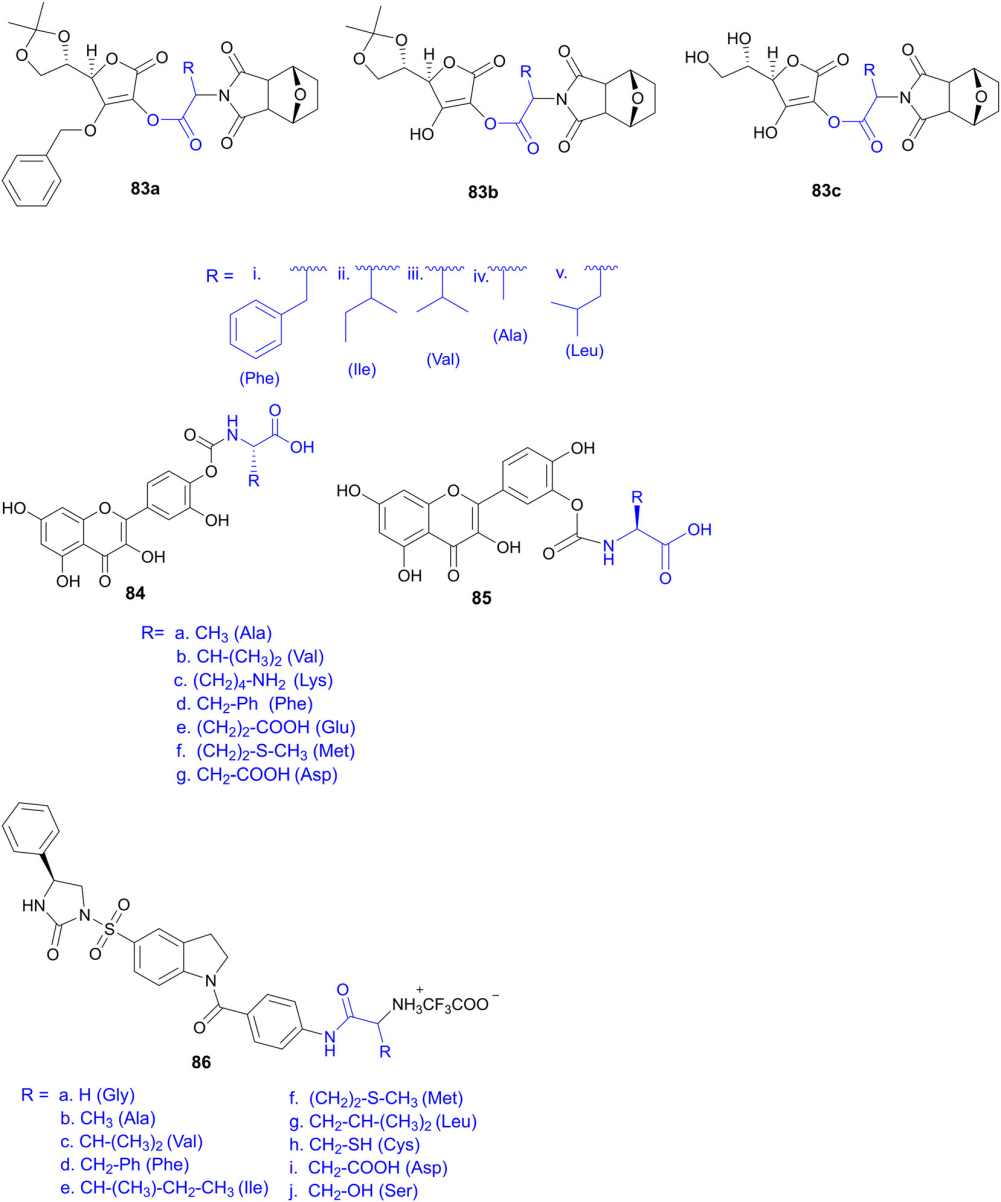
Amino acids used as linkers in prodrug design.

Besides bioconjugation, amino acids as linkers enhance pharmacokinetic properties. Forty-two norcantharidin–amino acid–vitamin C conjugates (compound 83, Scheme 33) were synthesized using amino acids as linkers screened in HepG2 and SW480 cells. Among them, several molecules showed high cytotoxicity. Additionally, one of the molecules 83a (iii) was found to induce Mφ-type macrophage polarization derived from mouse bone marrow.^199^ Quercetin (QUR) is a poorly soluble drug; thus, amino acids were used to enhance water solubility. Conjugates with various amino acids like Ala, Val, Phe, Met, Lys, Asp, and Glu and dipeptide conjugates of QUR (compounds 84 and 85) were synthesized to increase water solubility and decrease the rate of hydrolysis. Asp and Glu conjugates of QUR showed an increase in water solubility by 45–53 fold compared to the parent QUR molecule. In contrast, Lys and Glu conjugates showed resistance against hydrolase enzymes. Also, Glu and Asp and Ala-Glu conjugates enhanced cell permeability in MDCK (Madin–Darby canine kidney) cells compared to the QUR molecule. The QUR–Glu conjugate showed the most promising pharmacokinetic properties, with improved solubility, stability, and permeability compared to the parent drug quercetin.^200^

K.C. Lee *et al.* synthesized amino acid conjugates of (*S*)-1-[1-(4-aminobenzoyl)-2,3-dihydro-1*H*-indol-6-sulfonyl]-4-phenyl-imidazolidin-2-one (DW2282); eleven (Gly, Ala, Pro, Val, Phe, Ile, Leu, Met, Cys, Asp, and Ser) different hydrophilic and hydrophobic amino acids were used to enhance its water solubility. Anticancer activity was checked against the SW620 (human colon cancer) cell line. Compounds 86b, 86c, and 86d showed potent anticancer activity against the SW620 cell line compared to the parent molecules. *In vivo*, anticancer studies showed that compounds 86d and 86g exhibited remarkable tumor growth inhibition against the SW620 xenograft model. Compounds 86a, 86d, 86g, and 86f showed favourable reconversion rates compared to the parent drug in human plasma, suggesting that these can act as effective prodrugs.^201^

### 8.2. Steroidal amino acid bioconjugates

Steroids are a class of molecules with broad pharmacological activity and the capacity to bind membrane receptors and permeate biomembranes. A perhydrocyclopentano–phenanthrene ring structure with different degrees of functionalization distinguishes them. Hybrid bioconjugates incorporating steroid frameworks are recognized for their extensive biological activities.^202^ Cisplatin is an effective chemotherapeutic agent, although it is associated with considerable adverse effects. Conjugation with l-Met (87) and l-His has been studied to mitigate its toxicity (Table 5). These amino acids are essential in metabolizing platinum-based anticancer drugs. l-Met and l-His are employed as nephrotoxicity inhibitors of the cisplatin-creating complex. Further, different platinum(ii) complexes involving steroidal esters along with l-Met and l-His compounds were synthesized, exhibiting noteworthy cytotoxic activity against tumor cell lines that are non-toxic to normal cells.^203^

Cellular communication relies on the Eph receptors, a prominent family of receptor tyrosine kinases, and their ephrin ligands. Tumour vascularization and cellular dynamics during carcinogenesis are greatly impacted by the dysregulation of the Eph–ephrin system, which includes the EphA2 receptor. Thus, the Eph receptor–ephrin system is exploited as a potential target for novel anti-angiogenic drugs. According to recent research, lithocholic acid (LCA) is a tiny chemical that can block EphA2-dependent signaling in cancer cells. This suggests that the scaffold of (5β)-cholan-24-oic acid in LCA might be used to create more sophisticated EphA2 antagonists. Thus, a set of LCA derivatives were designed by attaching different α-amino acids (Gly, Ala, Val, Ser, Tyr, Trp, Met, Asn, and Phe) to their carboxyl groups. Analysis of the SAR reveals that attaining high potency requires a lipophilic side chain for amino acids. The most active of these derivatives was the l-Trp derivative (88, PCM126) (Table 5), which, at low micromolar concentrations, significantly outperformed LCA in terms of breaking the EphA2–ephrinA1 connection and decreasing EphA2 phosphorylation in prostate cancer cells. Among the most effective small-molecule antagonists of the EphA2 receptor is compound 88. The l-Trp conjugate is a functional pharmacological active molecule for therapeutic potential against Eph antagonists. Due to their hydrophobic side chain characteristics, Trp-based drug conjugates can thus be employed to develop potent amino acid conjugates that improve prodrug-like characteristics.^204^

Cholanic acid, identified through exploration of the SAR of lithocholic acid derivatives, emerges as a more potent inhibitor of EphA2–ephrin-A; to enhance both its inhibitory efficacy and physicochemical characteristics, cholanic acid is conjugated with various α- and β-amino acids as an EphA2 antagonist. SAR studies have shown that methyl ester amino acid conjugates are inactive, whereas free carboxylic acid-containing amino acid conjugates show better activity. This indicates that the free carboxylic acid group is crucial for the activity, the carboxylate group is fundamental to disrupting the interaction between the EphA2 receptor and ephrinA1 ligand, and β-Ala conjugated cholanic acid 89 (Table 5) shows the highest potency among all the amino acid conjugates of cholic acid.^205^

### 8.3. l-Amino acid use in nanoparticle fabrication

Selenium (Se) is a trace element found in the human body that is essential for a variety of physiological processes, including metabolism, DNA synthesis, reproduction, thyroid function, and defense against oxidative damage and infections. Se nanoparticles (SeNPs) have attracted attention for biological applications due to their lower toxicity compared to selenocompounds and other inorganic forms of selenium.^206^ However, their instability limits the potential application of SeNPs, since they tend to combine into grey and black elemental Se without any regulatory mechanisms. To overcome this challenge, amino acids have been used to change the structure of SeNPs, altering their physicochemical characteristics. Recent studies have focused on modifying SeNPs with proteins or polypeptides to improve their biocompatibility and therapeutic efficacy. SeNPs, for example, have been synthesised using a redox-based approach and functionalized with amino acids like Val, Asp, and Lys. *In vitro* cytotoxicity studies on MCF-7 have shown that amino acid-coated SeNPs greatly increased apoptosis rates, indicating their potential as promising therapeutic agents against cancer treatment. These findings present that amino acid-functionalized SeNPs might serve as a viable tool in targeted cancer therapy, with enhanced stability and lower toxicity than conventional selenium compounds.^207^

Two amino acid transporters, SLC6A14 (ATB^0,+)^ and SLC7A5 (LAT-1), have been targeted by lapatinib and JPH203-coloaded ATB^0,+^ targeted nanoparticles (LJ@Trp-NPs). Kou and colleagues developed poly-lactic acid (PLA) nanoparticles conjugated with Trp, Lys, and Gly for targeted delivery of lapatinib and JPH203 to cancer cells. Many cancer subtypes showed the overexpression of amino acid transporters SLCA14 (ATB^0,+^) and SLC7A5 (LAT-1). Cell viability studies indicate enhanced cytotoxicity for Trp-coated nanoparticles, likely due to increased nanoparticle uptake, as confirmed by cellular uptake studies. The enhanced endocytosis *via* the ATB^0,+^ transporter facilitates JPH203 release inside the cells and inhibits the LAT-1 transporter, further causing amino acid starvation and disrupting mTOR signaling. Also, *in vivo* studies showed that these nanoparticles increased lapatinib's half-life by four-fold and enhanced tumor biodistribution of DiR-loaded Trp-nanoparticles, resulting in synergistic anticancer activity with lapatinib.^208^ Functionalizing the nanoparticles with small molecules or ligands is one of the extremely promising strategies for effective and targeted delivery of the nanoparticles to tumor locations.^209^ Amino acid nanoparticle conjugates were employed in tumor imaging, such as MRI. While large amounts of amino acids are needed to maintain the rapid growth and proliferation rate, these up-regulated transporters can also be excellent targets for active tumor targeting. Alternatively, overexpressed receptors on tumor sites can be targeted with magnetic nanoparticles.^210^ The kynurenine pathway in the body metabolizes Trp, involving the enzyme indoleamine 2,3 dioxygenase (IDO), and LAT-1 regulates its delivery. IDO and LAT-1 overexpression was observed in various cancer types like ovarian, lung, and colorectal cancers.^211^

## 9. Limitations of amino acid conjugates

Amino acid conjugates face several translational challenges. One of the key issues is off-target effects, as SLC transporters are overexpressed under various disease conditions such as cancer and arthritis. However, as SLCs are expressed in healthy tissues as well, this leads to significant cross-reactivity, off-target effects, inadmissible uptake and toxicity. Interpatient variability such as genetic factors, environmental factors, physiological factors, and age complicates predicting the pharmacokinetics like uptake, distribution and response to drugs among the population. Overlapping substrate specificity and redundancy among SLC family receptors also lead to problematic pharmacodynamics. Overcoming these issues requires deeper understanding of transporter biology, real-time monitoring of patients' expression patterns and integration of personalized medicine into the design and clinical development of amino acid-based conjugates.^189,212^

Compared to ADCs, amino acid conjugates are small molecules that are relatively inexpensive to produce and can be synthesized through simple, well-established reactions such as amide bond formation or esterification, widely used in peptide and medicinal chemistry. This allows for large-scale manufacturing *via* a common chemical synthesis platform with reduced cost of manufacture and batch-to-batch consistency. In contrast, ADCs require complex biotechnological processes such as monoclonal antibody manufacturing in a mammalian expression system, site-specific linker conjugation of very potent cytotoxins, and verifying that the final product is homogeneous and stable, resulting in increased production and complexity.^213^

## 10. Future perspective

Rising technologies such as CRISPR-Cas9 gene editing and AI-assisted drug design are quickly transforming how molecules are being designed and screened. Current CRISPR technology enables creation of a cell line with a defined transporter profile, *i.e.*, the knockout of a transporter, which defines the amino acid uptake and elimination pathway and facilitates an enhanced structure uptake relationship. Genome-wide CRISPR activation and inactivation screening approaches, under nutrient restriction conditions, identify transporters essential for cell viability and nutrient uptake. Advanced CRISPR tools such as the base editor and prime editor enable scientists to make specific single-letter modifications in the DNA of a transporter or an activation enzyme. Technologies such as CRISPR-based genome editing are allowing scientists to edit individual amino acid transporters and prodrug-activating enzymes with greater precision than ever before, assisting in matching amino acid conjugates to their biological target more effectively.

Amino acid conjugates promise targeted drug delivery and improved metabolic profiles, but high dose or long-term effects of amino acids can show potential adverse effects such as gastrointestinal disturbances, imbalance of amino acid transporters, elevated ammonia levels and tumor promoting activity in sensitive cases (Gln and Arg). Long term pharmacokinetic studies are essential to see how amino acid conjugates distribute, metabolize and are cleared from the body. Since these therapies are progressing towards long-term or chronic use in patients, there is a requirement for continuous pharmacovigilance throughout clinical trials and post-marketing surveillance. Routine biomonitoring measures can identify adverse effects at early stages. The future of amino acid conjugates must also be shaped by environmental and economic sustainability. For the synthesis of greener alternatives, biocatalytic synthetic methods must be adopted while maintaining cost efficiency. Amino acid conjugates have the potential to become not only powerful therapies but also part of a more sustainable and ethically conscious future in medicine. To scale the production of amino acid conjugates by following enzymatic synthesis, greener chemical routes need to be developed.^213^

## 11. Conclusion

In conclusion, amino acids constitute a versatile and emerging strategy in drug design for enhancing drug delivery and therapeutic efficacy across a wide range of diseases such as cancer, bacterial, fungal, and viral infections, rheumatoid arthritis, and CNS disorders. The ability of amino acid conjugates to target specific receptors SLCs (PEPT1 and LAT1) further enhances oral absorption and tissue targeting. Their diverse use in the construction of therapeutic amino acid-based drug, peptide, and protein therapies offers a promising alternative for the treatment of a broad spectrum of diseases. Importantly, the stereospecificity of amino acid drug conjugates plays a critical role in pharmacokinetics, metabolic stability, and transporter selectivity, thereby directly influencing the biological activity. l-amino acid conjugates dominate current clinical development due to their biological compatibility, whereas d-amino acid conjugates offer resistance to enzymatic degradation, extended half-life and improved therapeutic outcomes, which highlights chirality as a powerful design principle in drug development. Clinically successful examples such as valganciclovir, valacyclovir and ALZ-801 underscore translational feasibility, while other molecules like brivanib–Ala, decitabine–Val and pomaglumetad methionil demonstrate the breadth of applications in oncology, infections and neurological disorders. Despite this promise, translation to the market remains limited due to interpatient variability in transporter expression and manufacturing complexity. Further advancement in amino acid drug conjugates will be driven by design strategies based on rational chirality and robust pharmacokinetics modeling, ensuring a streamline progression from preclinical research to clinical development and eventual market approval.

## Author contributions

Soma Mandal: conceptualization, methodology, visualization, writing – original draft, preparation. Rajat Choudhary: conceptualization, methodology, visualization, writing – original draft, preparation. V. Badireenath Konkimalla: conceptualization, supervision, resources, writing – original draft, writing – review & editing.

## Conflicts of interest

The authors declare that they have no conflicts of interest.

## Data Availability

There are no additional data available and no new data were generated or analysed as part of this review.
